# Magnesium isotopes of the bulk solar wind from Genesis diamond‐like carbon films

**DOI:** 10.1111/maps.13439

**Published:** 2020-01-22

**Authors:** A. J. G. Jurewicz, K. D. Rieck, R. Hervig, D. S. Burnett, M. Wadhwa, C. T. Olinger, R. C. Wiens, J. M. Laming, Y. Guan, G. R. Huss, D. B. Reisenfeld, P. Williams

**Affiliations:** ^1^ Center for Meteorite Studies Arizona State University m/c 6004 Tempe Arizona 85287 USA; ^2^ New Mexico Consortium 4200 West Jemez Road Suite 200 Los Alamos New Mexico 87544 USA; ^3^ School of Earth and Space Exploration Arizona State University Tempe Arizona 85287 USA; ^4^ Department of Geology and Planetary Sciences California Institute of Technology m/c 100‐23 Pasadena California 91125 USA; ^5^ GET‐NSA, LLC, AU‐62 19901 Germantown Rd Germantown Maryland 20875 USA; ^6^ Los Alamos National Laboratory (Remote Sensing) ISR‐2, m/s C‐331 Los Alamos New Mexico 87545 USA; ^7^ Naval Research Laboratory Space Science Division Code 7684 Washington District of Columbia 20375 USA; ^8^ Geological and Planetary Sciences California Institute of Technology m/c 100‐10 Pasadena California 91125 USA; ^9^ Hawaii Institute of Geophysics and Planetology University of Hawaii at Manoa 1680 East‐West Road, Post 504 Honolulu Hawaii 96822 USA; ^10^ Los Alamos National Laboratory ISR‐1 Los Alamos New Mexico 87545 USA; ^11^ School of Molecular Sciences Arizona State University Tempe Arizona 85287 USA

## Abstract

NASA's Genesis Mission returned solar wind (SW) to the Earth for analysis to derive the composition of the solar photosphere from solar material. SW analyses control the precision of the derived solar compositions, but their ultimate accuracy is limited by the theoretical or empirical models of fractionation due to SW formation. Mg isotopes are “ground truth” for these models since, except for CAIs, planetary materials have a uniform Mg isotopic composition (within ≤1‰) so any significant isotopic fractionation of SW Mg is primarily that of SW formation and subsequent acceleration through the corona. This study analyzed Mg isotopes in a bulk SW diamond‐like carbon (DLC) film on silicon collector returned by the Genesis Mission. A novel data reduction technique was required to account for variable ion yield and instrumental mass fractionation (IMF) in the DLC. The resulting SW Mg fractionation relative to the DSM‐3 laboratory standard was (−14.4‰, −30.2‰) ± (4.1‰, 5.5‰), where the uncertainty is 2ơ SE of the data combined with a 2.5‰ (total) error in the IMF determination. Two of the SW fractionation models considered generally agreed with our data. Their possible ramifications are discussed for O isotopes based on the CAI nebular composition of McKeegan et al. (2011).

## Introduction

### Science Behind SW Mg Isotope Measurements

The isotopic composition of the Sun is perhaps even more important than the elemental abundances of the Sun for understanding the formation and evolution of the solar system from the protoplanetary disc. Yet, we know little about solar isotopic abundances. Spectroscopic measurements of the solar photosphere have given us a few approximate isotopic measurements, but even solar physicists rely heavily on terrestrial proxies and solar wind (SW) from various returned samples (Asplund et al. 2009).

Thus far, the most definitive solar isotopic compositions have come from returned samples of SW (see Burnett 2013; Burnett et al. 2019). Yet, SW does not have the exact composition of the solar photosphere; solar processes that create the SW and accelerate it out of the photosphere fractionate the isotopes of at least some elements from those of the solar photosphere. The magnitude (‰) of the fractionation between the SW and the photosphere (herein denoted as ^x^Δ_(sw‐p)_ for element X) may—or may not—affect cosmochemical implications. For example, for SW N, the ^N^Δ_(sw‐p)_ of the SW to solar isotopic composition is small compared to the variations observed throughout the solar system. For other elements, such as SW O, the difference between the SW and solar isotopic composition is significant, and even a 6‰ error in ^O^Δ_(sw‐p)_ would change our interpretation of solar system evolution (Burnett et al. 2019).

So, quantifying solar physical processes that change the isotopic composition of the SW or, at least, having a generally applicable, empirically derived method of accurately determining the ^x^Δ_(sw‐p)_, can make SW‐derived isotopic data significantly more useful for cosmochemistry (and solar physics).

Mg isotopes in the SW are the key to understanding the science behind the ^X^Δ_(sw‐p)_ and to quantify the fractionation between the SW and the solar photosphere. The reason is that, except for CAIs. which often contain radiogenic ^26^Mg (e.g., Itoh et al. 2008), on the ≤1‰ level they are effectively unfractionated in all solar system materials (Catanzaro and Murphy 1966; Bizzarro et al. 2011). Therefore, the Mg isotopic composition of planetary materials represents the solar composition at the ‰ level and any deviation of the SW Mg isotopic composition ≥1‰ from this baseline is due to solar processes.

Solar physicists postulate that the processes that may cause fractionation of SW Mg isotopes occur in two regions of the Sun. First, there is a small fractionation in the solar convective zone. Specifically, although the solar composition is *essentially* static over the lifetime of the Sun, solar physics suggests that gravitational settling of heavy ions in the solar convective zone has produced an estimated ~3‰ per amu fractionation of Mg at the solar surface (Bochsler 2000; Turcotte and Wimmer‐Schweingruber 2002). Second, there is fractionation of SW during its formation and acceleration away from the solar surface. This significantly larger fractionation is still poorly understood; moreover, it probably changes with the source of the SW. For example, the predominant processes fractionating SW produced by coronal holes (fast SW) are likely to be different from those fractionating SW produced by coronal loops (slow SW). Knowing the precise composition of Mg isotopes in the SW for each SW regime would anchor solar physics models by direct observation. Once we understand the processes for fractionation of SW from solar matter (or at least define the trend), then we can accurately calculate the solar composition from the SW isotopic data for any element (see Burnett 2013; Burnett et al. 2019).

### Mass Fractionation in the SW: Previous Work in Solar Physics

The solar physics community has been researching the processes behind fractionation observed in SW ions relative to the solar photosphere using spacecraft data for decades. By 1982, spacecraft data had already indicated elemental fractionation of the SW composition from that of the solar photosphere (e.g., Geiss 1982) that correlated with the first ionization potential (FIP) of the element. Isotopes of the same element have the same FIP, so it is not a direct factor in fractionation of isotopes of the same element. Yet, at the time of the Genesis mission, spectroscopic measurements, spacecraft measurements, and the Apollo foils experiments had shown for decades that mass‐dependent fractionation did exist for isotopes (review in Bochsler 2007). Pilleri et al. (2015) and Heidrich‐Meiser et al. (2018) both find evidence that mechanisms other than FIP must play a role in SW elemental fractionation, with Pilleri et al. (2015) giving observational evidence for a mass‐dependent effect. The corollary to Pilleri et al. (2015) is that if a process fractionates elements by mass, then it also fractionates isotopes.

Mg data from in situ solar wind measurements have been used in the past to hunt for isotopic fractionation in the SW (necessarily mass dependent) based on the assumption of equivalence between solar Mg composition and that of Earth (e.g., Bochsler et al. 1997). Mg was also chosen for fractionation studies over more abundant elements (e.g., C, N, or O) because of the relatively large abundance of all three isotopes (Wiens et al. 2004). The in situ studies used data collected over up to 2 yr. Uncertainties of the results were mostly in the ±80‰ range or larger (Wiens et al. 2004), so potential hints of mass fractionation could not be easily confirmed, although some results (e.g., Kallenbach et al. 1998) showed lower uncertainties. However, trends indicated by different data sets did not always agree. That is, the Kallenbach et al. (1998) data seemed to indicate a definitive fractionation in a set including Mg, Si, and Ne isotopes favoring light isotopes in slow SW (~2‰ per amu). Conversely, other studies (Bochsler et al. 1997; Kucharek et al. 1997) found the opposite trend, but with larger reported uncertainties (see Wiens et al. 2004). When Bochsler (2007) suggested that the Apollo solar wind foil data on helium and neon, while ambiguous, were consistent with a Coulomb drag model (described in the Discussion), he indirectly acknowledged that sample return was necessary to obtain precision data. Laming et al. (2017) presented a quantitative model demonstrating how isotopic and elemental (primarily FIP) fractionation in the SW may be related, driven by magnetohydrodynamic waves whose interactions vary spatially and cause the variations in the SW fractionation and velocity with the solar source region observed by spacecraft (detailed overview in Laming et al. 2019). This last solar physics model relies heavily on a baseline SW composition determined from laboratory measurement of SW samples collected and returned to the Earth by the Genesis SW Sample Return mission.

### Genesis Mission and this Study

The goal of Genesis was to obtain a precise and accurate solar elemental and isotopic composition for use in cosmochemical models related to the formation and evolution of our solar system. To collect SW, the Genesis spacecraft used collectors of various composition and designed for the analyses of specific elements and isotopes (Burnett et al. 2003; Jurewicz et al. 2003). These collectors were placed in the trajectory of the SW ions, whose impact energies (averaging ~1 keV per amu) were enough to bury the ions half micron or less into the collector.

Because it was known preflight that there was some fractionation between solar composition and the SW that varied with solar source region, two instruments built by Los Alamos National Laboratory, the genesis ion monitor (GIM) and the genesis electron monitor (GEM), continuously monitored the SW. The monitors gave the exact conditions experienced by Genesis, enabling comparison of SW data from Genesis with that of other spacecraft and, in addition, allowed SW samples to be collected from predefined solar conditions (see Barraclough et al. 2003; Reisenfeld et al. 2007). That is, GIM and GEM data triggered deployment of specific arrays of collectors stacked below the bulk SW array in the Genesis Collector Array instrument (Burnett et al. 2003) to obtain SW from known solar features; that is, coronal holes, coronal mass ejections, interstream flow (Barraclough et al. 2003; Neugebauer et al. 2003). These specialized samples were to be used in large part to verify solar physics models; it was likely that one source of SW would eventually be identified as less fractionated from the nebular composition than bulk SW (see Reisenfeld et al. 2007 and Laming et al. 2017 for additional perspectives).

The crash of the Genesis Sample Return Capsule in 2004 made analyzing the SW sample more difficult and time consuming. The collectors were shattered and contaminated, so the development of new procedures is now required for the analysis of most elements and isotopes; that is, everything other than noble gasses and elements measured in the Genesis Concentrator (see Burnett 2013; Burnett et al. 2019). Still, some technique development would have been required even without the crash because some collector materials—like the diamond‐like carbon (DLC) on silicon (DoS) collectors used in this study—were not standard semiconductor materials, but were individually, laboratory‐made for Genesis.

This study uses secondary ion mass spectroscopy (SIMS) analysis to measure Mg isotopes of a bulk SW sample in DoS collectors from the Genesis B/C collector array (i.e., bulk SW—fixed arrays in both Burnett et al. 2003 and Jurewicz et al. 2003). A detailed description of procedures, including background information on why they were used, which may aid work by future investigators, is given below. Additional details of procedures are in the supporting information and a list of acronyms specific to this work are given in Table 1.

**Table 1:** Acronyms used frequently in this work

Abbreviation	Full phrase	Role	Definition	References giving additional information
DLC	Diamond‐like carbon	Part of DoS wafer that collected and retained SW	A carbon film having tribological properties more diamond than graphite. Genesis films were anhydrous, >50% sp^3^ bonds, dense (averaging ~3 gm cc^−1^), effectively homogeneous in columns but spatially variable electrical and chemical properties	Robertson (2002); Grill (1999); Sullivan et al. (1998); Friedmann et al. (1994); Jurewicz et al. (2017a, 2017b)
DoS	Diamond‐like carbon on silicon	SW collector	For Genesis, DoS wafers are DLC deposited on silicon using pulsed laser deposition by Sandia National Laboratory. The DLC collected the SW, the silicon supported the DLC film	Jurewicz et al. (2003)
DSM‐3	DSM‐3	Standard reference	Solution used by many laboratories for quantification of Mg isotopic composition	Bizzarro et al. (2011)
FIP	First ionization potential	Known to control concentration of element in SW	The energy required to remove the outermost electron from an atom	Reisenfeld et al. (2007); Laming et al. (2017)
ICD	Inefficient coulomb drag	Physics model for SW fractionation	A model based on SW H interacting electrically with minor SW ions; that is, as SW H moves, coulomb forces (modified by the SW environment) drag minor SW ions in the direction of H movement. Because this is a mass‐dependent process, it fractionates isotopes; however it is not considered significant for elemental fractionation	Bodmer and Bochsler (1998); Bochsler (2000)
IMF	Instrumental mass fractionation	Fractionates isotopes during analysis	Electromagnetic fields inside all analytical equipment tend to separate isotopes of the same element so that one isotope is measured more efficiently than the others. In SIMS, IMF is affected by sample and electrical fields. This fractionation is mass dependent and is accommodated using an appropriate standard (see IMF standard)	Wilson et al. (1988); Eiler et al. (1997)
IMF Standard	IMF standard	DoS with known ^25^Mg/^26^Mg is used to correct IMF	Primary standard made for this study. Commercial implant of ^25^Mg and ^26^Mg into both DoS and silicon; then ICPMS performed by dissolution of silicon determines ^25^Mg/^26^Mg ratio precisely so that analysis can be used to correct for IMF when measured under the same conditions as the unknown (SW). Also: IMF‐Calibration standard; DoS standard; implant standard	Burnett et al. (2015); Jurewicz et al. (2017a, 2017b)
SoS	Silicon on sapphire	SW collector	One of several solar wind collectors flow in the Genesis Array instrument. This collector is a single‐crystal silicon film epitaxially grown on sapphire: the film collects and retains the SW while the sapphire supports the film	Jurewicz et al. (2003)
SRIM	Stopping range of ions in matter (program)	Program used to model implants	Freeware used internationally for both teaching and research concerning ions implanted into solids. Assumes homogeneous target material not previously damaged	http://www.srim.org
SRIM fit	Model for implant generated using SRIM (program)	Distinguishing implant from surface contamination	Best fit (using Ψ^2^) between SW measured versus calculated from spacecraft measurements	Supporting information Table A3; Jurewicz et al. (2019)

## Experimental: Materials and Techniques

### The SW Collector

The selection of the appropriate collector for Mg analysis by SIMS was based on *in‐house* testing of several collectors (e.g., Burnett et al. 2007) and balanced a number of factors, including electrical conductivity, SW retention, ease of cleaning, and low Mg background. The ability to measure the SW close to the collection surface was also a factor because the depth of implantation of SW changes with the mass of the ion (*E*_implant_ = 0.5 × m × *v* ^2^). Because the velocity distribution for each element in the SW depends overwhelmingly on solar processes and conditions (not mass), heavier isotopes are implanted deeper than light isotopes. Therefore, the isotopic ratio changes sharply in the 10 nm adjacent to the collection surface. Thus, the closer to the collector surface that the SW can be cleanly measured, the more accurate the final isotopic measurement (Koeman‐Shields et al. 2018).

Previous analyses of the SW Mg fluence had used multiple types of silicon collectors (both Czochralski grown [CZ] and float zone [FZ] silicon wafers and silicon on sapphire [SoS] wafers) as well as DLC film on silicon (DoS) wafers (Jurewicz et al. 2008). The silicon of the SoS wafers was insulating and required a conductive coating for SIMS analysis. The low conductivity suggests very high intrinsic purity, but coating was a process that might add Mg contamination to the surface. Moreover, there were at least some physical and/or chemical changes to flown SoS (e.g., either extremely radiation damaged and/or oxidized during flight or the crash) as Humayun et al. (2011) discovered (during a technique development study for measuring SW Mg isotopes) that flight SoS coatings dissolved in aqua regia even though their flight‐spare counterparts did not.

Silicon collectors are more conductive than SoS, but are also difficult to clean. Silicon is chemically reactive, and some crash debris could not be removed (e.g., Callaway et al. 2009; Goreva et al. 2015; Welten et al. 2018). In fact, because of difficulty cleaning the collector surface, the method of choice for measuring SW in Genesis silicon by SIMS is depth profiling from the backside (Heber et al. 2014). But the backside depth profiling technique does not cleanly sample SW proximal to the collection surface because any slight sample wedging causes a differential breakthrough (see Heber et al. 2014). Techniques for deconvolution of the data near the surface are currently under development (e.g., Koeman‐Shields et al. 2018). But, these techniques were not available at the time of the analyses used in this study, nor was the method of removing the silicon backing from the DLC (Rieck 2015).

DoS wafers are chemically inert and can withstand aggressive cleaning without damaging the surface of the wafer. SW Mg profiles obtained by front‐side SIMS analysis using an O_2_^+^ primary beam in the DLC appeared to be separated from almost all ion‐mixed surface contamination within ~4 nm of the surface. Moreover, transient sputtering effects in DoS standards were shown to be 4 nm or less for the SIMS conditions used here (Jurewicz et al. 2017a, 2017b). For comparison, observations during SIMS analysis of silicon under similar conditions produced transient sputtering zones of 8–12 nm. Accordingly, this study used DoS collectors.

### The Calibrated Standard Implant for Instrumental Mass Fractionation Corrections

Instrumental mass fractionation (IMF) of the Mg isotopes during SIMS analysis was determined using a flight‐spare DoS fragment implanted with both ^26^Mg and ^25^Mg, each at nominally 1 × 10^14^ ions per cm^2^. Burnett et al. (2015) give a method of making and calibrating the analytical standards using commercial implants into materials of known composition. A modification of that method was used here to make and calibrate an IMF standard for SW Mg isotope measurement.

**Table 2:** Backgrounds: Analyses of the IMF standard (cps)

	^24^Mg	^25^Mg	^26^Mg
STD_2	0.5510	1.166	1.139
STD_3	0.1555	0.0744	0.0372
STD_4	0.0895	3.518	3.770
STD_5	0.0239	0.0582	0.0865
STD_6	0.0597	0.070	0.1401
STD_7	0.0388	0.0134	0.0224

Gray shading indicates the presence of correlated interferences.

**Figure 1:**
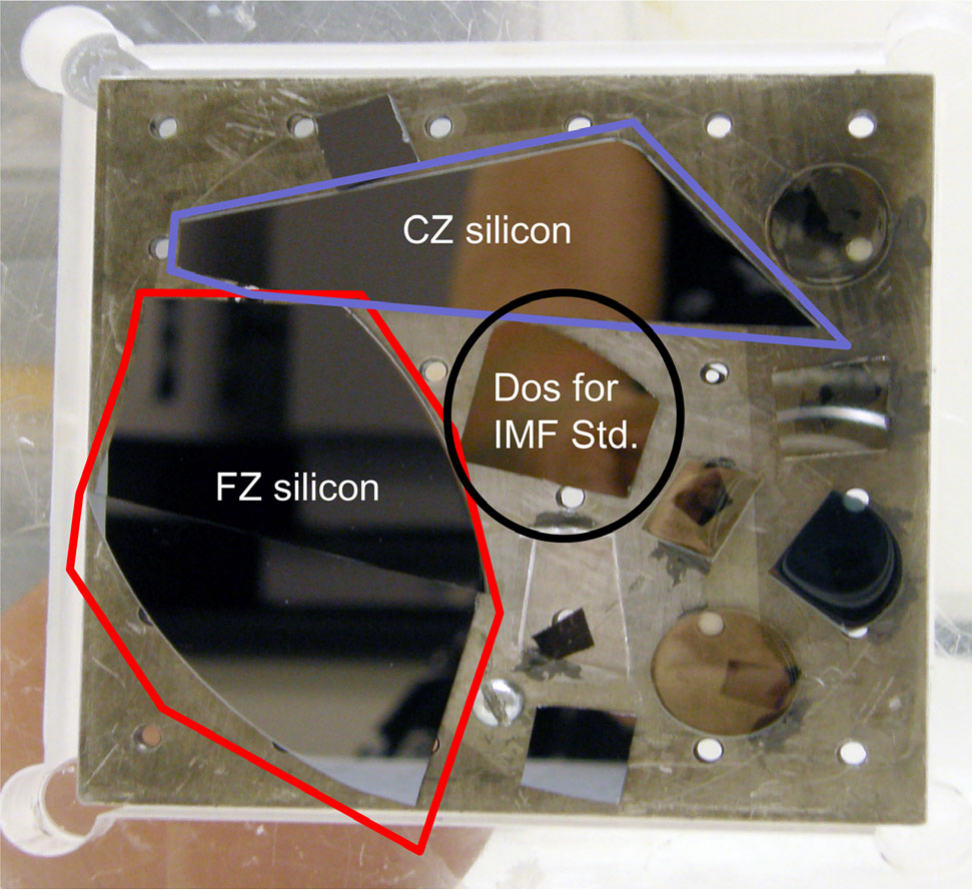
Materials mounted for implantation with ^25^Mg^+^ and ^26^Mg^+^. Reported implant fluences are nominal. However, because the implant is uniform across all materials on the plate, the absolute ^25^Mg^+^/^26^Mg^+^ ratio was subsequently measured by ICPMS using the Genesis flight‐spare CZ silicon (outlined). ICPMS results for the FZ silicon which was *not* Genesis flight‐spare (outlined) had scatter suggesting embedded particulates so results were not used. The SIMS IMF standard (also referred to in the text as the calibrated standard implant) is the circled DoS wafer. (Color figure can be viewed at http://www.wileyonlinelibrary.com.)

Accurate determination of IMF required an independent, precise calibration of the (nominally 1:1) ^26^Mg/^25^Mg ratio. Accordingly, the Isotope Cosmochemistry and Geochronology Laboratory at ASU used ICPMS to determine the absolute isotopic composition of the implanted Mg. DLC is not amenable to dissolution and so is not analyzable using ICPMS. Therefore, pieces of FZ and CZ silicon were co‐implanted with the DoS standard (Fig. 1) and it was the silicon surrounding the DoS that was analyzed in the ICPMS calibration. Homogeneity of the implant was not an issue: RBS on a high‐dose Fe implant from the same vendor (Leonard Kroko Inc., Tustin, CA) showed the Fe implant to be uniform within our ability to measure (~≤1%). Sample preparation for the ICPMS analysis is given in supporting information Table A1.

The ICPMS measurements of the implanted silicon were bracketed by replicate analyses of an ICPMS standard, DSM3, whose composition was reported by Bizzarro et al. (2011) to be ^25^Mg/^24^Mg = 0.12691 and ^26^Mg/^24^Mg = 0.13969, which is nominally ~0.5‰ heavier than measured terrestrial values (Catanzaro and Murphy 1966; Bizzarro et al. 2011).

Leonard Kroko Inc. was not able to separate ^25^Mg from ^24^Mg^1^H completely, so a small amount of ^24^Mg was also implanted. This “accidental” implant was measureable by SIMS (^24^Mg/^25^Mg = 0.00423) in a piece of silicon remaining from the implant and subtracted from the ^24^Mg the ICPMS data; the remaining ^24^Mg was the analytical blank. Assuming the blank isotopic composition to be the same as DSM‐3, the implant ^26^Mg/^25^Mg was determined to be 1.0224 ± 0.0004 (2 sigma). This error is the reproducibility of replicate ICPMS analyses; the SIMS error is negligible for this calculation. The absolute error for the DSM3 standard is not included. *Thus, the measured SW Mg isotopic ratios reported in this work are relative to DSM3 as a representative sample of terrestrial Mg*.

### The SIMS Analysis

Analyses were run on the ASU CAMECA IMS 6f in depth profiling mode. A 22nA O_2_^+^ primary beam having an impact energy of ~7.5 kV was rastered over a 250 × 250 μm^2^ area. The resultant sputtering rates were ≤0.03 nm s^−1^. Sputtering rates were calculated using the depth of the analysis pit as measured by an Alpha Step 200 profilometer divided by the total analysis time. A standard CAMECA 9‐hole mount held both the calibrated implant used for determining IMF and the flight sample (Genesis #20732,2). To avoid nonuniform extraction fields, depth profiles were run at least ~1.3 mm from the edge of the holes in the cover plate of the mount. Both IMF standard and the flight sample were treated identically.

**Figure 2:**
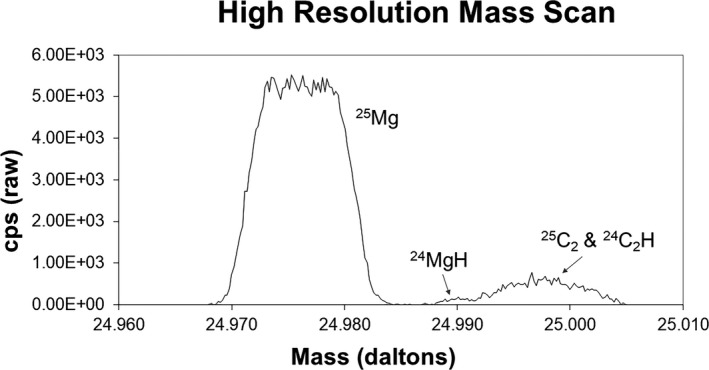
High resolution mass scan (MRP ~3550 M/ΔM) for a ^24^Mg, ^25^Mg, ^1^H implant. Nominal fluences were 5e13 atoms per cm^2^ implanted at 43 keV for ^25^Mg and ^24^Mg; 1.8e16 atoms per cm^2^ implanted at 10keV for ^1^H. Mg is *not* implanted to imitate the natural abundance ratio, so the tiny hydride peak on the low‐mass edge of the carbon interferences represents ~3‰ ^24^MgH. The value ~3‰ ^24^MgH was assumed for later data reduction, then the MRP was lowered to 1604 M/ΔM for the analyses. The lower MRP allowed the collection of significantly more SW Mg counts per profile.

All analyses used DTOS (the CAMECA Dynamic Transfer Optical System in which charged plates deflect the secondary ions emitted at each position to a single focal point) with 60% electronic gating (i.e., the outer perimeter equaling 20% of the raster width on each side does not collect secondary ions), so that the 250 × 250 μm^2^ raster gave a 150 × 150 μm^2^ analyzed area. However, even with 60% DTOS, high‐resolution mass scans did not give perfectly flat peaks because our focused, primary O_2_^+^ beam had a “wing.” Therefore, a 750 μm diameter field aperture was used to mask the DTOS crossover point in the image plane. The field aperture did not change the analyzed area; rather, it precluded transmission of scattered, extraneous secondary ions to insure that high‐resolution mass scans produced flat‐topped peaks. The mass resolving power (MRP) was ~1604 M/ΔM (i.e., at mass 24, there was at least a 90% separation of ^24^Mg from ^12^C_2_) because the high‐resolution mass scan on a ^24^Mg‐^25^Mg‐H implant at ~3554 M/ΔM indicated that ^24^Mg‐H formation was minor (~3‰; see Fig. 2). Because the SW fractionation was hypothesized to be significantly larger than 3 ‰ per amu (e.g., ~10‰ and 20‰ for ^25^Mg/^24^Mg and ^26^Mg/^24^Mg, respectively, for the Coulomb Drag model of Bodmer and Bochsler 2000; and a similar range from Laming 2017 Model 2—both described in the Discussion section), the additional statistical error for ^25^Mg caused by degrading the MRP to include ^24^Mg‐H was accepted and 3‰ was subtracted after the analyses to account for the interference. In return, the gain in the number of Mg counts at the lower MRP decreased error for counting statistics on all Mg species (Rieck et al. 2010).

These SIMS parameters (above) gave ^25^Mg and ^26^Mg signals on the IMF standard that were optimized to give the maximum counting rate that did not require a dead time correction for the detector, a correction that would have added error to the data reduction. Specifically, the signal at the peak of the calibrated implant was ~4 × 10^4^ cps (counts per second) after correction for “instantaneous count rate” (i.e., the highest counts seen by the detector, not the average counts reported for the entire rastered area (as per Wilson et al. 1988). SW Mg was ~2 orders of magnitude lower in fluence and dead time corrections were never an issue. Yet, counts were sufficient to give (^25^Mg cts/^24^Mg cts) and (^26^Mg cts/^24^Mg cts) ratios (found by the integral of each ion species) having a typical statistical error of ~6 to 7‰ (±1ơ).

A single electron multiplier was used and signals for secondary ion signals were collected sequentially by mass (peak) jumping. Time lost to peak jumping in the SIMS duty cycle was not an issue for the 75 kV implant because the intense ^25^Mg and ^26^Mg signals provided excellent statistics (<1‰). Secondary ions for five species (^24^Mg, ^25^Mg, ^26^Mg, ^12^C^+^, and C_2_^+^) were collected throughout the depth profile. In contrast, the SW fluence was two to three orders of magnitude lower than that from the implant, so the time lost peak jumping had significant impact on the total SW signal. Accordingly, the depth profiles for the SW collector measured the ^12^C and C_2_ only at the beginning and end of each profile, thus allowing more time to measure Mg ions. This decision assumed implicitly that the DLC matrix was homogeneous in columns, as reported by Sullivan et al. (1998).

The ^12^C^+^ and C_2_^+^ signals from the Genesis collector (hereafter designated “initial” and “final”) were collected for a range of times. The majority of both initial and final matrix‐ion measurements were 25 s each, but the range was ~18 to 270 s. Since ~25 s of sputtering at a nominal sputtering rate of 0.03 nm s^−1^ consumes only ~0.8 nm of sample, depth losses due to the initial and final sputtering were usually within the error of the measurement of the depth of the analysis pit. For extended initial and/or final matrix collections, the times recorded in the Mg profiles were corrected to account for the additional sputtering of the matrix.

While the SIMS analytical conditions for the standard were the same as for the Genesis‐flown DoS (except for the matrix‐ion collection), the duration of SW analyses was much shorter. Because the modal value for the energy of SW Mg implantation was ~30 kV, the peak depth was ~25 nm and each SW depth profile took about ~2 h to run. In contrast, the monoenergetic implantation of ^25^Mg and ^26^Mg for the standard was 75 keV, the peak was ~90 nm, and each depth profile in the standard ran ≥7 h. During this session, the nominally 22 nA primary‐beam current appeared to be stable. Primary current drift (from current recorded at the beginning and end of each profile) was <4 nA for STD_4 and <2 nA for the other profiles. The SIMS operators also conducted random spot checks of the readout of parameters during those long analyses and did not observe excursions in the primary beam intensity.

### The Novel Method of Data Reduction that Accommodates the Matrix Effects

Data were collected for this work in 2009 during a technical development study of SW quantification by SIMS analyses of DoS collectors. The choices of SW collector and the SIMS analytical conditions (above) implicitly assumed that any variations in the DLC film are small and due to spatial differences in carbon texture and structure. Instead, early data from this study indicated that the lateral heterogeneity in the structural and electrical properties previously reported for DLC (e.g., Friedmann et al. 1994; Sullivan et al. 1998) radically affected SIMS results. Even more has been learned about the reduction of SIMS data since Jurewicz et al. (2017a, 2017b). For example, fig. 15 of Jurewicz et al. (2017a, 2017b) shows a ~10% offset in relative sensitivity factors (i.e., Mg_concentration_/[Mg_ion yield per C_] between SW Mg and their calibrated standard (our IMF standard). One hypothesis for that offset was that SW H affected the SIMS ion yields. Instead, *in‐house* work has shown that the surface correction of Jurewicz et al. (2017a, 2017b) left some ion‐milled surface contamination in the SW data: although a small number of counts, the effect on the fluences was significant because the SW is present in trace concentrations. Nevertheless, the effect of variations in the DLC structure and composition on SIMS analysis of Genesis DoS collectors reported by Jurewicz et al. (2017a, 2017b) still apply. The need to accommodate these matrix effects in isotopic analyses shaped the data reduction technique used herein.

#### Matrix Effects and their Causes in DLC

Isotopic analyses (and fluence analyses) by SIMS can be skewed by matrix effects both directly and indirectly. Changes in electrical conductivity in this material have caused variations in the IMF by 2% per amu (not ‰ per amu) among individual isotopic measurements in this study. Second, matrix effects cause spatially variable changes in the oxidation state of the sputtered material (as evidenced by the variable ion yields reported in Jurewicz et al. 2017a, 2017b). Oxidation state also controls the amount and type of hydrogenated ions produced and, therefore, cause both the type and intensity of mass interferences to be spatially variable (again, at % levels, not ‰ levels, in the hydrogen‐implanted SW samples). There is a dearth of reference materials for *quantitative* elemental analysis in DLC by SIMS in the literature, presumably because most DLC is used to reduce friction or increase the wear resistance of parts (Robertson 2002; Chu and Li 2006) and the trace element chemistry of DLC is, in general, not a commercial issue. Therefore, an overview of these matrix effects is given here; details are in the Results section and the SOM.

Obvious sources of heterogeneity in DLC include entrained particulates (dust, or bits of metallic silicon) and/or contamination at annealing surfaces. Large particulates skew the profile shape; smaller particulates are difficult to detect and remove but may still require the exclusion of the affected depth profiles from the data set.

**Figure 3:**
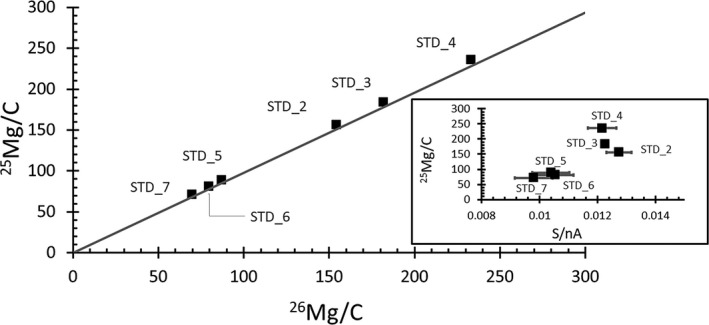
Illustration of variable ion yields, where markers are the total counts from the depth profile divided by the average carbon counts per sec. The standard is implanted uniformly and the statistical uncertainty for each analysis is smaller than the markers. Normalization to the average matrix ^12^C^+^ counts to mitigate variations induced by changes in primary beam current and/or changes in the current‐normalized sputtering rate (S/nA) caused by variations in matrix properties (e.g., inset). If there were no IMF, variable sputtering yields would plot on a line with a slope equal to the ICPMS‐derived ratio of ^25^Mg/^26^Mg for the implant (dark gray line). Data would be a single cluster in the main plot and also in the inset if DLC were homogeneous.

Silicon is a contaminant in even the purest carbon sources (e.g., Canada Carbon Inc. 2016), and the minor silicon intrinsic to the DLC matrix is heterogeneously distributed and has significant effects on the SIMS analysis of DLC. In the absence of significant concentrations of silicon, sputtering with an O_2_^+^ beam is controlled by chemical etching (Guzman de la Mata et al. 2006; Rubshtein et al. 2006). When significant silicon is present, chemical etching no longer dominates and DLC sputters more slowly under the O_2_^+^ beam (Jurewicz et al. 2017a). The slower sputtering rate suggests the formation of a “barrier” SiO_*x*_ surface film that protects the carbon from etching; that is, the sputtering mechanism becomes “typical” and includes more ion mixing that allows oxygen from the primary beam to build up in the matrix, increasing the secondary ion yields (Williams and Baker 1981). The matrix normalized, variable ion yields of Mg^+^ ions were observed by Jurewicz et al. (2017a, 2017b). The spatially variable ion yields in DLC are illustrated in Fig. 3, where they are described by the line:

(1) $${}^{25}{\text{Mg}}_{\mathrm{sims}}={\left({}^{25}{\text{Mg}}^{26}\text{Mg}\right)}_{\mathrm{icpms}})/\left(\frac{\varepsilon }{1000}+1\right)\times^{26}{\text{Mg}}_{\mathrm{sims}}$$

where ^x^Mg_sims_ is the ion yield of Mg species X (^25^Mg/^26^Mg)_icpms_) is the ICPMS‐measured ratio of ^25^Mg/^26^Mg implanted into the standard, and ϵ is the IMF. The line shown in the plot has the slope of the ICPMS‐determined Mg ratio (i.e., ϵ = 0). A constant, nonzero ϵ changes the slope while a variable ϵ curves the line. In Fig. 3, the data are linear within error; however, markers for depth profiles 2, 3, and 4 are closer to the line than depth profiles 5, 6, and 7. Part of the observable shift in slope from ϵ = 0 is due to the %‐level IMF of the sample, but the shift for profiles 5, 6, and 7 is due to the fact that the IMF is relatively larger in magnitude for these three analyses; that is, analyses labeled “silicon‐rich” in Jurewicz et al. 2017a, 2017b).

Silicon also appeared to affect the length and shape of the zone of transient sputtering (Jurewicz et al. 2017a, 2017b) consistent with Williams and Baker (1981), which was longer when the sputtering per nA of beam current was slower than when the sputtering per nA of beam current was higher. The effect of silicon could be direct, but might also be an artifact caused by the suppression of chemical etching when silicon is present or a reflection of different electronic properties of the carbon matrix (e.g., more or sp^2^ versus sp^3^ bonds (conductor versus insulator) when silicon is present.

Thus far, the second largest contaminant observed in Genesis DLC is the SW H. Unlike silicon, DLC with hydrogen can show chemically enhanced sputtering, since the by‐products of H with O and C tend to be gasses or organic molecules that react with oxygen. Hydrogen at SW levels will also likely not directly affect IMF (1) incorporating hydrogen into the structure does not appear to effect the electron mobility in DLC and (2) electrical conductivity for both hydrogenated and non‐hydrogenated DLC is in the same range (Grill 1999). Besides, hydrogen is present in the vacuum system of the SIMS, so SW H overprints a pre‐existing background.

Low‐energy H implants (and other shallow ion implants) have been shown to physically change the structure of DLC. Low‐energy ion implants can break down the sp^3^ structure, producing more sp^2^ bonding especially in proximity to the surface (e.g., Nakazawa et al. 2010); however, others have observed that ion implants destabilize (stress‐relieve) the DLC matrix to produce nanodiamonds (Logothetidis et al. 1998). Implanted SW H may increase conductivity through graphitization or make the sample more insulating by creating nanodiamonds, depending both on the energy of the H ion and the local structure of the DLC.

The amount and distribution of the sp^3^ carbon relative to sp^2^ carbon in the DLC vary spatially, in part as a function of the internal stress state (Friedmann et al. 1994) whether or not H is present. Spatial variability can be inferred from both localized changes of electrical conductivity (Sullivan et al. 1998; Grill 1999) and Raman spectra of thin DLC films (e.g., Friedmann et al. 1994; Chu and Li 2006). In the IMF standard made from flight‐spare DLC, nanometer‐sized up to a &frac12; μm diameter diamond crystals were observed during an SEM session characterizing analysis pits in the calibrated implant (see Table A3). These crystals were observed directly in two of the three pits from the high‐sputter‐rate group; however, the geometry of the surface contamination gives strong circumstantial evidence that, in all three of the analyses with chemically enhanced sputtering, some ions from the primary beam were scattered by diamond crystallites present in the DLC.

The obvious experiment for defining the effect of SW H on the structure and chemistry of the DLC (as well as the SIMS matrix effects) is to implant hydrogen at SW energies and concentrations into DLC and observe the changes, if any. Unfortunately, accurately duplicating SW H on the scale of a wafer is not yet feasible. Plasma ion implantation can duplicate the H concentration at SW depths, but the H depth distribution is complicated and not directly controlled (see Vajo et al. 1994). Standard commercial facilities generally cannot deal with the implantation energies needed to emulate SW (1–3 kV) and the resultant extremely low H‐currents. The exceptions are extremely expensive. The one and only quote for a 1e16 H‐atoms per cm^2^ monoenergetic implant at an energy near the SW average of 2 keV (to be done on a special commercial tool for low implant energies at their minimum wafer diameter of 4″) was $125,000 because of the time needed for the low current implant. That said, although a direct simulation of SW H (necessary for duplicating structural effects) is not feasible, higher energy H implants for testing the chemical effect of H on secondary ion yields are available. Such tests have been performed in house and elsewhere (e.g., Koeman‐Shields et al. 2017, 2018). Our in‐house comparison of DLC analyses with and without commercially implanted hydrogen showed no measurable effect of H on ion yields, within the matrix‐effect induced scatter. So, it is feasible to map a variable IMF to DLC of an appropriate matrix structure regardless of the presence of SW‐level hydrogen.

Finally, note that in addition to spatial variations, the sp^3^/sp^2^ ratio also varies with depth in the thick (1 μm) Genesis DLC films. The reason is that the flight wafers were fabricated by depositing individual 100 nm layers and annealing each one; therefore, the outer surface of the DLC experienced only one annealing while the bottom layers may have experienced ~ 8 to 10 annealing steps. Each annealing tends to grow sp^2^ bonds at the expense of sp^3^ bonds present in the DLC and slightly coarsens the structure (Sullivan et al. 1998). This inhomogeneity with depth *must* be mentioned because the Mg^+^ secondary ion peak is ~100 nm in the calibrated implant, but only about 12 nm in the SW; however, it does not seem to preclude mapping the variable IMF measured in the DLC standard to an appropriate matrix structure in the SW analyses, as evidenced by in‐house work‐in‐progress on SW Mg fluences (preliminary data in Jurewicz et al. 2019).

So, spatial variation in properties of the DLC is rampant and, at this point, not predictable prior to analysis. Jurewicz et al. (2017a, 2017b) were able to map the structure of the DLC using the variations in the SIMS results. For this work, we do the opposite. That is, the data reduction techniques incorporate parameterizations to map DLC structure (after Jurewicz et al. 2017a, 2017b) to track mass interferences and other matrix‐effect‐induced variations in the analyses, as well as to match analyses with an appropriate matrix composition to obtain an appropriate IMF.

#### SIMS Data Reduction

The data reduction method for DLC‐derived SIMS data is based on the typical three‐step process for quantifying SIMS depth profiles. Specifically, that basic process is (1) subtracting background (i.e., the steady signal consisting of the contamination intrinsic to the collector as well as tails of otherwise mass‐separated peaks), followed by (2) correcting the initial data (from near the surface of the collector) for terrestrial surface contamination mixed into the collector during sputtering as well as for transient sputtering effects. Then, finally (3) the profile is integrated and the counts are quantified by comparison with the integrated counts from a standard. However, when analyzing DLC, this simple process requires modifications for matrix effects.

The data reduction procedures for both the SW depth profiles and the standard analyses are outlined below. Although the techniques are optimized for this particular data set, the fundamental concepts can be used for all DLC analyses. Additional technical details are given in the SOM (see supporting information Tables A2 and A3).

#### SW Data Reduction

**Figure 4:**
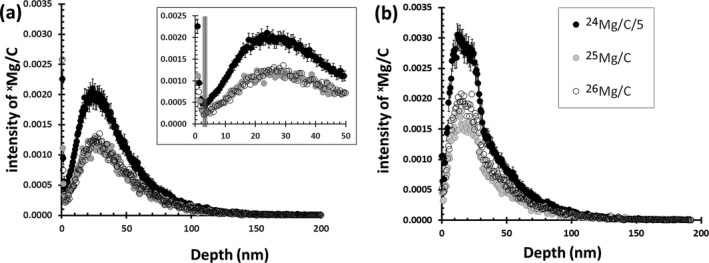
(a, b) SW depth profiles in raw counts; that is, SW_2 in (a) and SW_12 in (b). The depth scale is calculated assuming a constant sputtering rate during the analysis. The profiles in (a) are typical. The inset illustrates that each isotope of Mg is implanted to a slightly different depth (e.g., ^24^Mg peaks at ~24 nm; ^26^Mg at ~29 nm). The composite vertical line marks the local minimum for the three isotopes: before screening total counts from these data for interferences (as in Fig. 5), all data closer to the surface than the local minimum were dropped (“truncated”) to mitigate the influence of ion‐mixed surface contamination. In (b), the ^24^Mg depth profile has an angular peak (~15 to ~30 nm) which drops precipitous at about 35 nm. These are the only two clues that an embedded particulate is responsible for one‐third of the total Mg counts (see supporting Table A2.A for details on SW_12). Profile details: ^24^Mg intensity is divided by 5 to make comparisons easier and the 1ơ error is changed proportionately; error bars not included for ^25^Mg and ^26^Mg for clarity; marker definitions in the legend.

The ^24^Mg, ^25^Mg, and ^26^Mg depth profiles from two SW analyses, one useful for isotopes and one not useful, are given in Figs. 4a and 4b. In *both* cases, all of the profiles start with a relatively high number of Mg^+^ counts that originate from surface contamination; the contribution from the surface decreases sharply with each SIMS duty cycle until, at some point, the Mg^+^ signal reaches a local minimum (e.g., vertical line, inset Fig. 4a). After the minimum signal, the Mg^+^ signal increases, peaks between 21 and 29 nm (depending on the local density of the DLC analyzed) and then decreases rapidly. Eventually, each depth profile exhibits an extremely long, nearly level tail consisting of the highest energy SW ions. What is not evident from a casual inspection is that, although both of the SW analyses in Fig. 4 contain ion‐mixed surface contamination, the profiles in Fig. 4a contain ~6% terrestrial material while the profiles in Fig. 4b contain ~36%. Specifically, the analysis in Fig. 4b samples a near‐surface Mg‐bearing particulate that contributes over one‐third of the counts in all three depth profiles. Although the peak counts in Fig. 4b are high, only the shape itself is definitive: Fig. 3 showed previously that the relative (^24^Mg/C) ion yield in DLC can vary by a factor of 5× due to matrix effects.

**Figure 5:**
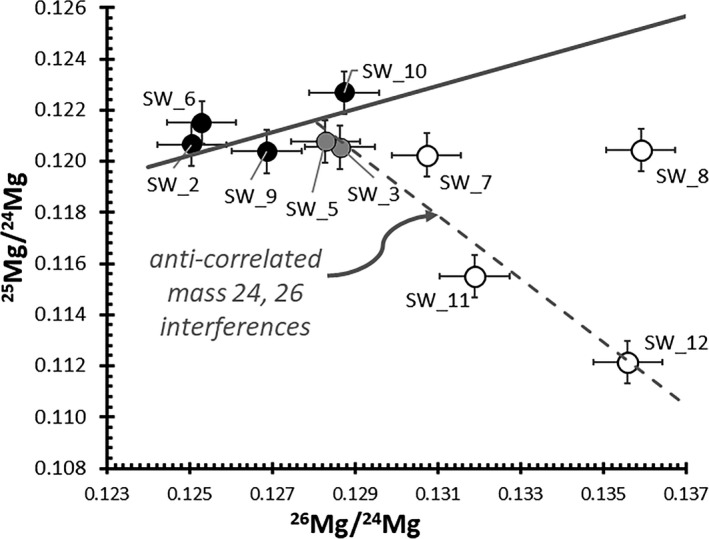
A three isotope diagram for SW data. Ratios use raw total counts, but to mitigate surface contamination the counts within ~4 nm of the surface (i.e., between the local minimum of the profile and the surface—see Fig. 4 vertical line in inset) of the depth profile were dropped). Background and remaining surface contamination are second‐order effects relative to the general trends. Solid line is the terrestrial fractionation line (TFL) with slope (1:2) based on the DSM‐3 composition. All solid markers are analyses distributed along the TFL by the variable IMF. Gray solid markers may be on the trend with the open markers, which are analyses that contain significant, paired interferences (^25^Mg/^24^Mg decreases as ^26^Mg/^24^Mg increases) due to compositional variations in the matrix and that were not used to determine the SW composition (see Table A2 for discussion).

The analysis in Fig. 4b is an extreme example of particulate contamination. It is also an analysis that contains interferences requiring more than the MRP of 1604 M/∆M used to separate them: notice that the ^26^Mg/C intensity is significantly more intense than the ^25^Mg/C intensity in Fig. 4b but overlaps in Fig. 4a. A method had to be developed to remove analyses containing more moderate (and, therefore, harder to observe) particulate contamination as well as unresolved mass interferences effectively. To that end, the first step in the SW data reduction was to truncate the counts that were obviously surface contamination (counts to the surface side of the profile minima). Then using Fig. 5 (in Results), analyses more than 2ơ (~12‰) from the terrestrial fractionation line were dropped. This data screening procedure eliminated analyses containing large particulates and/or extreme interferences.

**Figure 6:**
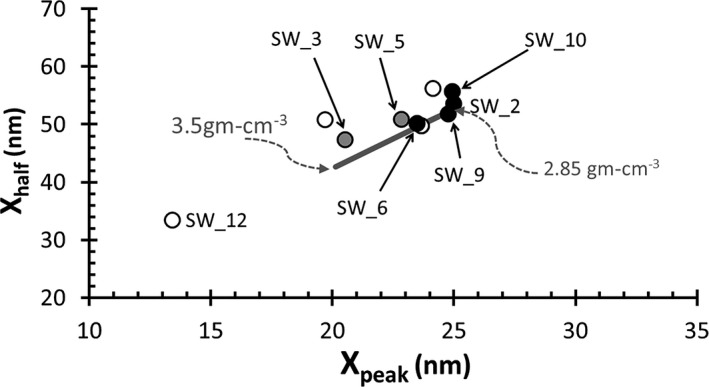
Measured SW ^24^Mg versus SRIM‐calculated depth profiles. Profile shapes are quantified to enable comparisons. Two predetermined points from each depth profile are plotted: *X*_peak_ is the depth at which the SW profile peaks and *X*_half_ is the depth (post peak) at which the SW drops to half of the peak concentration. Markers represent measured depth profiles (fill is as in Fig. 5); the line represents (*X*_peak_, *X*_half_) for all SRIM profiles for DLC calculated using densities from 3.5 to 2.85 g cc^−1^ as indicated. If a marker touches the line, it has a near ideal shape for a DLC density (found by linear extrapolation from the endpoints). SW_8 (not labeled) lies on the line but, in Fig. 5, it appeared to have an interference at mass 26 only; other open markers deviate significantly from the line. SW_3, SW_5, and SW_10 follow the TFL in Fig. 5, but do not have the theoretical shape; however, as these are raw ^24^Mg data, significant background or surface contamination may shift the SW data from ideal, as can mass interferences.

**Figure 7:**
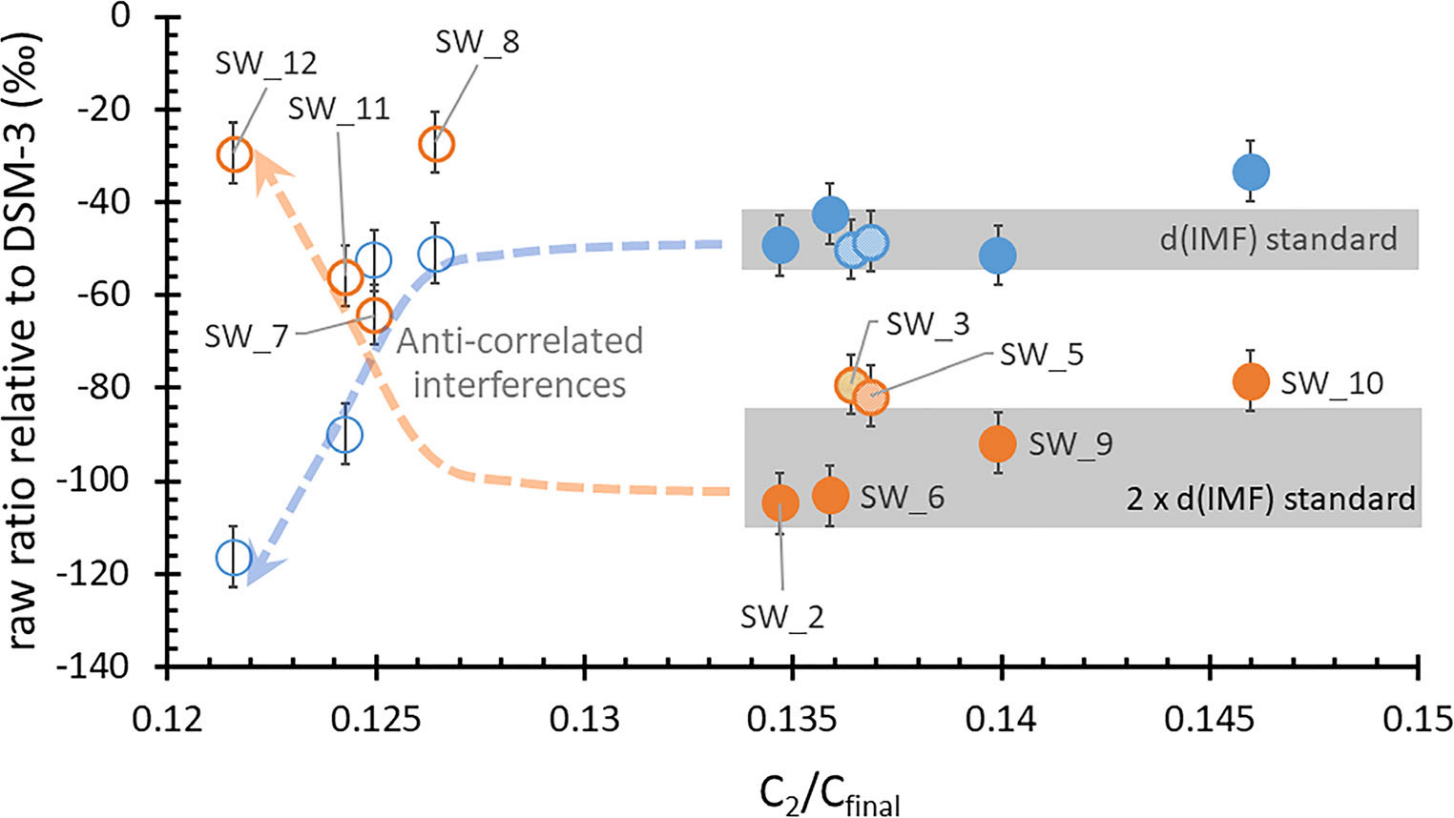
^25^Mg/^24^Mg and ^26^Mg/^24^Mg from Fig. 5 as ‰ relative to DSM‐3 plotted as a function of ^12^C_2_^+^/^12^C^+^_final_, which varies with matrix properties. For each pair, the ^26^Mg/^24^Mg (orange markers) is labeled and the corresponding ^25^Mg/^24^Mg (blue markers) plots directly above, at the same ^12^C_2_^+^/^12^C^+^_final_. Open markers were analyses rejected for interferences in Fig. 5; dashed arrows are the estimated trend of the “anticorrelated” interferences. Gray bars are the range of IMF calculated in the standard; therefore, it is unlikely that scatter greater than the width of the bars can be attributed to IMF. Paired markers will be more negative after the surface correction if interferences are not present. (Color figure can be viewed at http://www.wileyonlinelibrary.com.)

Next, two parameterizations were used to assess the quality of the remaining data. One compared the shape of the analytical profiles with the shape of the SW implant profiles calculated using SRIM. The other looked at the internal consistency of the isotopic values calculated from the (truncated but otherwise raw) data versus matrix‐based parameters. Plots of these two parameterizations are given in the Results section (i.e., Figs. 6 and 7 in Results) as part of the quality assessment.

Finally, the data were corrected for background and surface contamination. Since background was not directly measurable in the SW analyses because of the long tail on the SW implants, the SW data correction required an iterative procedure by which the depth profiles of all three isotopes were fit by SRIM models by minimizing the χ^2^ (“chi‐squared”) variance. This procedure was based on three assumptions. The first assumption was that the density of the DLC was the same for all depth profiles of a single analysis. The second assumption was that the implanted SW ions had not moved after collection (i.e., there was no radiation‐induced segregation). The third assumption was that SRIM gives a realistic SW distribution: no SIMS artifacts (i.e., ion mixing and/or beam drift did not change the overall shape of the implant and was accommodated in the calculated density) and negligible errors in the Monte Carlo calculation of the SRIM curve, the SW velocities, and/or the matrix properties chosen for the DLC. The assumption that the SW ions have not moved since implantation would not be appropriate for some materials (e.g., silicon), but is fine for the type of DLC used by Genesis, anhydrous, tetrahedrally coordinated DLC (see Vainonen et al. 1997).

To minimize the χ^2^, DLC density, SW intensity, and background were varied independently to fit the ^24^Mg depth profile; only intensity and background were varied for the ^25^Mg‐ and ^26^Mg‐ depth profiles since the density was assumed to be the same for the three isotopes. Although the background was ≪1cps, for these isotope calculations, it had a significant effect on the SRIM fits. Moreover, probably because of either interferences or SIMS artifacts (like beam drift), the χ^2^ fits of a few SW depth profiles showed more than one local minima (i.e., when different initial fit parameters were chosen, the iterated best fit was different). When more than one minimum χ^2^ was observed, the combination giving the smallest residuals over the entire profile was used. Details of this iterative data reduction technique are presented in Table A3 for SRIM fits and supporting information Table A5 for residuals.

**Figure 8:**
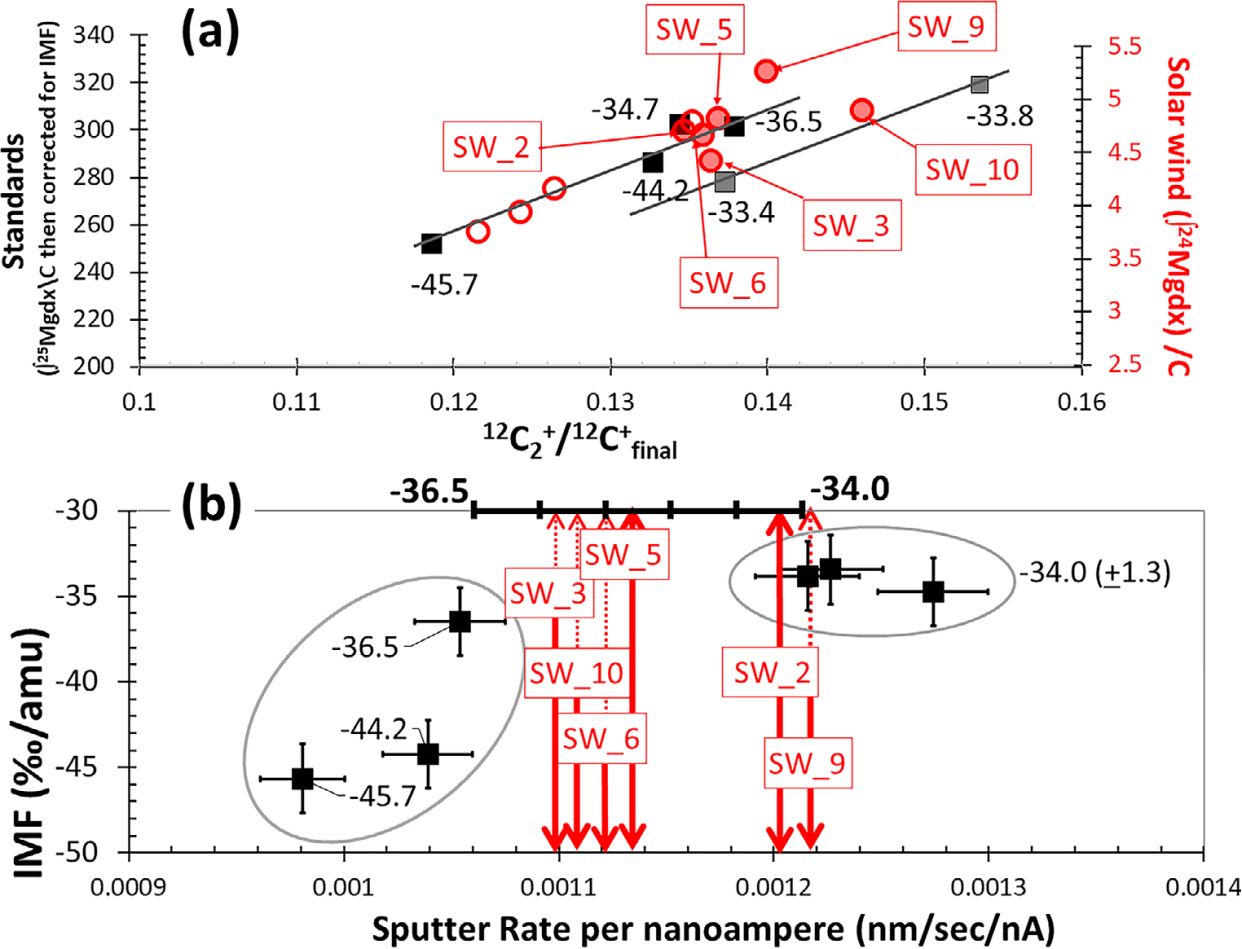
a, b. Two methods for mapping the IMF of SW analyses to matrix‐appropriate standard analyses. Square markers are data from standard analyses (matrix‐normalize integral of ^25^Mg^+^ corrected for IMF as labeled). In (a), the standard data from two trends controlled by the dominant matrix effect: one (black markers and line) reflects variable, minor Si and the second (gray markers and line) reflects dominant sp^3^ bonds (diamond and/or nanodiamond = high electrical resistivity) and density. To find the matrix appropriate standard for the SW in (a) the matrix‐normalized integral of ^24^Mg^+^ yield in SW analyses (open circles have interferences; six filled circles are SW near TFL) has been matched to the trends in the standard. The matrix‐appropriate IMF for the SW is extrapolated from the closest standards. Note that all six of the filled SW markers plot such that their IMFs are between −33.4‰ and −36.5‰ (per amu). In (b), the oval on the left contains the standard analyses of the low sputter rate, high ion yield cluster while the oval on the right contains the analyses of the high sputter rate, low ion yield cluster (see inset Fig. 3; detail in Jurewicz et al. 2017a, 2017b). Labels are IMF, and the axis at the top is the interpolation of IMF between clusters. To find the IMF, SW data are plotted by S/nA and the matrix‐appropriate IMF is estimated from the upper axis (vertical arrows are SW analyses represented by the filled circles in [a]). Note: the methods in (a) and (b) give similar, but not identical, results, which is why a systematic error is added to all of the final SW data. (Color figure can be viewed at http://www.wileyonlinelibrary.com.)

After the SRIM fit, the final calculation for the SW isotopes was as follows. The background‐corrected profile for each isotope was integrated, with the first 4 nm and the tail calculated using the SRIM fit. Contamination was then subtracted (calculated from the measured profile—SRIM profile) for ^24^Mg and assuming terrestrial composition (corrected for IMF) for ^25^Mg and ^26^Mg. Note: IMF was determined using parameterization to match appropriate matrices (see Fig. 8 in Results). Isotopic ratios were calculated from the net integrated counts, corrected for IMF, and 3‰ (for the ^24^MgH interference) was subtracted from the d^25^Mg.

#### Calibrated Implant (IMF Standard) Data Reduction

Quantifying the IMF values from the calibrated implant required only minor modifications to the technique used for homogeneous materials. Standard analyses were run overnight, so the background was precise and was subtracted directly. Because the implants were minor ions at high doses, any particulates or other irregularities pre‐existing in the DoS were generally too small to make a significant contribution to the total counts. Also, because the standard was a two‐isotope implant, the third isotope (^24^Mg) was used to trace sources of contamination—both surficial and embedded.

The measured ^24^Mg was not just background in the DLC. A small dose of ^24^Mg (less than the SW ^24^Mg fluence) was implanted with the high doses of ^25^Mg and ^26^Mg, probably as ^24^MgH. Such “accidental implants” are not unusual, as the MRP of tools used for ion implantation is generally poor; for example, tails of large adjacent mass peaks or molecular interferences are often implanted at doses that are %‐level relative to that of the intended ion implant. But, the presence of the accidental implant meant that not all of the measured ^24^Mg^+^ was terrestrial. So, for a precise correction to the measured counts of ^25^Mg and ^26^Mg, the non‐terrestrial component had to be removed from the measured ^24^Mg. To do this, the accidentally implanted ^24^Mg was modeled using SRIM (details in Table A3). Then, the model counts were subtracted from the measured counts. The remaining counts of measured ^24^Mg^+^ were terrestrial (from the surface contamination), so proportional amounts of ^26^Mg and ^25^Mg were subtracted from the SIMS data. In theory, this small correction for the ion‐mixed surface contamination should have been corrected for IMF; however, this small correction (0.0–0.4‰) was negligible relative to the total propagated error.

For quantification, the ^25^Mg and ^26^Mg profiles were integrated separately and then divided to give the measured ^26^Mg/^25^Mg ratio for each analysis. This ratio varied with position even though the implanted ^26^Mg/^25^Mg ratio determined by ICPMS was uniform (see Fig. 3). The difference between the SIMS and ICPMS ratio for each analysis was the local IMF.

## Results

Figure 5 gives the ^25^Mg/^24^Mg versus ^26^Mg/^24^Mg results for the raw SW data. To mitigate the effect of surface contamination on the raw data, the counts from the surface to the minimum of the depth profiles were deleted (see vertical line in Fig. 4a inset). Background was not subtracted for this plot; however, as later SRIM modeling showed, this was not the cause of the primary trends in Fig. 5. That is, the solid line and the dashed lines, which are the terrestrial mass fractionation line (TFL) and a line of anticorrelated interferences, the best fit (*R* ^2^ = 0.9) to SW 3, 5, 7, 11, and 12. That is not intended to suggest that the anticorrelated interferences are the only interferences present in the Fig. 5 data. Backgrounds have not been removed and, for example, Table 2 (background corrections for the standards) shows correlated interferences at mass 25 and 26 (STD_2 and STD_4) that may be (^12^C_3_N)^++^ and (^12^C_3_O)^++^. These interferences do not, however, drive the dominant trends of Fig. 5.

The black and gray markers in Fig. 5 are scattered along the solid line which, here, reflects the variable mass‐dependent fractionation within the SIMS. Indeed, most of the data fall within statistical error (2ơ = ∼12‰) of the TFL. The open markers are mostly distributed along the dashed line, where the ^26^Mg/^24^Mg increases, but there is also a corresponding decrease in the ^25^Mg/^24^Mg ratio. This second trend is likely the result of interferences due to the presence of silicon: SW_12 is the analysis in Fig. 4b containing the large particulate. A particulate of dust should contain some excess ^24^Mg‐H counts but, since the ^25^Mg/^24^Mg ratio decreases, changes in ^24^Mg‐H content is clearly small relative to the other factors. The most likely candidates for the dominant interferences, and supporting Raman Spectroscopy results, are deduced and discussed in Table A2.

A corollary to the evidence in the SOM: for the likely interferences at mass 26, which are (^28^Si^12^C_2_)^++^ and (^28^Si^24^Mg)^+2^, an MRP of 4427 M/ΔM would resolve the silicon carbide interference, but the magnesium silicide interference cannot be resolved and would need to be parameterized given any analytical conditions. Moreover, in the presence of silicon, the relative yield of ^24^Mg^+^ may decrease by many percent due to the presence of Mg‐rich silicon hydroxides when the amount of ^24^MgH^+^ increases, again requiring parameterization to quantify. Note that this parameterization of silicon interferences and ion yield is done implicitly during the calibration of measurements of Mg in H‐implanted silicon wafers.

Figure 6 compares the shape of the SW ^24^Mg depth profile from each analysis to models of SW implants calculated using SRIM by plotting two points for each profile: the depth of the peak of each depth profile (*X*_peak_) on the *X*‐axis, and the depth at which the SW counts drop to half of the peak signal on the high‐energy side of the depth profile (*X*_half_) on the *y*‐axis. The uniformity of the DLC within a given profile (and between profiles) is also tested in Fig. 6 because the profile's shape depends on the structure and composition of the matrix. Because the depth for each SIMS duty cycle in the profile was calculated by assuming a constant sputtering rate, the shape can also depend on the reliability of the assumption of constant sputtering rate. But, since the primary current was reasonably stable, the primary control on the profile shape is likely the density coupled with chemical properties of the matrix. Specifically, silicon decreases the effective stopping power of the matrix and profiles are skewed to longer tails; density and, to a lesser extent, sp^3^ bonding increase the effective stopping power of the carbon matrix.

Open markers are the excluded SW data, as per Fig. 5. Labeled markers are SW_2, 6, 9 which fall on the SRIM line as well as SW_3 and 5 (gray markers) and SW_10 (labeled black marker) that fall just above the SRIM line. These six analyses are all within 2ơ error of the terrestrial fractionation line in Fig. 5. The analysis SW_12 is labeled because it was reviewed earlier in Fig. 4b. SW_11 is the only profile previously identified as containing interferences consistent with the SRIM line but, in Fig. 5, appears to have a mass interference at mass 26 only. On Fig. 5, SW_11 plots very close to SW_12, which samples an Mg‐bearing particulate and also exhibits a very distorted shape. It is possible that SW_11 reflects silicon from the sputtering target used to make the DLC (e.g., Friedmann et al. 1994) rather than embedded dust or other contamination during fabrication (Si‐C bonds were seen in Raman adjacent the SW_11 pit—described in the Table A2).

In any case, SW_11 illustrates that the shape factors give interesting and useful information, but that there are multiple scenarios that define the same shape. That is, although the shape is consistent with the theoretical model, there is a chance that there may be an issue with the analysis. Conversely, an *X*_half_ longer than ideal may also represent and artifact of “large” amounts of ion‐milled surface contamination, which can be corrected using SRIM or even a process SRIM does not consider, such as channeling through oriented diamond crystallites.

To better understand the information contained in Figs. 5 and 6, Fig. 7 gives the ratio for each analysis from Fig. 5 as an isotopic shift in per mil relative to DSM‐3 plotted versus a matrix parameterization (^12^C_2_^+^/^12^C^+^_final_) to sort the data by matrix properties. Since the ^12^C_2_^+^/^12^C^+^_final_ for the SW analysis is steady‐state data, it is the equivalent to the ^12^C_2_^+^/^12^C^+^_average_ parameterization for analyses of the IMF standard. Jurewicz et al. (2017a, 2017b) showed that these parameters roughly trend with the matrix silicon content and sp^3^/sp^2^ bonding.

In Fig. 7, the anticorrelated interferences are clearly associated with the lower values of ^12^C_2_^+^/^12^C^+^_final_; that is, elevated concentrations of silicon, consistent with the likely interferences deduced in supporting information A2. The two dashed arrows estimate the trends of these interferences. As in Fig. 5, SW_8 shows no obvious interference at mass 25, but is very high at mass 26; yet Fig. 7 suggests that SW_8 is in the trend of anticorrelated interferences. The gray bars give the range of IMF variation seen in the standard (e.g., largest IMF minus smallest IMF). The thin gray bar (d[IMF]/amu) can be forced to cover all but one of the points (SW_10). The thicker gray line can cover SW_9 and either SW_2, SW_6 (which, with SW_9, plotted on the line of ideal SRIM curves in Fig. 6) or SW_3, SW_5, and SW_10, which had longer tails than the ideal SRIM curves of Fig. 6. However, removing terrestrial material from an otherwise interference‐free SW profile will shift both the d^25^Mg and d^26^Mg to more negative values; removing the 3‰ ^24^Mg‐H mass 25 interference (see Fig. 2) will further make d^25^Mg more negative; and removing a uniform background could move these isotopic shifts either way. So, although these SW analyses are not disqualified, Fig. 7 clearly defines potential issues and can serve as a point of discussion for the results after the background and surface corrections.

All analyses whose raw data plotted parallel to the terrestrial fractionation line in Fig. 5 were first corrected for background, integrated and corrected for surface contamination using the iterative technique outlined previously in the SW Data Reduction and detailed in Table A3. The initial SW counts (from <4 nm) and the tail SW counts (starting from whatever depth background was encountered) were added using SRIM. Isotopic compositions were calculated using the ratio of the integrals, correcting for IMF, and then d^25^Mg was corrected for 3‰ ^24^Mg‐H. The correction for IMF was performed though parameterization, as given in Fig. 8.

An important aside to the calculation of the SW isotopes: two parameterizations are used to match SW data with matrix‐appropriate standard values. Specifically, Fig. 8a uses the carbon normalized integrated ion yields. To create Fig. 8a, the ^12^C_2_^+^/^12^C^+^_final_ of plots for the standard and SW integrals were superposed, then the plot of SW data was stretched and translated (in intensity only) to best match the trends in the standard. Note that the plot of the carbon‐normalized ^25^Mg integral in the standard is corrected to represent a ^24^Mg count rate by the measured IMF. All six of the data near the terrestrial fractionation line in Fig. 5 have relatively high ^12^C_2_^+^/^12^C^+^_average_ values, interpreted as DLC matrices low in silicon transitioning to chemically enhanced sputtering at the higher ^12^C_2_^+^/^12^C^+^_average_ values. Figure 8b plots current‐normalized sputtering rates for SW and standard depth profiles as a second estimate of IMF (see the inset in Fig. 3, which gave S/nA versus relative ion yield, suggesting that S/nA was also a function of silicon concentration). So, although the total variation in IMF across the standard was ~12‰ per amu, Figs. 8a and 8b indicate that the total range of IMF for all six analyses to ~3 to 4‰ per amu and that the IMF for each analysis should be accurate to ±1.5‰ per amu.

**Figure 9:**
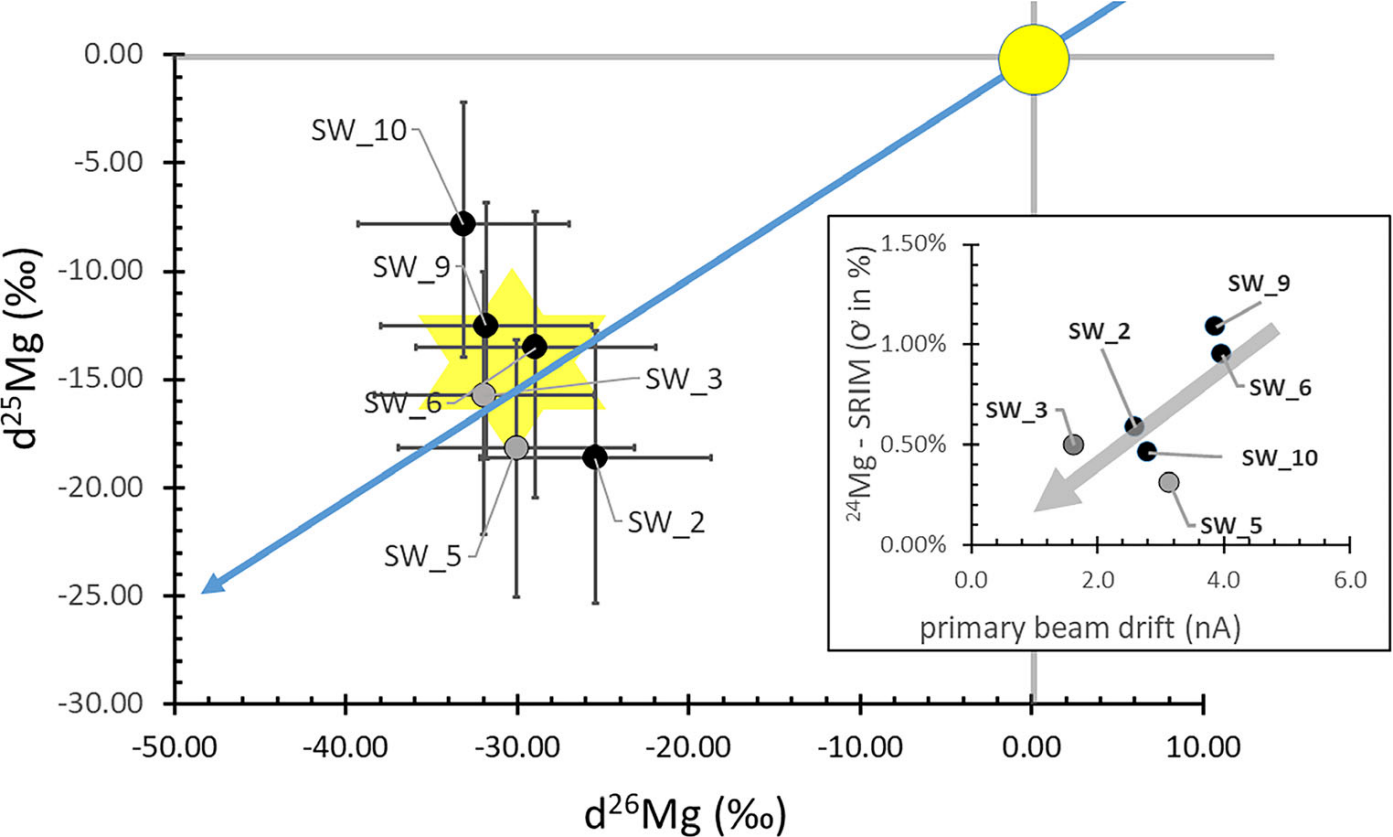
Final isotopic fractionation calculations for the six analyses within 2ơ of the terrestrial fractionation line in Fig. 5. Error bars are 1ơ of the SRIM fit, about the same as counting statistic error—both of which are controlled by the low counts for the minor ions. Yellow circle is terrestrial; yellow star is SW (breadth ~2ơ SE of all six analyses); solid blue line is the terrestrial fractionation line. The inset suggests that the small error in the SRIM fit is at least in part due to primary beam drift during the analysis.

The final results, as well as the final fit parameters, are given in Table 3. Figure 9 plots the results with the propagated statistical error to the SRIM fits. The inset of Fig. 9 shows that the propagated error seems to be related to drift in the nominal primary current during the analysis, which was not correctable using the software in use when these data were collected. The resultant average SW fractionation, plotted as a star on Fig. 9, is (−14.4‰, −30.2‰). Error propagation (1ơ) from the SRIM fit calculation in each analysis plus ~(1.5‰ per amu) error in IMF averages (5.7 ± 0.2‰, 6.6 ±0.1‰). The error propagation is detailed in Table 4. This error is approximately the 1ơ propagated counting statistics you would get by only considering the number of counts of ^24^Mg, ^25^Mg, and ^26^Mg obtained in each depth profile, taking the square root of the counts, and propagating the errors. The 2ơ standard error (SE) for the six SW values is (3.3‰, 2.3‰) and the largest difference in the IMFs calculated from Fig. 8a versus 8b is 2‰ per amu, giving a ±1.0‰ error on IMF. Adding another ±0.5‰ to the IMF error (since the scales were manually drafted) and then the adding the conservative IMF error of (2.5‰, 5‰) to the 2ơ SE using quadrature gives the error used in the discussion: (4.1‰, 5.5‰).

**Table 3:** Parameters and results for SRIM‐fits to SW depth profiles used in surface and background corrections

Solar wind analysis	d^25^Mg^a^	d^26^Mg	Density	IMF	Background			∆nA	SRIM fit to data (%)			Range of ^24^Mg fit (nm)	Range of ^25^Mg, ^26^Mg fits (nm)
					^24^Mg	^25^Mg	^26^Mg		^24^Mg	^25^Mg	^26^Mg		
SW_2	−18.64	−25.46	2.98	−34	0.400	0.048	0.050	2.60	0.59	1.89	1.72	350–1300	350–1300
SW_3	−15.73	−31.96	3.15	−34	0.265	0.030	0.068	1.62	0.50	1.53	1.26	300–1400	300–1200
SW_5	−18.17	−30.08	2.94	−35	0.503	0.068	0.054	3.13	0.31	1.30	1.33	300–1400	400–1200
SW_6	−13.51	−28.96	3.05	−35.5	0.1	0.03	0.03	3.97	0.95	1.80	2.13	300–1300	300–1000
SW_9	−12.50	−31.84	2.89	−34	0.315	0.030	0.047	3.88	1.09	1.50	1.37	500–1500	500–1500
SW_10	−7.84	−33.16	2.85	−32	0.000	0.023	0.090	2.80	0.46	1.07	< 0.01	500–1500	500–1500
SW_8^b^	−3.52	76.71	2.80	−44.8	0.103	0.015	0.025	3.30	1.21	2.41	3.08	300–1400	450–1000
SW_11^b^	−35.82	81.38	3.11	−45.5	0.396	0.038	0.096	4.51	0.49	1.09	1.58	300–1400	350–1200

a Includes 3‰ correction for ^24^MgH.

b Contain interferences (see Figs. 5 and 7). Gray shading designates that these analyses were not used for calculating SW Mg isotopes.

**Table 4:** Error propagation for individual analyses based on SRIM fit

	2ơ (‰) ^24^Mg dirt (d^25^Mg and d^26^Mg)	2ơ (‰) d^25^Mg (SRIM calc.)	2ơ (‰) d^25^Mg (IMF err.)	1ơ (‰) d^25^Mg	2ơ (‰) d^26^Mg (SRIM calc.)	2ơ (‰) d^26^Mg (IMF err.)	1ơ (‰) d^26^Mg
SW_2	0.5	11.7	2.5	5.88	13.1	5	6.72
SW_3	1.2	11.2	2.5	5.69	12.4	5	6.41
SW_5	0.7	9.9	2.5	5.02	13.4	5	6.89
SW_6	0.8	12.4	2.5	6.26	13.6	5	6.99
SW_9	0.7	11.2	2.5	5.65	11.9	5	6.15
SW_10	0.8	11.2	2.5	5.65	11.9	5	6.16
Ave error				5.8			6.9
SD				0.2			0.1

## Discussion

### The Effect of Systematic Errors and Related Assumptions on the Accuracy and Interpretation of the SW Results

The primary assumption used when evaluating the data was that the data having negligible interferences in Fig. 5 had to lie “near” the terrestrial fractionation line in Fig. 5 because of the variable IMF (a mass‐dependent fractionation within the SIMS). The 1ơ propagated statistical error for the SW counts in each analysis was ~6‰. So, the four SW analyses thrown out using Fig. 5 were all greater than ±12‰ from the terrestrial fractionation line. Moreover, Fig. 5 shows that none of the six remaining points were exactly on the TFL. So, any findings of non–mass‐dependent fractionation of Mg isotopes in the SW (or even excess ^26^Mg from the decay of ^26^Al) were not excluded by the assumption that the data were near the TFL—assuming that any such effect is less than ±12‰.

The second major assumption is that SRIM accurately calculates the distribution of the SW in the complex DLC matrix. That is, SRIM not only estimates where ions will stop during collection, but using SRIM for data reduction assumes that the initial distribution of the captured ions does not change with time. Note that SW in Genesis silicon exhibits radiation‐induced segregation (e.g., King et al. 2008); however, the errors for the SRIM‐fits in Table 4 strongly indicate that this segregation process (caused by diffusion of vacancies created by the ion implantation) is significant in DLC at SW collection temperatures. A lack of mobility of implanted ions in DLC was reported in a solid‐state physics of materials study by Vainonen et al. (1997) for a similar DLC‐type. Another indicator supporting the use of SRIM for the SW ion distribution is given in Table A3. There, the shape of “contamination” removed from two SW depth profiles using SRIM is modeled by changing the intensity of contamination removed from STD_7, the least‐contaminated IMF standard analysis. If significant diffusion had occurred in the SW sample, the contamination indicated by SRIM would not have the shape of ion‐mixed contamination from the standard. Even if some diffusion had occurred in both, the distribution and concentrations of defects that would have controlled that diffusion would be significantly different in the SW and the implant. A caveat: in the few areas in DLC with “high” silicon content (e.g., areas containing particles, such as SW_12 in Fig. 4b) radiation‐induced segregation may (or may not) occur; however, isotope measurements from silicon‐rich areas were excluded by our screening process (Figs. 5, 6, 7). The clustering of the final set of SW data in Figs. 8a and 8b also suggests a narrow range of matrix composition, low in silicon.

For SRIM to accurately model the SW, it is also implicit that the energy spectrum of SW Mg ions has a negligible error. The energy spectrum of SW ions was gleaned from velocity data recorded by spacecraft. There are some very high (and, possibly, some very low) velocities which are certainly missed due to the limitations of the spacecraft instruments (e.g., safe mode during coronal mass ejections); but, since the reprocessing of the ACE data (Pilleri et al. 2015 and references therein), our SRIM fits using the bulk SW velocity distribution look very good, indeed.

Finally, it is important to assess the effect of non‐mass resolved interferences (e.g., ^24^MgH, [^28^SiC_2_]^+2^) on the accuracy and scientific application of these data. First, note that the data reduction technique should have removed all “interferences” due to background counts, including counts due to the uniform distribution of silicon; that is, (^28^SiC_2_)^+2^. In addition, significant, nonuniform mass resolved interferences (e.g., silicon‐carbide rims on embedded particulates) should be discernable as local excursions of measured counts from the SRIM fit. So, interferences remaining in the six SW analyses are likely from other SW ions.

As discussed in Table A3, information on interferences from SW ions can be gleaned by studying the analyses exhibiting anticorrelated interferences (Figs. 5 and 7). For example, although ~30% of the SW_12 Mg counts are from an embedded, Mg‐bearing particulate (Fig. 4b) and, so, hydrides must be present throughout the zone of implanted SW H, the ^25^Mg/^24^Mg ratio decreases. The solution is that (^24^Mg‐H)^+^ is certainly not the only hydride (and/or hydroxide) that forms in a silicon‐rich area of DLC. In fact, the preliminary results for ^24^Mg fluences from DLC (e.g., Jurewicz et al. 2019) show that the bulk Mg fluence appears to decrease ~10% in SW_12 versus SW_2. Clearly, this is a standardization issue: there is enough hydroxide (and/or hydride) formation to measurably lower the net ^24^Mg^+^ ion yield. Other interferences (e.g., [^28^Si^24^Mg]^+2^) are likely present at mass 26 (Table A2). Since the initial SIMS setup for this data set was performed near STD_2 (a low silicon area), the mass resolution was not set to resolve hydrides. So, where interferences exist, there is an entire set of Mg‐H–related interferences and, thus, the ^25^Mg/^24^Mg and ^26^Mg/^24^Mg ratios are anticorrelated.

The corollary to the discussion of interferences above and in the SOM is that if silicon is not present, then ^24^MgH formation is repressed. That is, screening for silicon also screens for significant ^24^MgH formation, but certainly does not preclude a ^24^MgH interference at the ~3‰‐level (Fig. 2). In fact, small variation in the amount of the ^24^MgH interference at the per mil level is probably why the 2ơ SE for the (d^25^Mg, d^26^Mg) without the IMF correction is (3.3‰, 2.3‰) and not (1.2‰, 2.3‰) as would be expected if the percentage of error was approximately the same for both isotopes (see Tables 3 and 4). In Table A5, it is shown that the (^24^MgH)^+^ is even matrix dependent at the ‰ level, but likely averages roughly 3 ‰ per amu. This inference is supported by the fact that the mean of the d^25^Mg in ‰ per amu was only 0.7‰ different (heavier) than the mean of the d^26^Mg. But, because of that variability, d^26^Mg (not d^25^Mg) is used to determine the SW Mg fractionation.

### Solar Nebula versus Solar Photospheric Mg Composition

As reviewed in the Introduction, the ‰‐level uniformity of Mg isotopes in extraterrestrial materials strongly suggests that the Mg‐composition we measure represents a nebular composition. However, the specific goal of this work is to find the amount of Mg fractionation due to the process of SW formation and acceleration away from the Sun, ^Mg^Δ_(sw‐p)._ This ultimate goal raises the issue of whether or not the nebular composition is exactly the photospheric value.

There is some suggestion that light isotopes have settled out of the convective zone of the Sun, producing a ‰‐level elemental and isotopic fractionation (“gravitational settling”) theorized to occur over the past 4.5 Gy in the solar convective zone. Bochsler (2000) estimated the fractionation of Mg isotopes in the convective zone to be 3–4‰ per amu, while Turcotte and Wimmer‐Schweingruber (2002) estimated ~2–3‰ per amu. If true, the measured fractionation from nebular (here DSM‐3) must be corrected for a ~3‰ per amu non‐photospheric (d^25^Mg, d^26^Mg) component of fractionation in the SW sample of (−3‰, −6‰). That would make the (d^25^Mg, d^26^Mg) from our measurements relevant to the models for the formation of the SW (−11.4‰, −24.2‰). However, Laming et al. (2017) and Bochsler (2007) do not include gravitational settling in their models even though its relevance is discussed in the ICD papers, and Bochsler (2000) further discusses the possible range of magnitudes for the effect.

In McKeegan et al. (2011), the effect of gravitational settling was discussed but no correction was made to the SW O data. The goal was to determine the nebular composition, not the solar composition, and CAIs are an excellent proxy for the O‐isotopic composition of the solar nebula. The CAI composition required only a −22‰ per amu correction for SW fractionation versus the −26‰ per amu fractionation of O in the SW predicted by the ICD model. If gravitational settling had made the solar composition lighter than CAI prior to SW formation, the discrepancy between the predictions of ICD model and CAI proxy would be even larger. An estimate of the O settling by Bochsler (2000) was −4.5‰ per amu; Marty et al. (2011) assumed −3‰ per amu for O.

### Models of ^X^Δ_(sw‐p)_—Overview

Theories to explain the compositional changes observed in the SW invoke various plausible forces, including electrical interactions with other streaming ions (e.g., Coulomb drag), forcing by waves (ponderomotive force) at the base of solar magnetic loops and interaction with the expanding solar magnetic field through conservation of the first adiabatic invariant, gravitational settling within those loops, etc. Since the inner workings of the Sun cannot be measured directly, determining which, if any, of these processes dominate the observed mass fractionation requires precision data for solar elemental and isotopic composition to compare with theory. Data having the precision needed to validate these theories are only available (albeit time integrated) through Genesis sample analyses; spacecraft or spectrographic measurements currently include more measurement and/or systematic errors. Therefore, comparison of models with the Genesis results is also an opportunity to test the relevance of solar physics assumptions.

This study compares the measured Mg isotopic fractionation in the SW with predictions by three very different methods. These are (1) the collisional Inefficient Coulomb Drag (ICD) model (after Bodmer and Bochsler 2000; Bochsler 2007), (2) a collisionless wave interaction model (Laming et al. 2017), and (3) a cosmochemical proxy after the ^O^Δ_(sw‐p_) = (SW‐CAI) of McKeegan et al. (2011) adapted for the scaling other elements as used for N by Marty et al. (2011).

The ICD model envisions a cascade of collisions between the minor ions and the (major) H component, with H flowing the fastest, thus accounting for the significant He depletion in the SW and the movement of the minor ions outward. Bodmer and Bochsler (1998, 2000), Bochsler (2007), and related models are based on this process of coulomb drag that is strongly dependent on the flow of protons and the charge states of the minor ions; but, the ability of the minor ions to couple with the proton flow is species dependent. Numerical values of fractionation are based primarily on He and Ne isotopes from both spacecraft and the Apollo foil experiments and ratios are predicted to vary with SW source. Details in Bochsler (2007). However, the ICD model did not explain Genesis ^4^He/^20^Ne results in the slow and fast SW regime samples Heber et al. 2012).

The Mg fractionation based on the ICD theory for this study uses the values: (1) the photospheric He/H ratio is 0.084, (2) the SW He/H ratio is 0.045, and (3) an average Mg charge state is +8.9 for the bulk SW (7.8% Mg^+6^, 6.2% Mg^+7^, 17% Mg^+8^, 28% Mg^+9^, 40.3% Mg^+10^). Note that (1) is from Basu and Antia (2004, 2008), while (2) and (3) are from the GIMs and the recently reprocessed ACE data (e.g., Pilleri et al. 2015). Given these assumptions, the Bodmer and Bochsler (2000) model predicts an isotopic fractionation for Mg in bulk SW of ~10.5‰ per amu; that is, a (d^25^Mg, d^26^Mg) of (−10.5, −21).

The Laming et al. (2017) models envision that fractionation of the SW is driven by the ponderomotive forces produced by electromagnetic magnetohydrodynamic waves at the base of solar magnetic loops followed by conservation of the first adiabatic invariant; that is, the gyroscopic motion of SW ions around magnetic field lines is converted to a velocity linear to the field lines as the magnetic field lines expand. Variation of SW composition with source (i.e., fast, slow) is accommodated by modeling fractionation in both straight (expanding) magnetic field lines and coronal loops.

Laming et al. (2017) constrained their model input parameters to best fit the currently available Genesis elemental and isotopic data. The elemental fractionation is predicted quite well, even in the early versions (e.g., Laming 2015; Laming et al. 2017). The isotopic fractionations of heavier isotopes Ne and Ar are well predicted, the lighter isotopes O, N predictions are slightly low, but the predicted He isotopic fractionation is currently opposite to that observed, likely because ^3^He is subject to extra physics not included in the model (discussion Laming et al. 2017).

The last model for ^X^Δ_(sw‐p)_ is a proxy method based on that used for oxygen isotopes by McKeegan et al. (2011) and adopted by Marty et al. (2011) for N isotopes. When oxygen isotopes were measured in a silicon carbide target of the Genesis Concentrator, McKeegan et al. (2011) had a conundrum. The ^O^Δ_(sw‐p)_ for SW fractionation calculated using the ICD model made the solar oxygen isotopic composition “heavier” than isotopes observed in extraterrestrial samples. The only known materials with O isotopic compositions even close to the ICD‐predicted composition were ultra‐refractory hibonite grains from some carbonaceous chondrites. McKeegan et al. (2011) also knew that the ICD model did not explain Genesis ^4^He/^20^Ne results in the slow and fast SW regime samples. Accordingly, they conservatively concluded that the solar oxygen isotopic composition looked like early solar system condensates (i.e., CAIs).

The ^O^Δ_(sw‐p)_ used by McKeegan et al. (2011) to map the SW O isotopes measured to the CAI composition was lower than that predicted by ICD. Specifically, the ^O^Δ_(sw‐p)_ was lower by a factor (1.019/1.026) = 0.9932. The factor 1.019 is the CAI O value of ~1.022 from McKeegan et al. (2011) which Marty et al. (2011) then corrected to photospheric by adding a correction of 3 ‰ per amu for gravitational settling. This factor was then used to calculate the ^N^Δ_(sw‐p)_ for the solar N isotopes in Marty et al. (2011) and will be used to calculate a ^Mg^Δ_(sw‐p)_ (i.e., CAI proxy) for the solar Mg isotopes for comparison with the solar physics models.

### Scientific Implications

**Figure 10:**
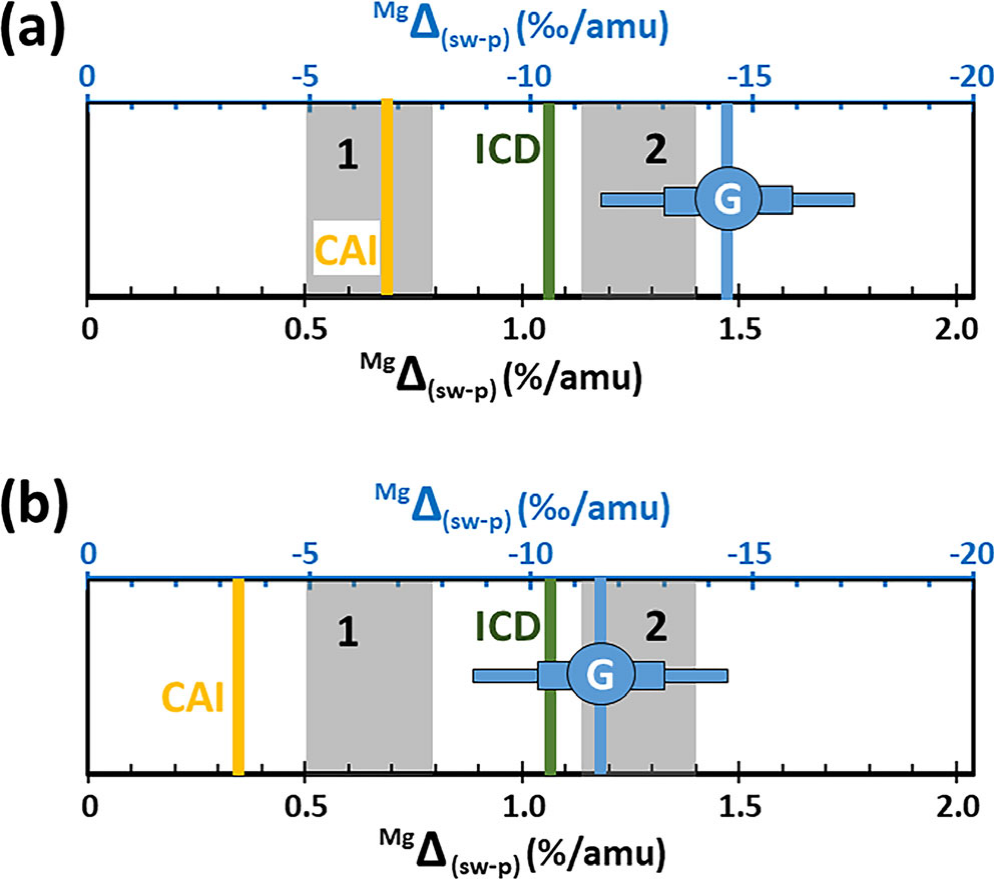
Comparison of predicted SW Mg fractionation with Genesis data: (a) SW as measured (b) SW after correction for gravitational settling (i.e., assuming that photospheric composition is 3‰ per amu different from that of nebular for both these data and CAI proxy). Theoretical models are not adjusted for gravitational settling, which would change their initial conditions. Symbols: vertical blue line (marked G) is data from this study—horizontal blue rectangles cover the 1ơ error (wide) and the 2ơ error (narrow), respectively. Gray boxes are the predictions of Models 1 and 2 by Laming et al. (2017), while the vertical green line and the vertical yellow line are the ICD and CAI proxy models, respectively.

Comparisons of all three models (ICD, both Laming et al. 2017 numerical, and CAI proxy) with the Genesis results (as d^26^Mg per amu) from this study are given in Figs. 10a and 10b. Again, the errors for d^25^Mg are larger than for d^26^Mg, probably because ^24^MgH^+^ varied spatially ~ (±4‰ to 5‰) in a manner consistent with minor differences in the matrix (details in supporting information Table A4). The measured (d^25^Mg, d^26^Mg) was (−14.4‰, −30.2‰), becoming (−11.4‰, −24.4‰) after correction for gravitational settling, so d^26^Mg data (per amu) are lighter than d^25^Mg by 0.7‰, which is within 1 SE of both measurements.

In Fig. 10a, the reference (the DSM‐3 composition) is assumed to be both nebular and solar: that is, no gravitational settling is included in either the Genesis SW Mg or the CAI proxy model. In Fig. 10b, the DSM‐3 composition is corrected from nebular to “photospheric”: that is, the Genesis SW Mg and the CAI proxy model both include a correction for gravitational settling over the past 4.5 Gy. The solar physics models in Fig. 10b are left unchanged because it is not clear how changes in their assumed (nebular) initial conditions will propagate to the results.

In both Figs. 10a and 10b, the Laming et al. (2017) Model 1 (low expansion field line source) and the CAI proxy predict significantly less fractionation than observed. The ICD model is also significantly low in Fig. 10a; however, in Fig. 10b, the 3‰ per amu gravitational‐settling correction moves the data to within 1σ of the ICD prediction. In both Figs. 10a and 10b, the Genesis SW Mg data are within 1σ of the range of the Laming et al. (2017) Model 2 (high expansion field line source) fractionation model. Therefore, to completely understand the comparison of the measured SW data with the models, we need to understand how their predictions would shift with the inclusion of gravitational settling into their initial conditions. That is, would a 3‰ per amu gravitational‐settling correction in the initial conditions for both the ICD and Laming et al. (2017) models simply shift their predictions for ^Mg^∆_(sw‐p)_ by 3‰? If so, the comparisons would look like Fig. 10a, but both models and SW would be shifted +3‰. In contrast, if the change in the initial conditions for the models did not propagate, the comparison between SW and the models would look like Fig. 10b. Therefore, at this time, both the ICD and Laming et al. (2017) Model 2 are reasonable approximations to the observed Mg isotopic fractionation for the bulk SW. So, as hypothesized by Bochsler (2007), the gravitational‐settling correction has become a factor in the comparison of SW data with the model predictions of SW fractionation.

**Figure 11:**
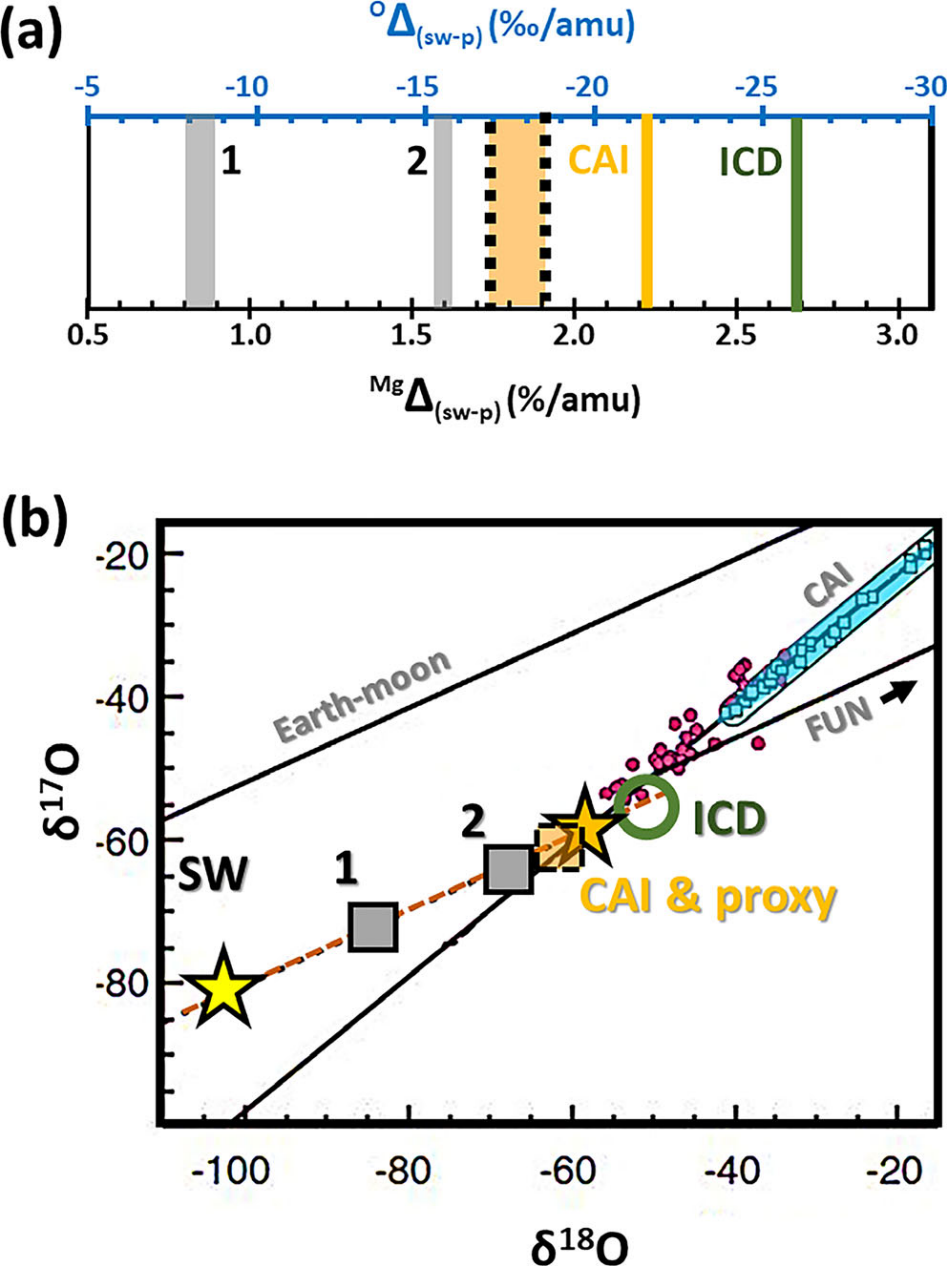
Comparison of predicted SW O fractionation models with Genesis data and possible implications after McKeegan et al. (2011) in (a) and (b), respectively. Gold box with dashed black edges gives the predicted effect of gravitational settling on O isotopes in the range of 3‰ (Marty et al. 2011) to 4.5‰ (Bochsler 2000). Yellow star and gold star in (b) are measured SW O and CAI values; gray lines and boxes are Laming et al. (2017) model predictions as labeled; green line and circle are the ICD model prediction. Marker sizes in (b) are bigger than the uncertainties.

Figure 11a gives the predictions of the models for SW O isotopes. No gravitational settling is included in the ICD, Laming et al. (2017) models, or the CAI line. Only the CAI proxy model (box bounded by dashed vertical line) gives the range of ^o^∆_(sw‐p)_ expected if a correction for gravitational settling is made (3‰ after Marty et al. 2011; 4.5‰ after Bochsler [2000]). Figure 11b illustrates how knowing a precise ^o^∆_(sw‐p)_ might affect the interpretation of the McKeegan et al. (2011) data. For example, the ICD model predicts a solar O isotope composition close to the hibonite component of some CAIs near the intersection of the CAI line with the FUN inclusions: if this is true, then the solar nebula was probably heavier than a CAI composition. In contrast, given gravitational settling in a Sun initially of CAI composition, the current photospheric composition should be closer to that currently predicted by Laming et al. (2017) Model 2 than to CAI (yellow box in Fig. 11b), with the caveat that gravitational settling was not a part of the Laming et al. (2017) initial conditions. So, again, the comparison of Genesis SW data with theoretical models predicting the fractionation of SW from the photosphere depends, in part, on the effect of the correction for gravitational settling on the solar composition used in the models.

In the future, measured Mg isotopic fractionation in each SW regime collected by the Genesis collector arrays (Burnett et al. 2003; Reisenfeld et al. 2007) may be able to further define the applicability of these theoretical models (and others) to define how much the SW is fractionation from the photospheric composition. It is also important to understand how shifts in the initial conditions of these models will affect the predicted fractionation. Of course, neither model as they exist today are perfect; that is, neither has been able to predict all mass‐dependent fractionation in Genesis samples (e.g., Heber et al. 2012; Laming et al. 2017 and references therein). Refinements to these (and other) models will be constrained by Genesis analyses and will almost certainly continue to improve our ability to use SW data to accurately and precisely measure both the composition of the solar nebula and the current composition of the Sun. This achievement will have significant ramifications for our understanding of active solar processes as well as our ultimate goal, the nebular composition and solar system formation (e.g., Burnett et al. 2019).

### Lessons Learned for Future Investigators Analyzing DoS by SIMS

Genesis Curation allocated our sample (#20732,2) for technique development. At that time, “technique development” on the DoS chip implied only cleaning and finding appropriate SIMS conditions. However, it quickly became clear that the DLC was not behaving predictably under the primary ion beam and that quantitative SW analyses required that we understand the variable response of this material during SIMS analysis. Accordingly, the technique development reported here is knowledge that will be required for other investigators using SIMS on DLC films to accurately and precisely quantify SIMS data. For example, our initial test indicated that H had little or no effect on our measurements (see Fig. 2, the mass 25 scan). Now we understand that the effect of H varies spatially with the properties of the matrix. If we had added H to our DLC standard and increased the MRP to 3554 M/∆M to resolve (^24^MgH)^+^, then (1) more of the analyses (SW_7, SW_8, SW_11, and possibly SW_12) would have been useful and (2) the error for the d^25^Mg results would likely have been reduced ~50% (Table A4).

We also now know that it would be useful to monitor silicon throughout analyses. If silicon is uniform, the small, potentially significant interferences (SiC_2_)^++^ and (SiMg)^++^ (Table A2) will either be removed by the background correction or be included in the calibration, respectively. However, if there is a silicon particulate present (e.g., Fig. 4b), the data may not be useful. To do this effectively and still collect enough SW counts, a higher transmission ion probe would likely be necessary.

A third “lesson learned” concerns our requirement of choosing SIMS conditions that insured analyses of the implant standard were not affected by dead‐time loss. Because SRIM fits our DLC data so well, it may be possible to use a higher primary beam current for more SW counts and then, for the same standard, use SRIM to correct the shape of the data for dead‐time loss. An alternative to increasing beam current would be to make a lower dose standard. A lower dose standard could still have been calibrated by ICPMS if a larger area had been implanted; that is, if an 8 inch diameter had been implanted instead of the 4 inch diameter, then two silicon wafers instead of one could have been included for the ICPMS calibration.

Most of the above suggestions require a higher beam current, which *may* have reduced the effectiveness of the ^12^C_2_^+^/^12^C^+^ parameterization, which is probably a function of current density versus impact energy of the primary beam. Luckily, if the ^12^C_2_^+^/^12^C^+^ does not behave effectively under new conditions, it is likely not the only useful parameterization (e.g., Fig. 8b used S/nA). Mapping matrix properties of the DoS wafer with Raman spectroscopy (e.g., Friedmann et al. 1994; Jurewicz et al. 2018) before analysis could mitigate the need for parameterization.

Other refinements for producing higher quality data would be (1) using a multicollector instrument or (2) measuring beam current for each duty cycle. Multicollection would eliminate the loss of counts during peak jumping, but has its own set of calibration problems. Beam current measurements for each duty cycle would be better for correcting for beam drift than the carbon yields used here (e.g., inset Fig. 9) as they also vary with matrix properties.

We invite future Genesis investigators to test these ideas and others.

## Summary with Conclusions

Quantifying the differences between the solar photosphere and the SW is central to Genesis Mission science, whose goal is to use the composition of the SW to determine the composition of the solar nebula. Mg isotopes are the key to this quantification (Burnett et al. 2019) because the composition of planetary materials (with the exception of radiogenic ^26^Mg) represents the solar composition at the ‰ level. Thus, any deviation of the SW Mg isotopic composition ≥1‰ from this baseline is due to solar processes. Comparison of measured fractionation in the SW with models of fractionation based on solar processes simultaneously (1) tests the model for the solar physicists and (2) identifies whether or not that model gives plausible results for the accurate and precise derivation of nebular composition from the measured SW composition.

This study reports fractionation of Mg isotopes between the solar photosphere and the bulk SW found by analyses of a Genesis DoS collector. Relative to DSM‐3, the (d^25^Mg, d^26^Mg) is (−14.4‰, −30.2‰) ± (4.1‰, 5.5‰). This uncertainty is the 2ơ SE plus additional error to compensate for the variable IMF of DoS. If gravitational settling is assumed to have occurred in the solar convective zone over the history of the Sun, this absolute fractionation is corrected by 3‰ per amu to (−11.4‰, −24.2‰) for the fractionation of the bulk SW from the photosphere. These results are then compared with the predictions of SW fractionation models based on different physics assumptions, as well as the prediction technique used by Marty et al. (2011). Marty et al. (2011) corrected for fractionation in Genesis SW N isotopes using a modification of the McKeegan et al. (2011) CAI proxy for Genesis SW O, taking into account gravitational settling in the convective zone of the Sun over the past 4.5 Gy. Figure 10 summarizes the Mg results; Fig. 11 illustrates the possible implications for O isotopes.

Figure 10 shows that, in spite of very different core assumptions the Laming et al. (2017) Model 2 agrees with the measured SW Mg isotopic fraction within a 1ơ error, whether or not gravitational settling is assumed, while the ICD model agrees within a 1ơ error only if gravitational settling is assumed for the SW data. Other results deviate more than 2ơ from the data. Figure 11 gives all model results for SW O after McKeegan et al. (2011) as well as their potential cosmochemical implications.

New techniques (reported herein and in the supporting information) were required for quantifying our SIMS analyses. The DLC has a variable, complex structure. Areas of the film having different density, electrical conductivity, and composition result in spatially variable IMF, ion yield, and mass interferences during SIMS analysis. An important discovery was that the SRIM program excels at modeling the SW in DLC, demonstrating that there has been little or no movement of SW in DLC after capture and allowing for the mitigation of some mass interferences. Accordingly, the method of choice for data reduction (both corrections for background and surface contamination) was the minimization of χ^2^ fits of SRIM models to all SW depth profiles while using the same density and IMF for each isotope. Details of techniques used herein (including successes and pitfalls) are given in the supporting information.

The “lessons learned” from this study will enable more precise measurements of isotopic and elemental abundances measurements by SIMS analysis of Genesis DoS collectors in the future. These future measurements will provide fundamental insights into solar physics and enable a more precise derivation of the nebular composition from SW data.

## Supporting information


**Data S1.** Data (both SRIM fits and Raw data) as separate MS Excel files.

**Table A.** List of abbreviations in SOM with pointers to more information. A1: Sample preparation procedures. A2: Nature of the interferences. A3: SRIM fits: Determination of surface contamination and background. A4: Correlation of ‰‐level (^24^MgH)^+^ with variations in the matrix for SW_2, SW_3, SW_5, SW_6, SW_9, SW_10. A5: References for the A1–A4. A6: Data, plots, and residuals for SRIM fits SW_2, SW_3, SW_5, SW_6, SW_9, SW_10.
