# Supplementary material for: Magnesium isotopes of the bulk solar wind from Genesis diamond‐like carbon films
Source: Meteorit Planet Sci. 2020 Jan 22;55(2):352–75. doi: 10.1111/maps.13439 (PMC7079557; doi:10.1111/maps.13439)

The Mg Isotopic Composition of the Bulk Solar Wind from Genesis DoS Collectors: supporting material

Contents

Table A. Abbreviations in SOM with pointers to more information... 2
A1. Sample preparation procedures.. 3
A2. Nature of the Interferences... 5
A3. SRIM Fits: determination of surface contamination and background.................................. 10
A4. Correlation of ‰-level ($^{24}MgH)^+$ with Variations in the Matrix for SW_2, SW_3, SW_5, SW_6, SW_9, SW_10.. 16
A5. References for the A1-A4.. 18
A6. Data plots and residuals for SRIM fits SW_2, SW_3, SW_5, SW_6, SW_9, SW_10.................. 20
A7. Data (both Srim fits and Raw data) as separate MS Excel files.. 34

We hope that our technique-development work will excite future investigators to spend the effort needed to measure precise SW isotopic compositions in Genesis samples. SIMS analysis of DoS collectors has great potential for this work, despite the variable matrix properties. Still, to quantify each and every bit of data, there needs to be a way to standardize the endmembers of the solid solution that constitute the diamond-like carbon matrix. Then parametrization can give results for the intermediate mixtures.

More information (and direct assistance) on techniques, methods and available materials are available from the first author and at the annual Genesis Solar Wind Sample Analysis and Techniques Workshop. Some Genesis-specific standards (including the IMF standard used here) eventually may be allocated through Genesis Curation at JSC; if not, Curation will be able to refer users to the appropriate sample archive.

Table A: Acronyms, abbreviations, and definitions

Abbreviation	Full phrase	Role	Definition	References and resources
DLC	diamond-like carbon	Part of DoS wafer that collected and retained SW	A carbon film having tribological properties more diamond than graphite. Genesis films were anhydrous, >50% sp^3 bonds, dense (averaging ~3 gm/cc), effectively homogeneous in columns but spatially variable density, as well as electrical and chemical properties.	Robertson (2002)<br>Grill (1999)<br>Sullivan et al. (1998)<br>Friedmann et al. (1994)<br>Jurewicz et al. (2017)
DoS	Diamond-like carbon on silicon	SW collector	For Genesis, DoS wafers are DLC deposited on silicon using pulsed laser deposition by Sandia National Laboratory. The DLC collected the SW, the silicon supported the DLC film.	Jurewicz et al. (2003)
IMF	Instrumental mass fractionation	fractionates isotopes during analysis	Electromagnetic fields inside all mass spectrometers tend to separate isotopes so that one isotope is measured more efficiently than the others. In SIMS, IMF is affected by sample properties and electrical fields. This fractionation is mass dependent and is accommodated using an appropriate standard (see IMF standard).	Wilson et al. (1998)<br>Eiler et al. (1997)
IMF Standard	IMF Standard	Dos with known $^{25}Mg/^{26}Mg$ is used to correct IMF	Primary standard made for this study. Commercial implant of ^{25}Mg and ^{26}Mg into both DoS and silicon; then ICPMS performed by dissolution of silicon determines $^{25}Mg/^{26}Mg$ ratio precisely so that analysis can be used to correct for IMF when measured under the same conditions as the unknown (SW). *Also: IMF-Calibration standard; DoS standard; implant standard*.	Burnett et al. (2015)<br>Jurewicz et al. (2017)
MRP	Mass resolving power	To separate peaks of the species of elemental and molecular ions produced by the primary beam	Defined as the M/ΔM for two adjacent mass peaks needed to resolve them to 10%; calculated as the position (M) divided by the peak's width (ΔM) at 10% of is height.	Wilson et al. (1998)
SRIM	Stopping Range of Ions in Matter (program)	Program used to model implants	Freeware used internationally for both teaching and research concerning ions implanted into solids. Assumes homogeneous target material not previously damaged.	www.srim.org
SRIM fit	model for implant generated using SRIM (program)	distinguishing implant from surface contamination	Best fit (using Ψ^2) between SW measured vs. calculated from spacecraft measurements.	SOM (A3)<br>Jurewicz et al. 2019

A1. Sample Preparation Procedures

Sample preparation was of great importance to this study for prepare (A) the standard implant for IMF and (B) Genesis sample #20732,2. An overview of the methods used are given below. Note that preparation of Genesis collectors for analysis are specific to each collector type and, to a lesser extent, to each target element. Several alternative methods under development are currently available in LPSC abstracts (e.g., Paramasivan et al. 2018; Welten et al. 2018).

A1.A. Cleaning procedures preceding the ICPMS measurement

Flight spare materials were used, so not everything required cleaning before implant. For the few pieces of silicon handled previously, a solvent pre-cleaning using semi-conductor grade solvents was performed prior to implant. Moreover, this pre-cleaning was repeated after the implant for all of the silicon to remove any organic contamination acquired in the vacuum system during implant. This treatment entailed a sequence of ultrasonic cleaning in 60°C xylenes, followed by acetone, methanol, ultra-high purity water in order to remove organic material deposited during the implant. This step also removed any adhesive remaining from the implant step.

ICPMS was performed in the Isotope Cosmochemistry and Geochronology Lab at ASU. The implant was uniform over all materials on the plate, so implanted silicon (as a proxy for the co-implanted DLC) and a silicon control were prepared for digestion to measure the isotopic ratio. The digestion and subsequent measurement used their trace-element grade solvents. The preparation for digesting both the control and implanted silicon for analysis consisted of an aggressive cleaning to remove of any surface contamination. The three steps in this cleaning was a variation of the common semiconductor RCA technique. They were:

(1) 5:1:1 H_2O:(30% NH_4OH solution): (30% H_2O_2 solution). 60°C, 12 minutes (intermittent ultrasonic used)
(2) 100:1 H_2O:(50% HF solution). 4 minutes in solution.
(3) 6:1:1 H_2O: (50% HCl solution): (30% H_2O_2 solution). 10 minutes in solution

The control and the implanted silicon were processed as separate batches. After the cleaning, the solutions for steps (1) and (2) for each batch were combined and then analyzed to ensure that the silicon in that batch was not leached during the cleaning. Digestion of the samples used an HF:HNO3 solution held in pre-leached high-quality PTFE (Savillex) container. The blank silicon (the first batch) consisted of several large pieces, which caused difficulties with the digestion. Accordingly, the implanted silicon (the second batch) was cleaved into 6 smaller

pieces prior to digestion. Cleaving entailed pressure at one edge by a diamond-tipped scribe while acid-leached forceps pinned the silicon by another edge (at a line of cleavage from the scribe) on a clean, soft surface (dacron class 10 cleanroom wipes). Any silicon powder created by the process of cleaving was blown off using air pressurized by a bulbed pipette.

A1. B. Cleaning of DoS (both standard and GENESIS #20732,2) for SIMS Measurement

The DoS for the standard implant had been attached to the plate for implant using a carbon-based SEM cement (DAG –e.g., "PELCO® Conductive Carbon Glue") strong enough to hold wafer fragments throughout shipping and handling. Post implant, the material was easily removed from the plate by slipping a razor blade under the edge of the fragment; however, some of the carbon cement continued to adhere to the back. This excess DAG was wiped from the backs using Kimwipes™ with xylenes followed by acetone.

A pre-cleaning followed that was similar to that used for the silicon. Again, the pre-cleaning used CMOS-semiconductor-grade solvents in an ultrasonic cleaner, but for DoS the ultrasonic cleaner was attached to a Variac® that lowered the input power to about 25%. (If full power was used, there was a significant chance that the DLC collector film of the DoS would delaminate from the silicon substrate). The cleaning sequence was as follows: xylenes at 70C for 10 minutes, then acetone to methanol to UHP water for two minutes each. A 2%- Micro-90® precision cleaning solution at 60C (10 min) in the (reduced power ultrasonic) was then used to remove particulates that had been loosened but not removed; then the pieces were rinsed in UHP water. ***Note that Micro-90® reacts with silicon***. Since the silicon in DoS just holds the DLC collector, the minor damage to the back from the Micro-90® is not an issue and there was no evidence of the solution reacting with the DLC film. However, Micro-90® should ***never*** be used with silicon (or silicon-on-sapphire) collectors.

After the pre-cleaning above, both the standards and Genesis #20732,2 were precision-cleaned using a modified RCA cleaning technique after Sinha (2002). Step 1 was an $NH_4OH:H_2O_2:H_2O$ (0.5:1:10) at 60C for 8 minutes, then rinse; Step 2 was an etch in $HCl:HF:H_2O$ (0.7:1:100) for 4 minutes, follwed by a rinse, and blown dry with a pippette.

A2. Nature of the Interferences in these data

Diamond-like carbon has chemical properties that negate much of the wisdom gained by the analysis of silicate minerals, or even other engineering materials. Carbon in general can be very reactive and tends to make hydrated organic complexes and gaseous oxides readily, but the high-stress components of the DLC, as well as the fact that it is anhydrous, makes the carbon thermodynamically unstable and even more reactive. For example, our results indicate that the usual ^{24}Mg-H interference is practically non-extant in DLC unless silicon is present. For example, It is possible that the small (~3‰ ^{24}Mg-H) peak observed in low-silicon DLC represents the reactivity of the strongly-bonded, intrinsic H in the (nominally anhydrous) DLC and that the implanted H ions have gone to gas and organic secondary ions instead because their internal energy is quite different from the lithophile trace elements present in the DLC.

Both the interferences encountered and the backgrounds present depend on the presence (or lack thereof) of silicon as a minor component. This will be shown by the Raman results presented later, as well as the observations described below. Although a problem for deriving internally-consistent results, these matrix effects provide a window into the underlying nature of the DLC that cause these effects (Jurewicz et al., 2017) as well as the opportunity to identify (and, in the future, to avoid or mitigate) the interferences encountered.

A2.A Deriving Information on matrix effects from SW_12 and SW_11

Much of the information on interferences present in this SW data set can be derived from a study of SW analyses SW_12 and SW_11. In review: these interferences are present because the MRP used for the SIMS analysis was 1604 M/ΔM as it was assumed that ^{24}Mg-H would be the major interference, and that was small, constant and could always be subtracted after the data reduction. Accordingly, any molecular secondary ions requiring an MRP > 1604 M/ΔM were definitely not resolved from the masses of ^{24}Mg, ^{25}Mg and ^{26}Mg and any abundant molecular secondary ions requiring an MRP slightly $\leq$ 1604 M/ΔM would not be completely resolved. Instead of being constant, these unresolved mass peaks and backgrounds varied from analysis to analysis. Rather than being random, most of these variations formed a strong trend of "anti-correlated" interferences (i.e., as the ^{25}Mg/^{24}Mg ratio decreases, the ^{26}Mg/^{24}Mg ratio increases) starting at the terrestrial fractionation line (TFL) and extending linearly to the analysis SW_12 (Fig. A2.1). Note: analyses without these interference follow the TFL due to the variable IMF.

SW_12 gives the first clue to the identities the molecular interferences. All of the SW_12 depth profiles are oddly-shaped and *cannot* be modeled by SRIM as a simple implant (see Fig. A2.2). The depth profiles are, however, consistent with an implant plus a Mg-bearing particulate

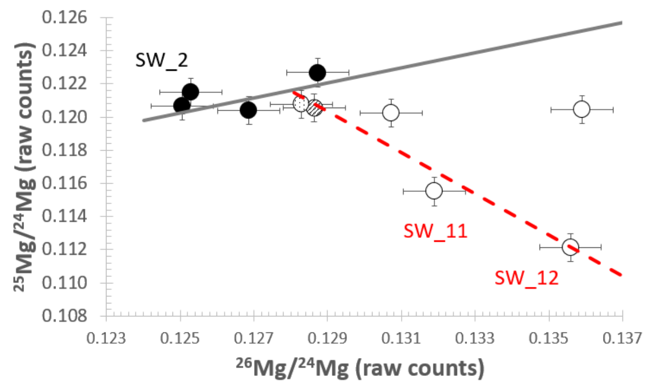

Figure A2.1. Three isotope plot. Solid line is terrestrial fractionation line; dashed red line mark anti-correlated interferences (seen in analyses with open markers and possibly patterned markers). See also Fig. 5 in text.

entrained ~25 nm below the collection surface in the DLC. Even if the particulate is anhydrous, logic suggests that a dust particle containing excess terrestrial Mg should increase the ^{24}Mg-H interference since there would be significantly more ^{24}Mg to react with the large amount of SW H; however, the observed ^{25}Mg/^{24}Mg ratio decreases. Accordingly, ^{24}Mg-H is not the *primary* interference in the ^{25}Mg/^{24}Mg ratio; the largest interferences present are at mass 24. Possibilities for the interference at mass 24 include the tail of the $^{12}C_2^+$ peak, $^{16}O_3^{++}$ (generated by the abundant ^{16}O of the primary ion beam), and doubly-charged silicon hydroxides. Note that even though the analysis (SW_12) samples a particulate that is likely entrained dust containing isotopically terrestrial Mg, it's ^{25}Mg/^{24}Mg ratio is the furthest from the TFL.

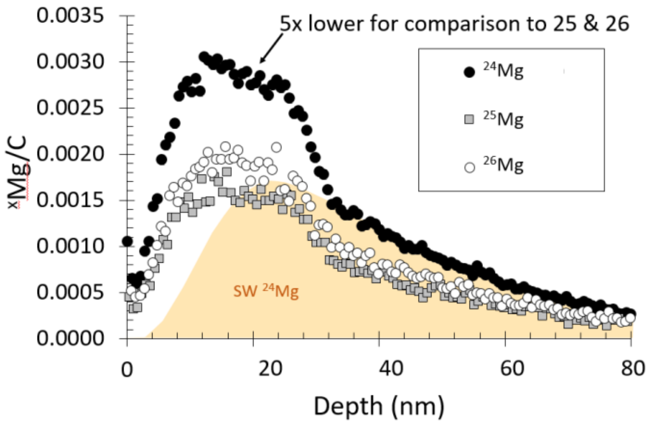

Figure A2.2. SW_12, all three depth profiles (24Mg is scaled for ease of comparison). Yellow shadow is the expected SW ^{24}Mg profile. See Fig. 4 in text for more details.

Interestingly, Fig. A2.1 demonstrates that as the ^{25}Mg/^{24}Mg ratio decreases, the ^{26}Mg/^{24}Mg ratio increases. Therefore, there must also be a significant interference at mass 26 too, even though $^{13}C_2^+$ and CN^+ should been completely resolved at an MRP of 1604 M/ΔM. A possible interference is $^{12}C_3O^{++}$, whose presence would constitute a uniform background. If $^{12}C_3O^{++}$ is present at mass 26, then $^{12}C_3N^{++}$ is likely present at mass 25. I fact, that correlation is consistent with Table 1 (in the text) which indicates that only two of the standard analyses have backgrounds at mass 26, but these are positively correlated with an interference at

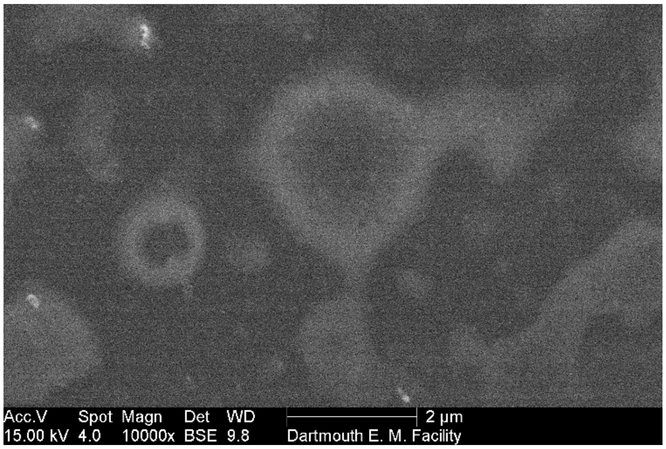

Figure A2.3. SiC exsolving from DLC locally, as seen on the walls (but not the floor) of this SIMS analysis pit in backscattered electron imaging from Genesis sample (after Jurewicz et al. 2017).

mass 25. Other possibilities at mass 26 (only) are $^{25}MgH^{+}$, $(^{24}Mg^{28}Si)^{++}$ and $(^{28}Si^{12}C_2)^{++}$. Two of the three interferences are directly related to the Si content, and silicon hydroxides were postulated (among other possibilities) for the interference at mass 24, so there may be an indirect relationship as well. When the silicon in the DLC is uniformly distributed, $(^{28}Si^{12}C_2)^{++}$ could be removed as background; however, Fig. A2.3 shows that not all of the silicon is uniformly distributed, even when it is not an entrained particulate. Localized silicon-rich features like the one seen in Fig. A2.3 would be removed by the surface correction if it was adjacent the surface; otherwise, if it was significant in size, it would skew the SRIM fit. The other two interferences are based on SW Mg ($\pm$SW H). Accordingly, this interference would change in magnitude with the Mg depth profiles and would definitely not be removed as background. Since the SRIM profile for SW H is different than that of SW Mg, a Mg-H interference might skew a fit to a Mg SRIM profile; however, the $(^{24}Mg^{28}Si)^{++}$) shape would not be recognized in the SRIM fit. Note that the $(^{24}Mg^{28}Si)^{++}$) interference cannot be resolved by any commercial SIMS instrument. If it is present, removing it from DLC would require a parametrization with the silicon concentration.

To further unravel the nature of the interferences, isotopes were calculated for SW_11, the analysis adjacent SW_12 showing strong “anti-correlated interferences” (Fig. A2.1), but which did not appear to have an embedded magnesium-bearing particulate. The resulting SRIM fits were as good as any of the six analyses that plotted adjacent the terrestrial fractionation line (details in Table 3 in text), but the resulting isotopic composition was highly anomalous: (-35, +81). So, at least one significant interference is proportional to the SW Mg.

If we assume we know the SW isotopic composition, then we can make estimates of the relative interferences at masses 24, 25, 26. Although the solutions are non-unique, they provide insight into what is happening during the analyses. So, assume that the true SW isotopic composition is, say, (-13, -30). Then, the fit to the SW_11 data requires that the Mg^{+} secondary ion yield must go down ~10%; i.e., with silicon present, additional Mg goes into Mg-bearing secondary molecular ions, perhaps formation of Si_x-O_y-H_z compounds that are not necessarily seen as interferences. The result is a decrease in the Mg ion yield. In fact, decreases in ion yield in analyses with anti-correlated interferences have been observed in our ongoing, *in house* work on Mg fluences, the preliminary result of which was reported in Jurewicz et al. 2019.

A2.B. Confirmation of the Role of Silicon by Raman Spectroscopy

Raman spectroscopy by 532 nm laser was used to investigate the role of silicon in the observed matrix effects. Since this work was performed after the analyses, the Raman spectrum from the edge of the sputtered crater was assumed to be representative of the (missing) analyzed area. The Raman spectra of interest were the carbon-carbon bonds and silicon-carbon bonds. In our

SW collector, 20732,2, none of the Raman profiles from the collector's surface showed a distinct crystalline silicon peak. A weak silicon signal was inconclusive, as it could originate in either the DLC or the silicon substrate. So, this work used the strong carbon (G, D) peaks and the silicon carbide Raman scattering for comparisons of the matrices. The signal for silicon carbide is weak under a 532 nm laser (Janz, 2006), so any signal likely represents bonding within the DLC.

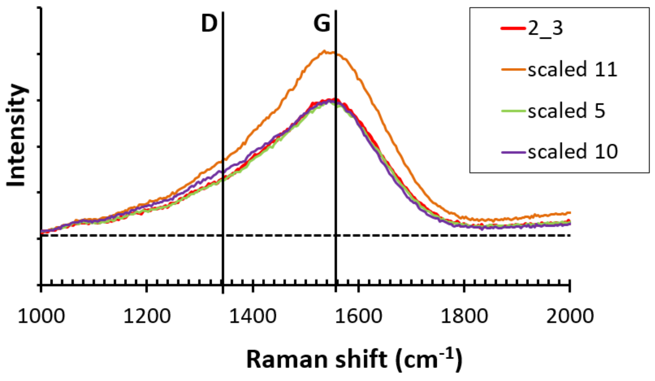

Fig. A2.4. Raman shifts for surfaces adjacent analysis pits that plotted on the terrestrial fractionation line vs. the pit of SW_11. Analyses near the TFL are nearly indistinguishable, but SW_11 has a significantly higher G-peak, suggesting a higher graphitic component to the SW_11 DLC. Dashed line gives the SW_2, 3 background intensity at 1000 cm^{-1} which was the value to which the spectra of the other analyses were scaled.

The Raman shift range 1000-2000 cm^{-1} fingerprints carbon bonding and textures in diamond-like carbon. To compare spectra, Raman intensities are first scaled (i.e., multiplied by a constant factor) so that all of them have the same intensity at a specified Raman shift (this work: 1000 cm^{-1}). Fig. A2.4 gives the 1000 – 2000 cm^{-1} Raman shift for spectra taken adjacent the analysis pit for SW_11 (which showed interferences but did not appear to contain an entrained Mg-bearing particulate) as well as Raman spectra for DLC adjacent the pits of four (SW_2 and SW_3 were adjacent) analyses whose error bars intersected the TFL. The dashed horizontal line is the measured intensity of 1000 cm-1 of SW_2,3. G ("graphite") and D ("diamond" or "disorder") are marked (interpretations based on Chu and Li 2006). All spectra taken adjacent analyses that plotted on the TFL in Fig. A2.1 are effectively identical; the G-peak of the spectrum taken adjacent SW_11 is clearly more intense. The high G-peak suggests that the DLC of SW_11 was more graphitic (a higher sp^2/sp^3 carbon-bonding ratio) than the other DLC analyses.

The Raman spectra shift range of 750-1000 cm^{-1} is where the (weak) signals for SiC occur: i.e., paired crystalline SiC peaks at (780 cm^{-1}, 960 cm^{-1}) and a "hump" for amorphous SiC centered ~840 cm^{-1} (interpretations after Janz, 2006). Fig. A2.5 gives the Raman spectra of SW_11 and SW_2,3 from Fig. A2.4, but centered on the 750 – 1000 cm^{-1} range (here, scaled to the intensity of SW_2,3 at a wavelength of 890 cm^{-1}).

SW_11 contains a weak amorphous SiC hump as well as a suggestion of weak, paired crystalline SiC peaks but the reference spectrum (SW_2,3) does not. So, it is quite possible there is an unresolved $(^{28}Si^{12}C_2)^{++}$ peak at mass 26; certainly a second interference from $(^{28}Si^{24}Mg)^{++}$ is not precluded if SW_11 contains excess silicon.

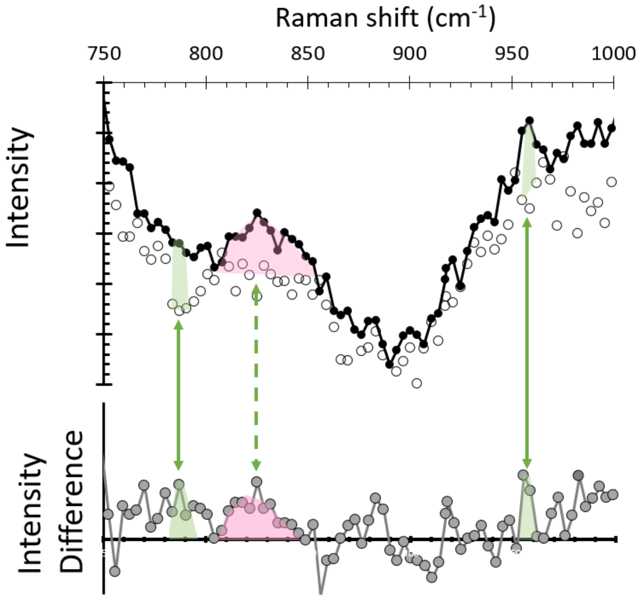

Fig. A2.5. Raman spectra fingerprinting (weak) SiC features. Top plot is Raman spectra (SW_2,3 open circles; SW_11 black). Bottom plot is the difference between spectra. Solid lines point to green-filled crystalline SiC; dashed line point to pink-filled amorphous SiC hump (after Janz 2006).

So, Raman spectroscopy detected silicon carbide bonds in an area of DLC matrix consisting of a more graphitic carbon. The main text gave evidence that that silicon changes the mode of sputtering under the O_2^+ beam. The Raman suggests that the silicon effects the DLC bonding during its fabrication, whether or not a large particulate is present. Some silicon may break C-C bonds, thus making the carbon more mobile so that it can increase density and form sp^3 bonds under the high internal stresses (e.g., Robertson 2002); however excess silicon will promote growth of SiC and, apparently, result in a more graphitic matrix.

A3. SRIM Fits for the determination of surface contamination and background

Because the calibrated implant used for the IMF standard used minor ions, it was straight-forward to correct for background and surface contamination. Specifically, first background was subtracted, and then the implant was modeled using SRIM* to separate the surface contamination. Moreover, the high dose made the calibrated implant relatively insensitive to contamination in general. The only issue encountered was the need to accommodate the spatially variable density of the sample, which was needed for the SRIM model. Surface corrections for the SW collectors were more problematic. Because the SW array collectors have only ~1.5×10^{12} Mg atoms per cm^2 distributed though the 200 nm depth, the SW signal is necessarily low. This low concentration is why the interferences discussed above had such a significant effect on the resultant SW values in the trend of anti-correlated interferences (Fig. A2.1). Additionally, because the tail of the solar wind implant is both low in concentration and extremely long, the analyses did not run to the end of the solar wind implant, so the observed "background" (<1 cps) contained solar wind counts. The solution to these data-reduction issues was to iteratively fit the SW data with a SRIM profile, subtract background, and re-fit the data until a minimum deviation from SRIM was achieved. Details of both the standard and solar wind data reduction processes (outlined in the main text) are given below.

A3.A. Data reduction for analyses of the calibrated implant (IMF standard)

The IMF calibration standard was a dual 75kV implant of ^{25}Mg and ^{26}Mg, each isotope having a nominal fluence of 1×10^{14} atoms/cm^2. A very small amount of ^{24}Mg was also implanted as ^{24}MgH, creating an "accidental implant" of ^{24}Mg less than the SW fluence. Background was measured directly during overnight analyses and was simply subtracted. The surface correction was only slightly more complicated. Each analyses of the standard included a depth profile for ^{24}Mg which sampled the accidental implant as well as any ion-mixed surface contamination. By fitting the ^{24}Mg accidental implant using SRIM, the amount of ^{24}Mg in the depth profile due to the accidental implant could be separated from the amount due to the surface contamination.

The SRIM calculation requires the input of both a matrix composition and a density. The 75kV Mg implants into the flight-spare DLC were best modeled using SRIM with a matrix of SRIM 2008 catalog material #906 nuclear-grade graphite with the SRIM-provided compound correction of 0.8684 (Jurewicz et al. 2017). This catalog matrix was all carbon, but the bonding and the compound correction made the peaks slightly narrower than assuming a matrix

* The SRIM program used to model the small ^{24}Mg implant is freeware software (www.srim.org). Designed for teaching undergraduate students about ion implantation, it easily models mono-energetic implants of known energy into uniform or layered solids of known composition and density.

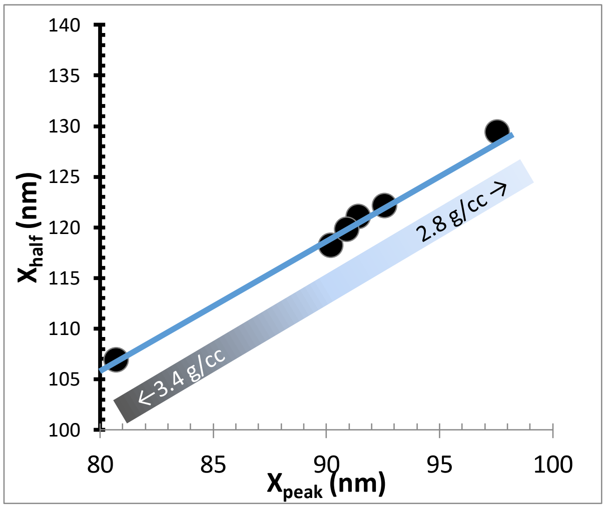

Fig. A3.1. Plot of the shape factors of the six standard analyses (markers) and the SRIM model for SRIM-catalog graphite having densities of 3.4 – 2.8 gm/cc (line) after Fig. 3 of Jurewicz et al. (2017). The density needed to model the analysis was estimated using the position of the markers relative to the density on the bar.

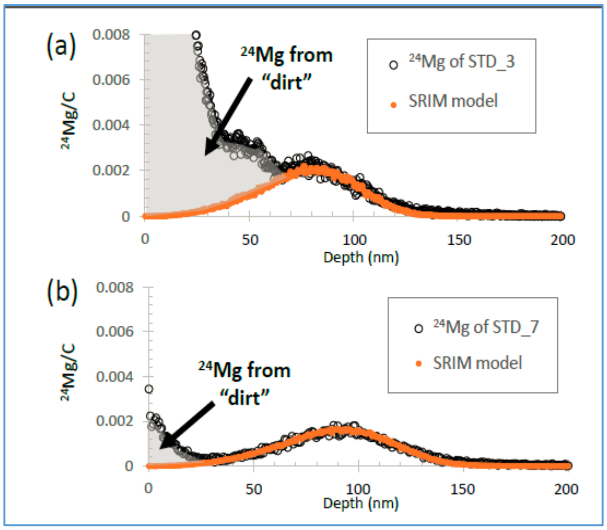

Fig. A3.2. Two example SRIM fits and their results. ^{24}Mg depth profiles in the standard (a) STD_3 and (b) STD_7 plotted with the SRIM model for the accidental implant. Excess 24Mg at the surface of the collector is surface contamination. Note the difference in shape of the SRIM models: (a) STD_3 is 3.4 gm/cc but (b) STD_7 is 3.0 gm/cc.

of elemental carbon. Density was then estimated using the plot in Fig. A3.1.: the line is the trend of SRIM models that use #906 nuclear-grade graphite matrix of different densities, and the black circles represent a parametrization of the actual ^{24}Mg depth profiles from the analyses. The parametrization is what Jurewicz et al. (2017) defined as "shape factors"; i.e., the depth of the implant peak (X_{peak}) and the depth when the tail of the implant drops to half of the peak and (X_{half}). The initial density input into the SRIM program was that of the closest density as estimated from the bar. This value was modified (slightly) if the original density estimate did not result in a good match with the data.

As an example of the extreme variability in the DLC and the parameters required for the SRIM fit, Figure A3.2 illustrates the results of two SRIM fits to the accidental implant. **(a)** is the worst contamination in any of the profiles; **(b)** is low in terms of contamination, but more typical. The model for (**a**) uses an average DLC density of 3.4 gm/cc while (**b**) uses a density of 3.0 gm/cc. Interestingly, these two analyses were taken less than 1cm apart on the same fragment of DoS. So, the question is whether or not both of these SRIM densities are reasonable. Sullivan et al. (1998) reported that the density of the DLC from Sandia National Labs (the Genesis source) was ~3.0 gm/cc, estimated by X-ray reflectivity. Robertson (2002) estimated that heavily sp3-bonded anhydrous DLC has a density of ~3.1 gm/cc. Both of these estimates are closer to that of the SRIM model for STD_7 (3.0 gm/cc) rather than the SRIM model for STD_3 (3.4 gm/cc). However, an inspection of the analysis pit for STD_3 using backscattered electron imaging found the feature presented in Fig. A3.3 – a diamond

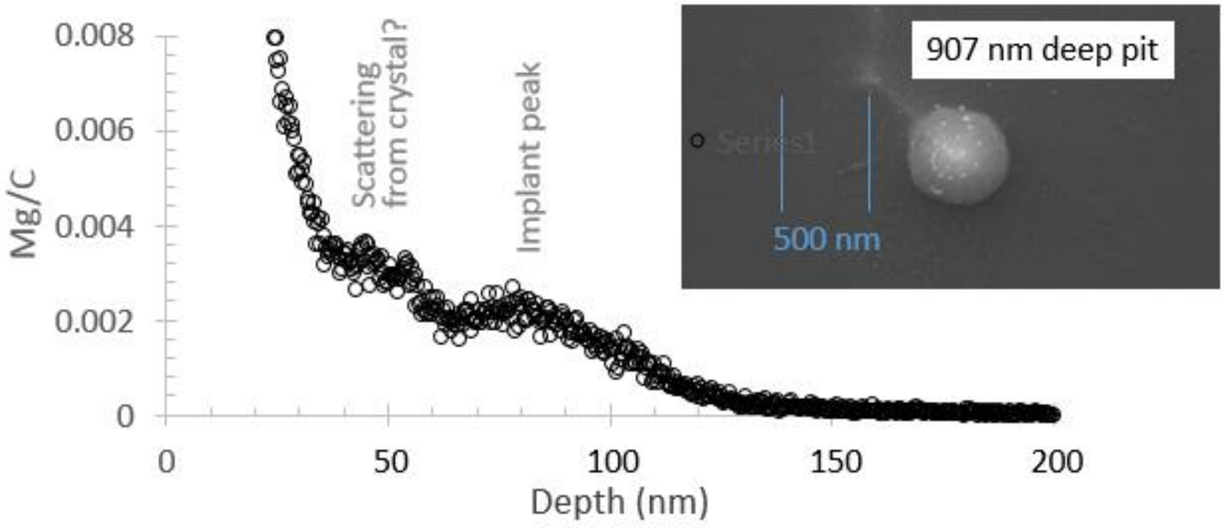

Fig. A3.3. Plot of ^{24}Mg depth profile from STD_3 with inset showing the bottom of the SIMS pit in backscattered electron imaging (after Jurewicz et al 2017). The diamond crystal is still euhedral after 12 hour of sputtering and pure diamond is one of the most electrical insulating compounds known.

crystal greater than 0.5 µm in diameter. Clearly, this diamond crystal, as well as the small diamonds (and probable nm-sized sp^3 domains) in the surrounding matrix indicate that an average density of ~3.4 gm/cc is reasonable for this analysis. (As an important aside: this feature also explains the extreme and somewhat oddly shaped surface contamination remaining after the SRIM fit. The crystal was definitely large enough to charge during analysis and probably scattered some of the incoming O_2^+ ions. Accordingly, future researchers should be aware of possible occasional beam scattering and small beam misalignments when analyzing DLC using a SIMS).

After the SRIM fit to the ^{24}Mg depth profile, the surface contamination was isolated as in Fig. A3.2. The counts of ^{24}Mg from contamination were integrated and assumed to be from isotopically normal terrestrial Mg. This last assumption allowed quantitative surface corrections to the ^{25}Mg and ^{26}Mg implants. The assumption was not perfect, because the actual counts of ^{25}Mg and ^{26}Mg implants were modified 3-5%/amu by the instrumental mass fractionation. However, since the correction for surface contamination was small relative to the implant, even for STD_3 ("dirt" = ~1% of total ^{25}Mg, ^{26}Mg counts), neglecting the IMF in the surface contamination was not considered an issue.

A3.B. Data reduction for the Solar Wind Depth Profiles

Data reduction for the solar wind depth profiles was a multi-step, iterative process; however, the first step is similar to that of the data reduction for the standard (IMF) implant, above. As mention in the introduction to this Appendix, background was not directly measureable because of the SW counts in the tail. So, background was not immediately subtracted; instead, the initial step was to run a SRIM profile with an estimated density of the DLC. Figure A3.4 gives the parametrized SW ^{24}Mg measurements versus the SRIM models that allowed the first estimate for density of the DLC sampled by the SIMS. Unlike the 75 kV implants, the matrix used for SRIM was elemental carbon and the energy distribution was derived from spacecraft data. A single SW SRIM data set (depth per implanted ions #1 – 99999) calculated for a density of 2.85 gm/cc was used. Each ion in this raw data set was first scaled in depth to the appropriate density. Then, the data were binned to the depths calculated for each SIMS duty cycle using the Frequency (x, y) function in MS Excel. Finally, the distribution was scaled in intensity to match the measured Mg cps.

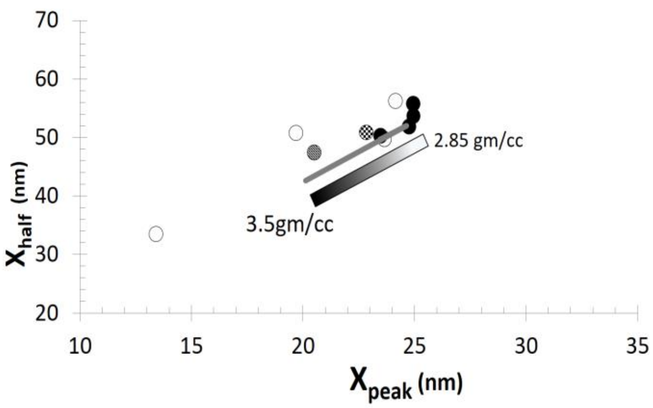

Fig. A3.4. Plot of Shape factors for all SW ^{24}Mg depth profiles vs. SRIM models. Open markers were not reduced; other markers represent analyses near the TFL (see text Fig. 6). Line is the locus of SRIM results for SW into carbon at densities 3.5 gm/cc to 2.85 gm/cc. Parametrization as in Fig. A3.1.

Fitting the SRIM models to the SW data was done by Ψ^2 give as:

$$X^2 = \sum_{n=x1}^{n=x2} ((profile - SRIM)^2)/SRIM$$

Where x1 to x2 is a range below the ion-mixed terrestrial contamination but above the point in the depth profile where the counts are primarily background. This range was usually ~30 nm – 150 nm for ^{24}Mg and a bit shorter (35 nm – 120 nm) for ^{25}Mg and ^{26}Mg.

Figure A3.5 gives a plausibility argument for the fundamental assumption of the Ψ^2 fit: that the best-fit SRIM model perfectly fits the true solar wind profile. Both Fig. A3.5(a) and (b) give the measured, uncorrected data for SW_2. (a) also gives the measured contamination profile

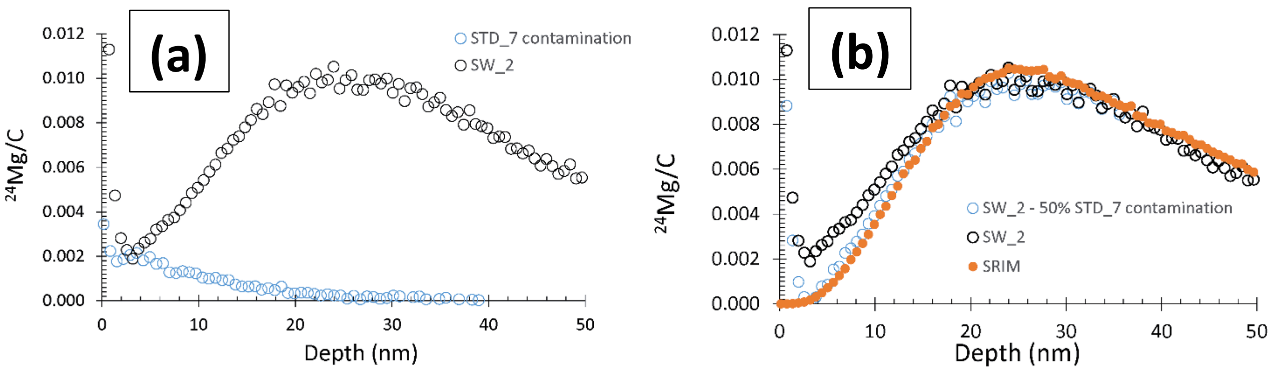

Fig. A3.5. Comparing surface correction using SRIM with surface correction using contamination from a standard analyses. (a) depth profiles of raw ^{24}Mg profile of SW_2 and raw ^{24}Mg surface contamination calculated for STD_7. Density of both assumed to be 30 gm/cc. (b) raw ^{24}Mg profiles of SW_2 and SRIM model. Also plotted is an empirical curve in which half the intensity of the STD_7 contamination from (a) was subtracted from SW_2.

for SW_7, and (b) includes both a SRIM fit to SW_2 and a plot of “SW_2 – 0.5*(STD_7 contamination)”, where the contamination is the profile from (a). Note the correlation between the SRIM fit and the subtraction of observed ion-mixed contamination. Of course, this correlation will not be true for every case as some analyses have sampled near-surface particulates: SW_2 and STD_7 were chosen because their low levels of surface contamination approximated thin films being mixed by the SIMS analysis.

Once a reasonable SRIM fit was determined for the SW ^{24}Mg depth profile, the distribution of counts in the tail could be estimated. The difference between the measured counts and the SRIM-derived counts was the background. The background was subtracted and the SRIM model was then re-fit to the background corrected data. This process was repeated until a minimum X^2 value was found for a combination of density, intensity, and background for the SW ^{24}Mg profile.

The process for the surface and background correction (i.e., the SRIM fit) for the ^{25}Mg and ^{26}Mg depth profiles were performed the same way as for the ^{24}Mg depth profile with a couple of modifications. First, it was assumed that the density determined for the SW ^{24}Mg was valid for the SW ^{25}Mg and SW ^{26}Mg implants. Because the counts were an order of magnitude lower for these depth profiles, the surface corrections for the ^{25}Mg and ^{26}Mg profiles were calculated using the ion-mixed contamination of the ^{24}Mg depth profile multiplied by the terrestrial isotopic ratio and then corrected for IMF. The determination of IMF for each profile is given in the main text (*Results*, including Fig. 8).

As a final note: it was discovered that the set of SRIM fits for each analysis occasionally had several local minimums for the Ψ^2 fits, probably due to the presence of interferences or simply because of the low counting rates. That is, starting with a different assumed density or background would give a different SRIM fit. In that case, the set of SRIM fits having the smallest % deviation from the measured data was used.

A4. Correlation of ‰-level $(^{24}MgH)^+$ with Variations in the Matrix for SW_2, SW_3, SW_5, SW_6, SW_9, SW_10.

One factor for selecting appropriate analysis conditions for this work was the determination of the magnitude of the $(^{24}MgH)^+$ interference at mass 25. Since our IMF standard was anhydrous, this determination was made on a separate implant having ^{24}Mg:^{25}Mg at nominal 1e13 fluences (i.e. 1:1) and a nominal 1.8E16 of H at 10keV. The result (shown in Fig. A5.1) is was an observed interference of ~3‰. Because it was assumed that DLC would be homogeneous under the ion beam – or at least variations of all secondary ion species would be proportional – a low MRP (~1604 M/ΔM) was chosen to increase the counting rate for the SW, and it was assumed that the ~3‰ contribution from $(^{24}MgH)^+$ could be subtracted from the mass 25 data after the fact.

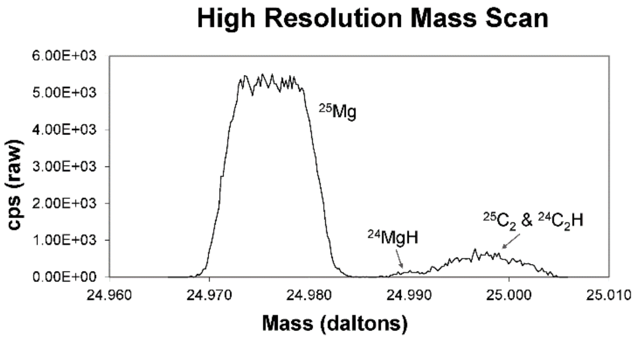

Figure A5.1. Mass scan at mass 25 from which the ~3‰ contribution from $(^{24}MgH)^+$ was estimated.

The initial SIMS results clearly indicated that the data contained variable contributions of interferences at all masses (see Section A2, especially Fig. A2.1). However, it was assumed that the six analyses nearest to the terrestrial fractionation line seen in the data contained minimal interferences, and that for these analyses a ~3‰ contribution from $(^{24}MgH)^+$ was a reasonable estimate. Moreover, the final ($d^{25}Mg$, $d^{26}Mg$) fell very close to the terrestrial fractionation line, a fact strongly suggesting that the ~3‰ estimate for $(^{24}MgH)^+$ was valid for these six analyses. However, the errors for the $d^{25}Mg$ and $d^{26}Mg$ data were not consistent with either a homogeneous material or an inhomogeneous material in which the variations in the ion yield for ^{25}Mg and $(^{24}MgH)^+$ were proportional (Fig. A5.2).

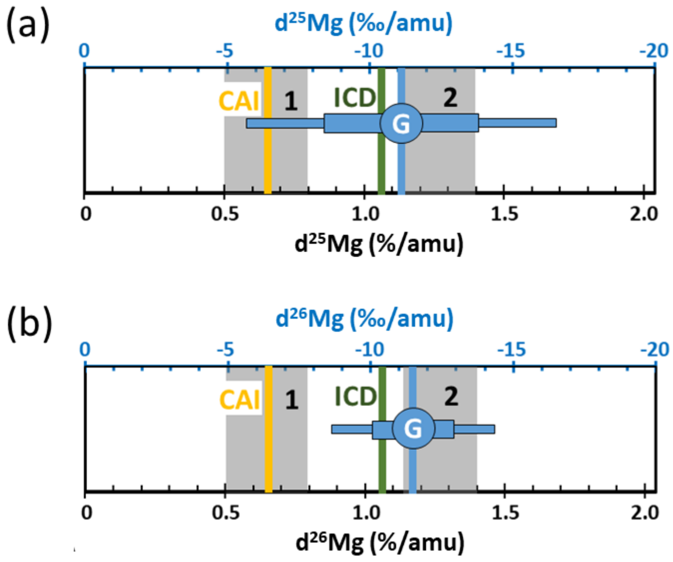

Figure A5.2. Comparison of mass fractionation results (per amu) from ^{25}Mg and ^{26}Mg in (a) and (b), respectively. CAI, ICD (vertical lines) and grey rectangles #1 and #2 are model results being compared with the Genesis bulk SW data, G, whose errors are given by the horizontal blue lines (thick = 1σ, thinner = 2σ; (see article, especially Fig. 10, for details).

In Fig. A5.2, the blue lines marked with a "G" indicate the Genesis bulk SW magnesium isotope measurements. The vertical line is the measured fractionation; the horizontal lines (thick and thinner) represent 1σ and 2σ errors, respectively. Note that the errors in (b) are larger than in (a) by almost a factor of 2.

The most likely reason for a larger error in the $d^{25}Mg$ is an issue with the quantification of $(^{24}MgH)^+$. During the instrument set-up, it was assumed that the magnitude of the measured interference would be constant relative to the intensities of $^{24}Mg^+$ and $^{25}Mg^+$. Assuming that all of the H comes from the SW, as do the Mg, species, was plausible. However, the larger

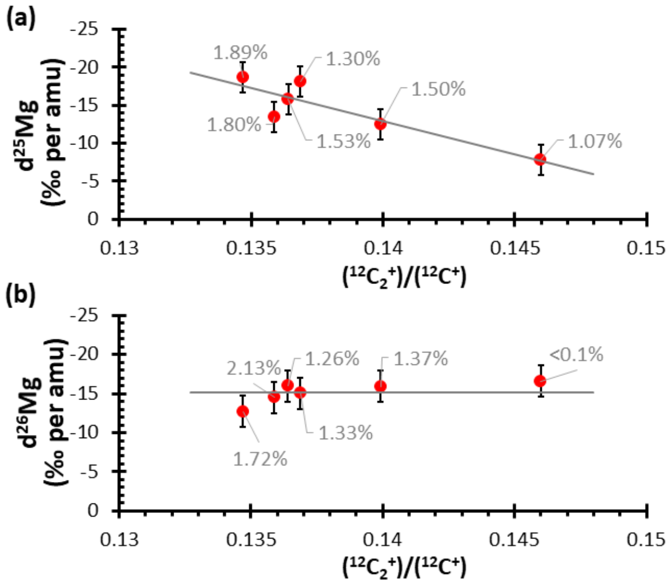

Figure A5.3. Mg isotope Fractionation in bulk SW prior to correction for gravitational settling in the convective zone. (a) derived from ^{25}Mg data (including (^{24}MgH)$^+$ interference) and (b) derived from ^{26}Mg data. Error bars are $\pm$ 2‰, the maximum observed difference in IMF estimated from two matrix-mapping techniques (Fig. 8 in text). Blue trend line of (a) must be sloped relative to the matrix parametrization ($^{12}C_2^+$)/($^{12}C^+$) to match data; trend line of (b) was forced to 0 slope, and the data are consistent to ~ $\pm$ 2‰. Labels are the SRIM fits to the ^{25}Mg data and ^{26}Mg data in (a) and (b), respectively.

errors for d^{25}Mg suggest that the large matrix effects discussed in A2 of this SOM also changes the ratio (^{24}MgH)$^+$/^{24}Mg$^+$ produced. To check that is a matrix-dependent variability in the calculated d^{25}Mg but not the d^{26}Mg, Fig. A5.3 plots the Mg fractionation per amu as determined by d^{25}Mg and d^{26}Mg, respectively vs. parametrization for matrix variability ($^{12}C_2^+$)/($^{12}C^+$). Note that the numbers here are the data reported in Table 4 of the text, and (unlike Fig. A5.2) *do not contain* the (3‰, 6‰) for gravitational settling in the convective zone of the sun.

The fractionation per amu in Fig. A5.3(a) shows a strong trend with the structure of the matrix, while the fractionation per amu in Fig. A5.3(b) does not. If there is a trend in the calculated d^{26}Mg with matrix structure, it is small relative to other possible errors, such as the IMF determination, beam current drift, and error incurred in finding the best-fit SRIM model.

A5. References for Appendix

Burnett D. S., Jurewicz A. J. G., Woolum D., Wang J., Paque J., Nittler L., McKeegan K., Humayun M., Hervig R., Heber V. S., and Guan Y. 2015. Ion Implants as Matrix-Appropriate Standards for Geochemical Ion Probe Analyses. *Geostandards and Geoanalytical Research* 39(3): 265-276.

Chu P. K. and Li L. 2006. Characterization of amorphous and nanocrystalline carbon films. *Materials Chemistry and Physics* 96: 253–277.

Eiler J. M., Graham C., and Valley J. W. 1997. SIMS analysis of oxygen isotopes: matrix effects in complex minerals and glasses. *Chemical Geology* 138: 221-244.

Grill A. 1999. Electrical and Optical Properties of Diamond-like Carbon. *Thin Solid Films.* (355-356): 189-193.

Friedmann T. A., Siegal M. P., Tallant D. R., Simpson R. L., and Dominguez, F. 1994. Residual stress and Raman spectra of laser deposited highly tetrahedral-coordinated-amorphous-carbon films. *Spring meeting of the Materials Research Society (MRS),San Francisco, CA (United States),4-8 Apr 1994.* Online: *www.osti.gov/scitech/biblio/10151495* Accessed 22 Nov 2016.

Janz S. 2006. Amorphous Silicon Carbide for Photovoltaic Applications. *online dissertation*: *www.ub.uni-konstanz.de/kops/volltexte/2007/3201*.

Jurewicz A. J. G., Burnett D. S., Wiens R. C., Friedmann T. A., Hays C. C., Hohlfelder R. J., Nishiizumi K., Stone J. A., Woolum D. S., Becker R., Butterworth A. L., Campbell A. J., Ebihara M., Franchi I. A., Heber V., Hohenberg C. M., Humayun M., McKeegan K. D., McNamara K., Meshik A., Pepin R. O., Schlutter D., and Wieler R. 2003. The Genesis solar-wind collector materials. *Space Science Reviews* 105(3-4): 535-560.

Jurewicz A. J. G., Burnett D., Rieck K., Hervig R., Friedmann, T., Williams P., Daghlian C. and Wiens R. 2017. Understanding heterogeneity in Genesis diamond-like carbon film using SIMS analysis of implants. Jour. Mater. Sci. 52(19): 11282-11305. DOI 10.1007/s10853-017-1267-3 (Open Access Paper).

Jurewicz A. J. G., Olinger C., Burnett D. S., Rieck K. D., and Woolum D. S. 2019. SW Mg from GENESIS: New Method of Data Reduction and Implications (*Abs #2353) 50th Lunar and Planetary Science Conference (e-poster).* ONLINE.

Paramasivan G. J., Sharma M., Jurewicz A., Burnett D. 2018. A Procedure to Cleanly Separate Solar Wind Osmium Embedded in Genesis Silicon Collectors (Abs #2886) *49th Lunar and Planetary Science Conference*.

Robertson J. 2002. Diamond-like Amorphous Carbon, Materials Science and Engineering Reports 37:129-281.

Sinha, D. 2002. Correlating chemical and water purity to the surface metal on silicon wafer during wet cleaning process Chem. Eng. Comm. 189: 974-984.

Sullivan J. P., Friedmann T. A., Dunn R. G., Stechel E. B., Schultz P. A., Siegal M. P. and Missert N. 1998. The electronic transport mechanism in amorphous tetrahedrally-coordinated carbon films. *Materials Research Society Symposium Proceedings* 498: 97-102.

Welten K. C., Bixler A. J., Nishiizumi K., Caffee M. W., Jurewicz A. J. G., Burnett D. S. 2018. Cleaning Studies of Genesis Sapphire Target 61530. (Abs #2660) *49th Lunar and Planetary Science Conference*.

Wilson R. G., Stevie F. A., and Magee C. W. 1988. Secondary ion mass spectrometry: A practical handbook for depth profiling and bulk impurity analysis. New York: John Wiley and Sons, Inc. 384 p.

SRIM fits to the 6 analyses along the terrestrial fractionation line

For each analysis:

- slide 1 = fit. Markers = SW data, line = SRIM fit
- slide 2 = residuals. Markers = (measured – srim fit), line = “0”.

SW_2 (-18.6448 -25.4576)

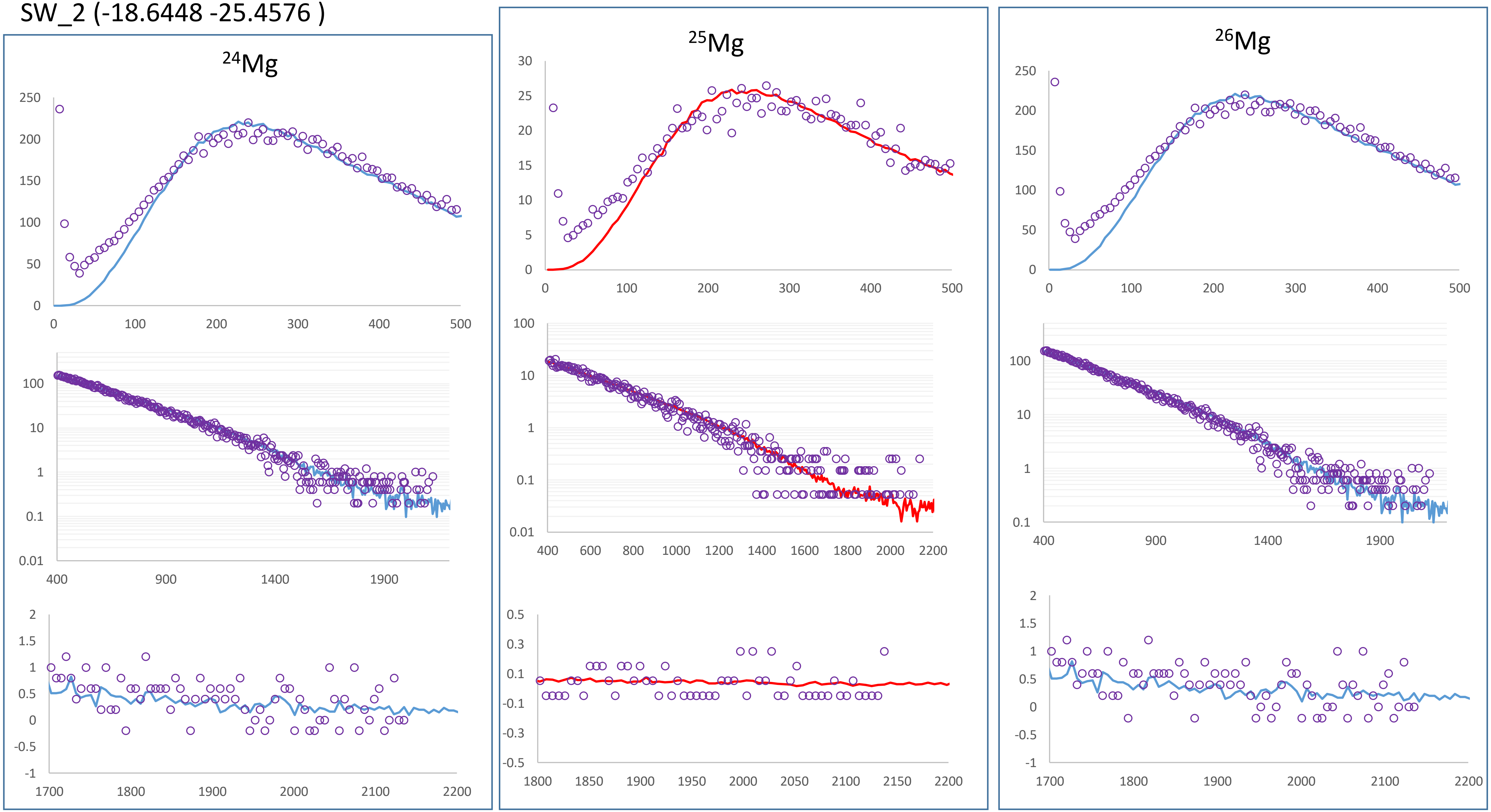

SW_2 (residuals)

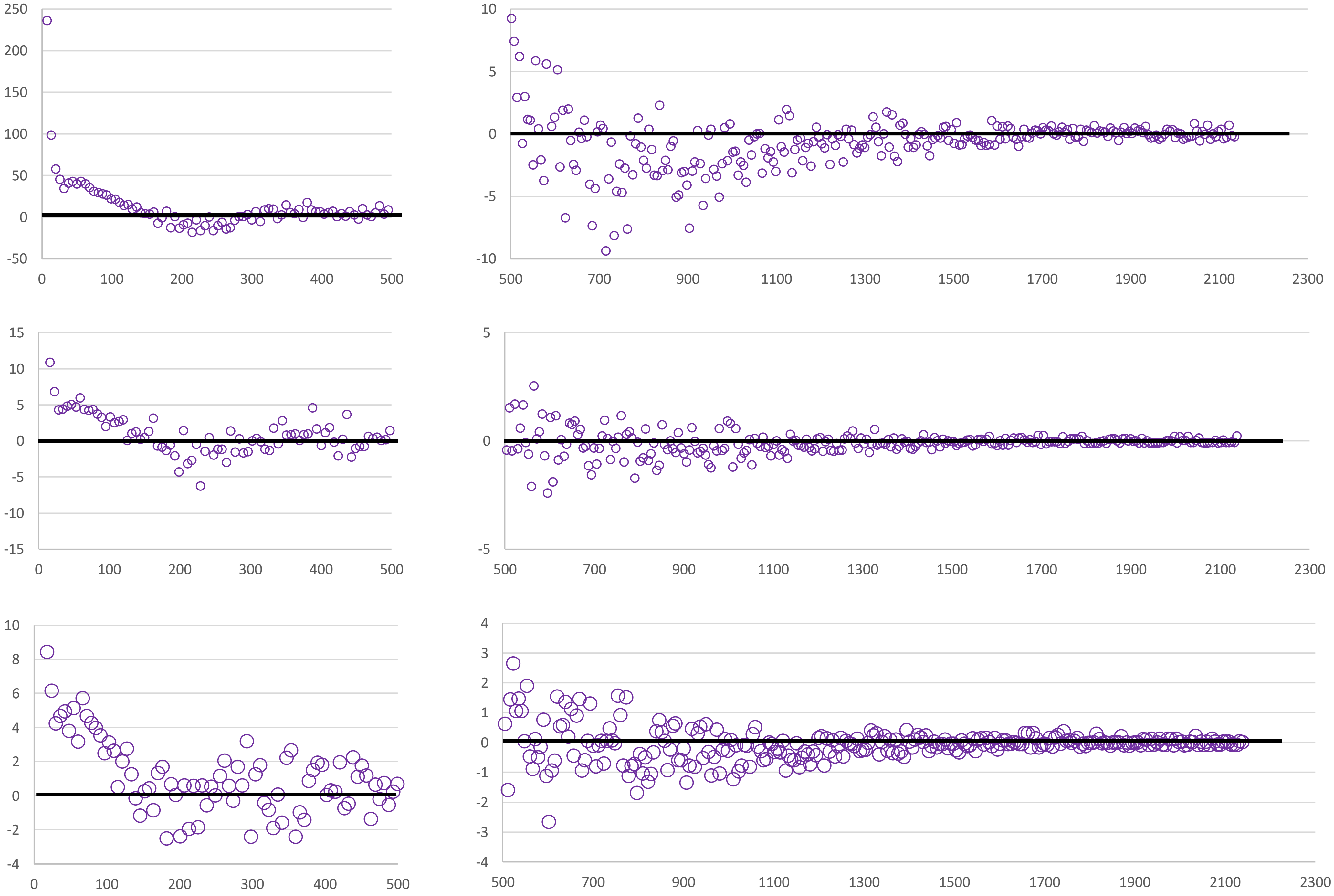

SW_3 (-13.7331 -27.9639)

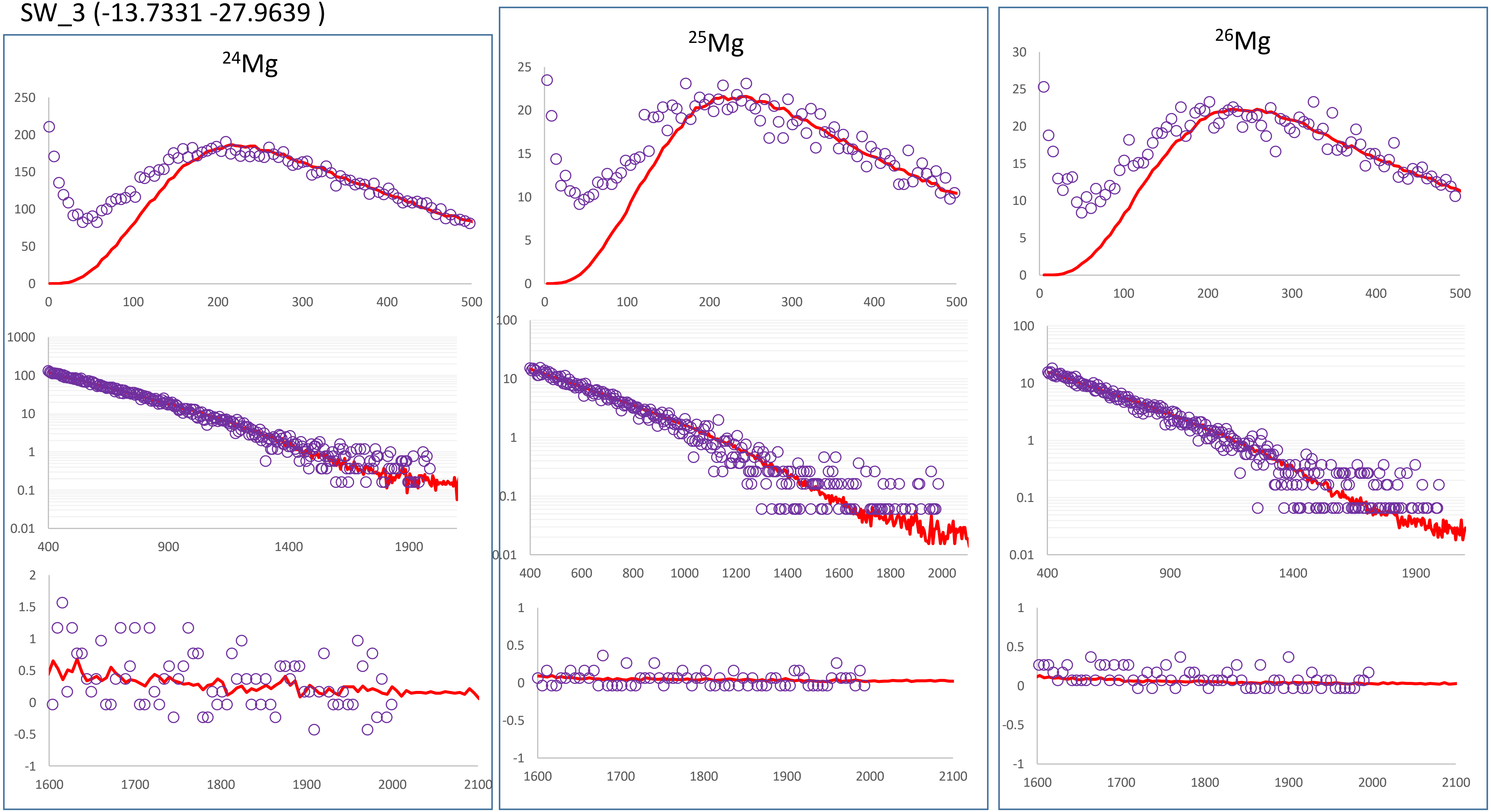

SW_3 (residuals)

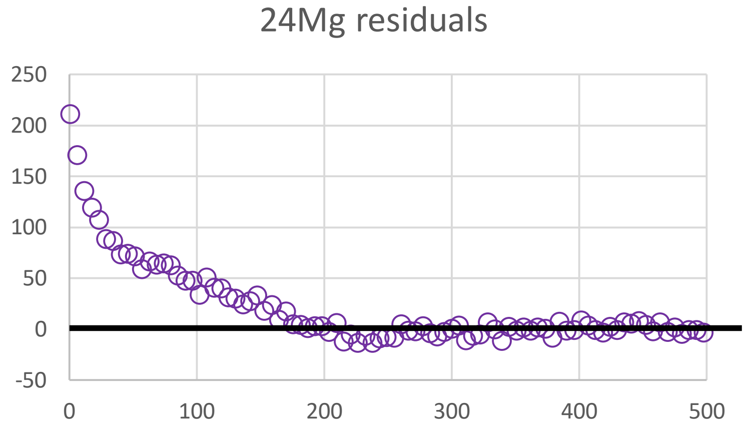

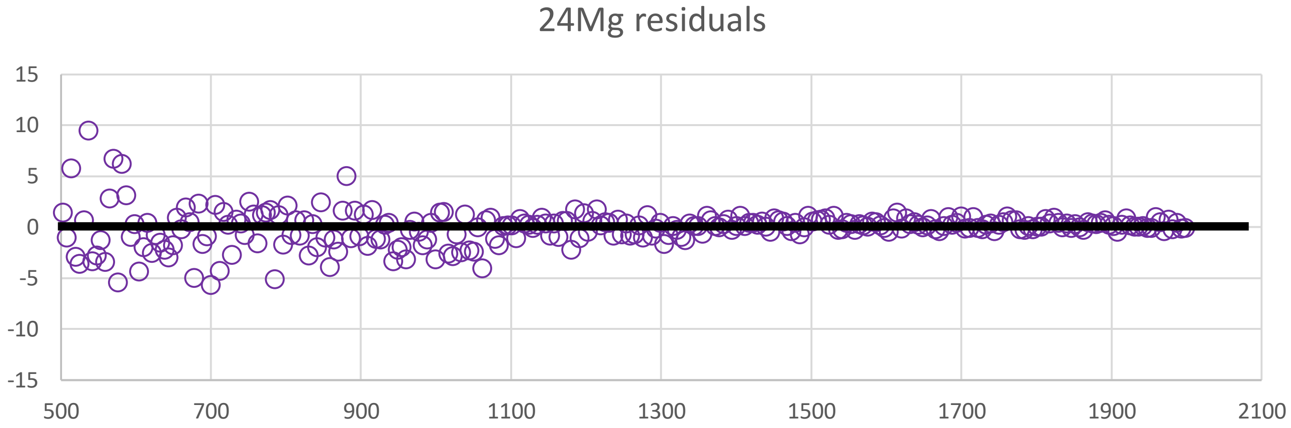

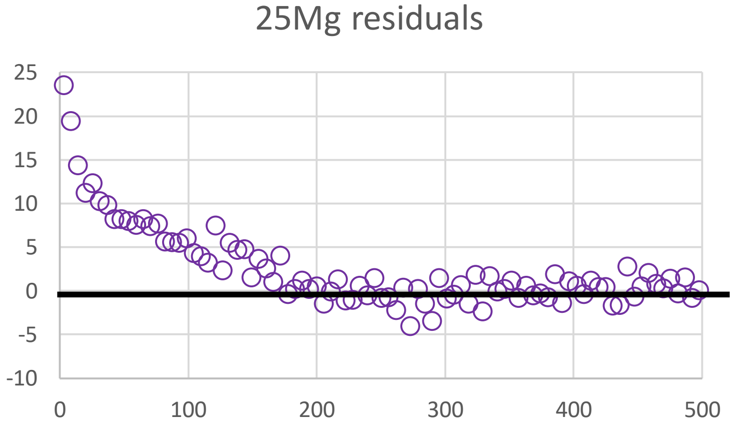

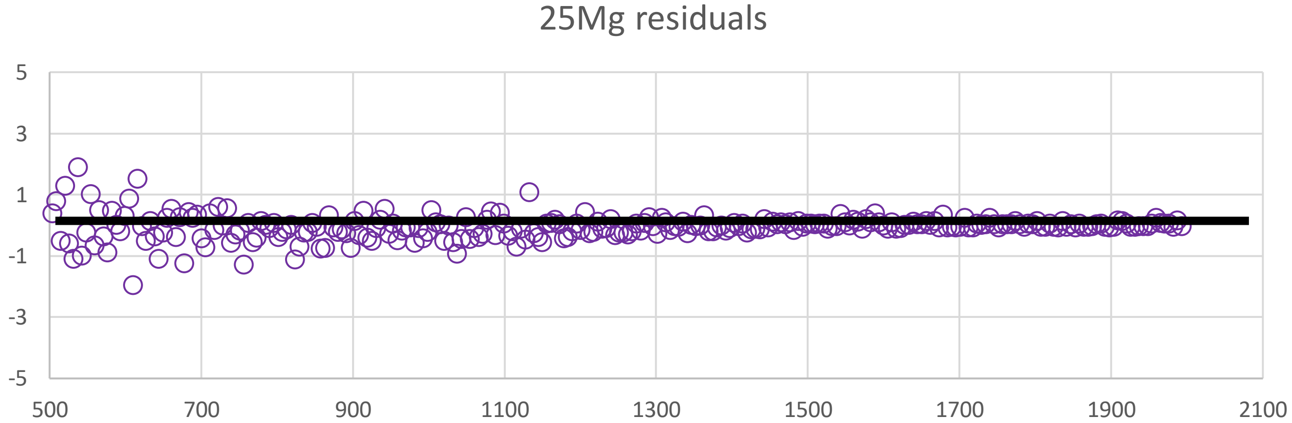

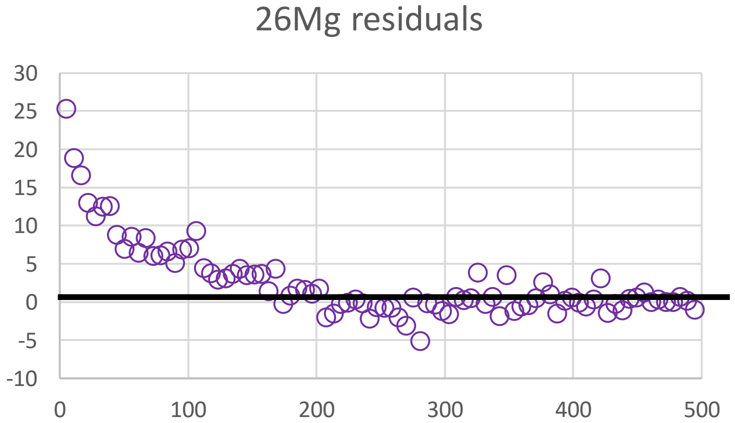

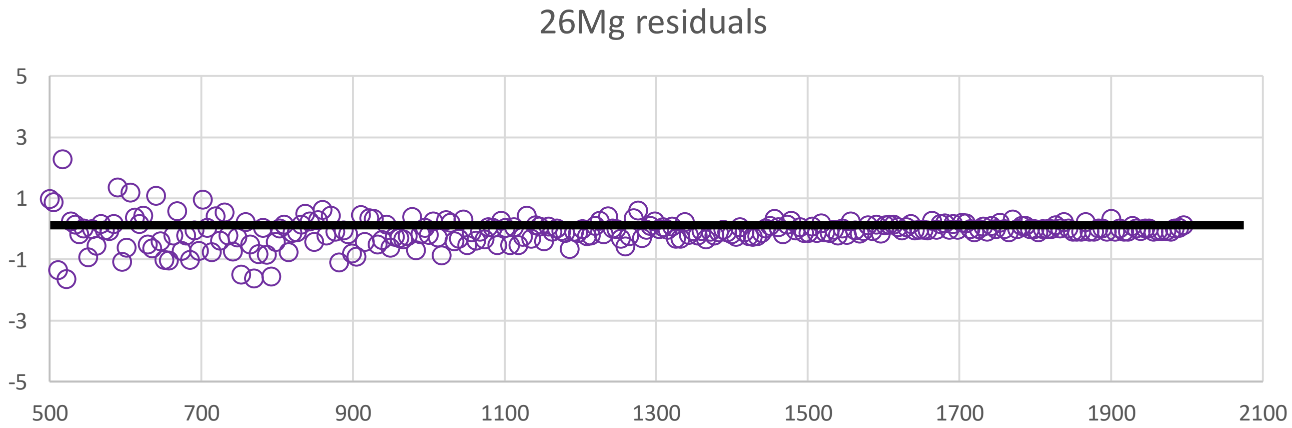

SW_5 (-18.1661 -30.0843)

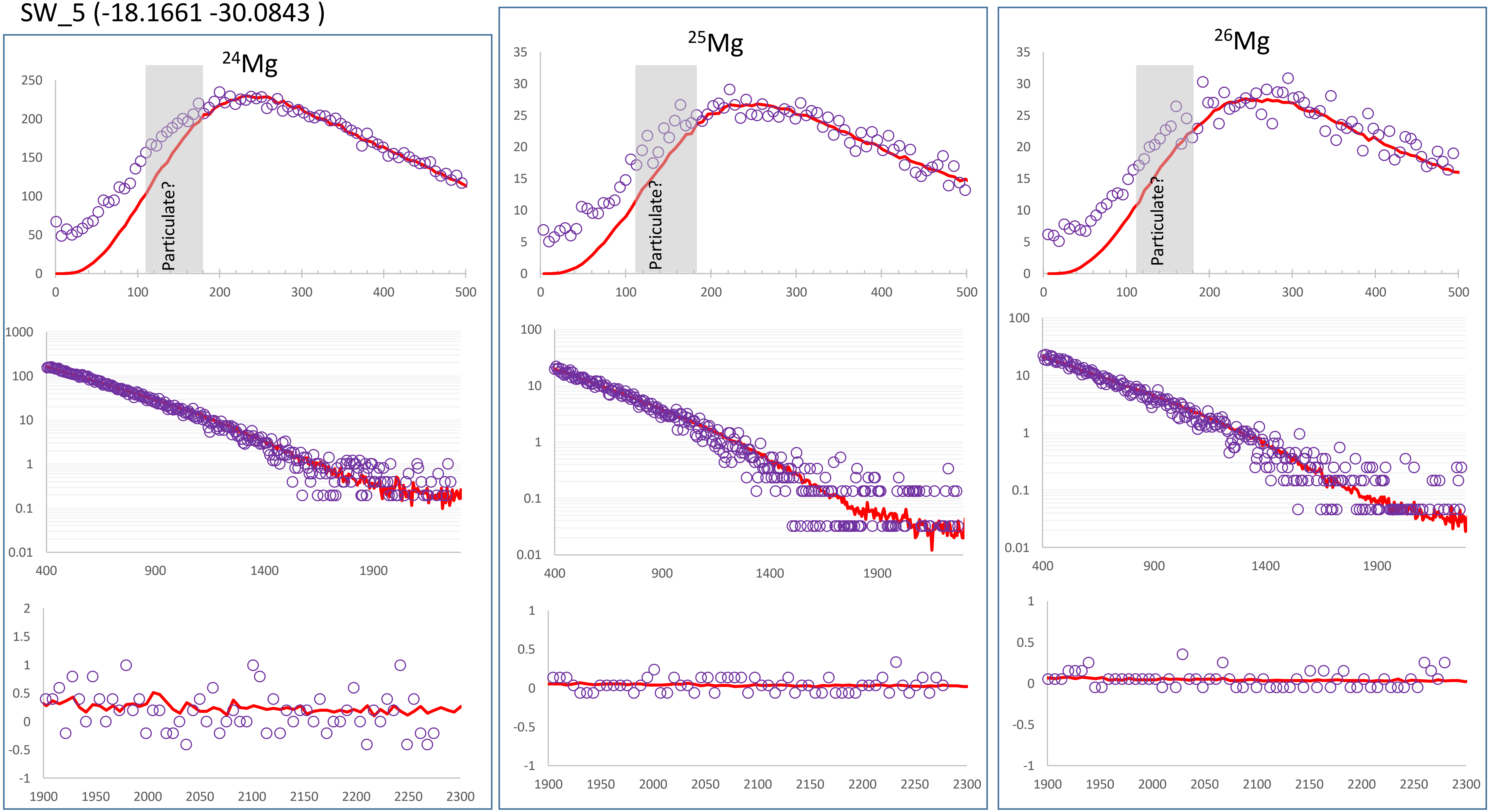

SW_5 (residuals)

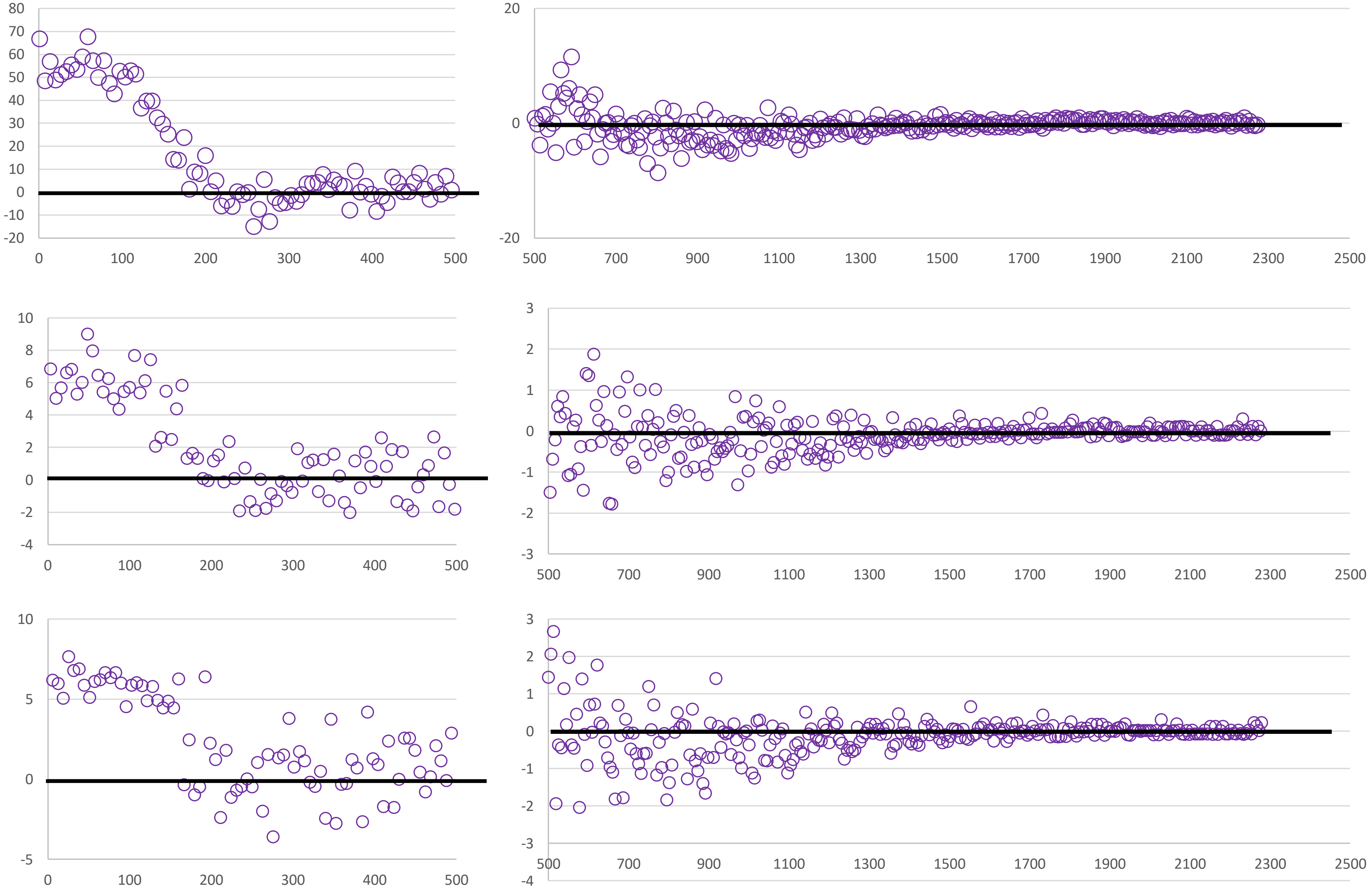

err on sw_6with bkg corr.xlsx - Excel

SW_6 (-0.67067 -19.5501)

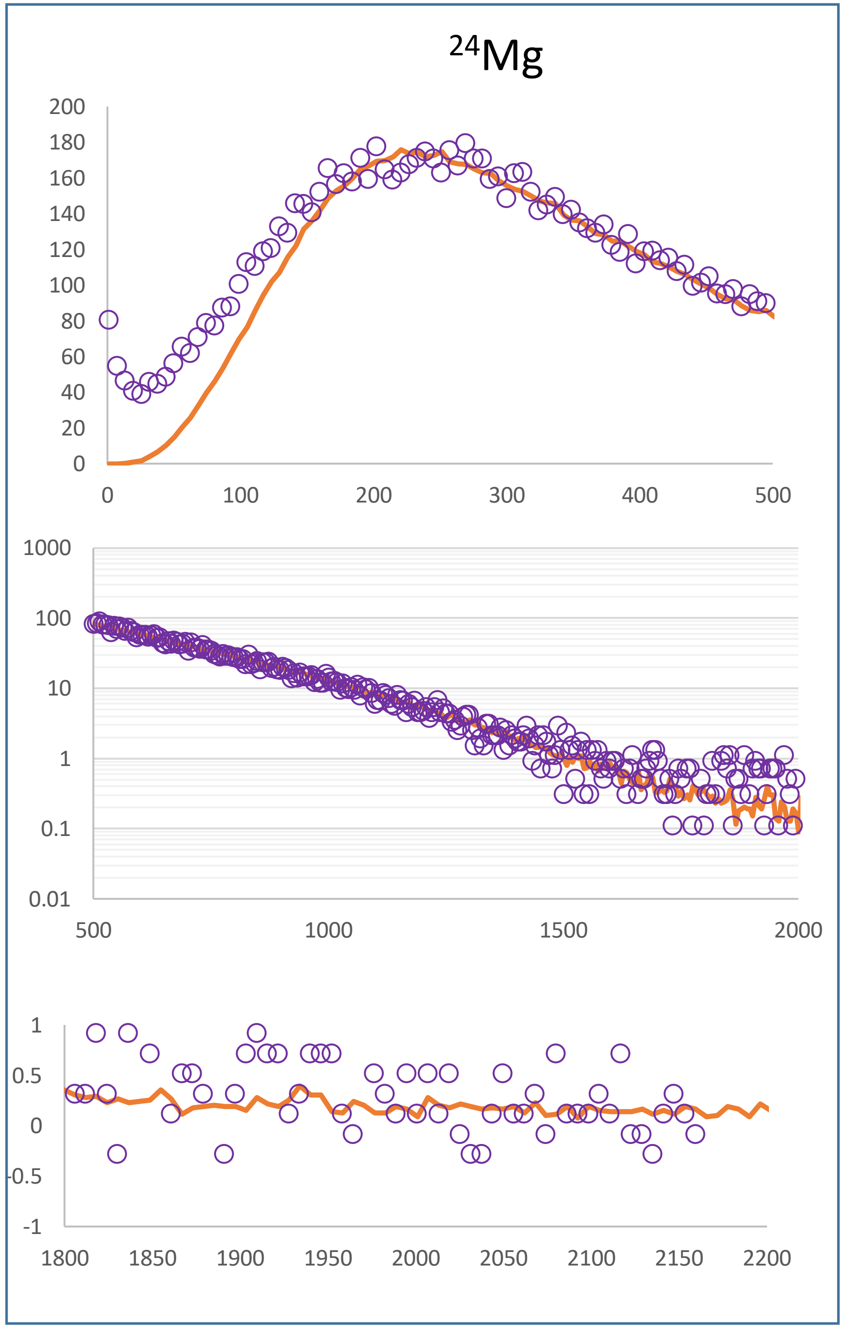

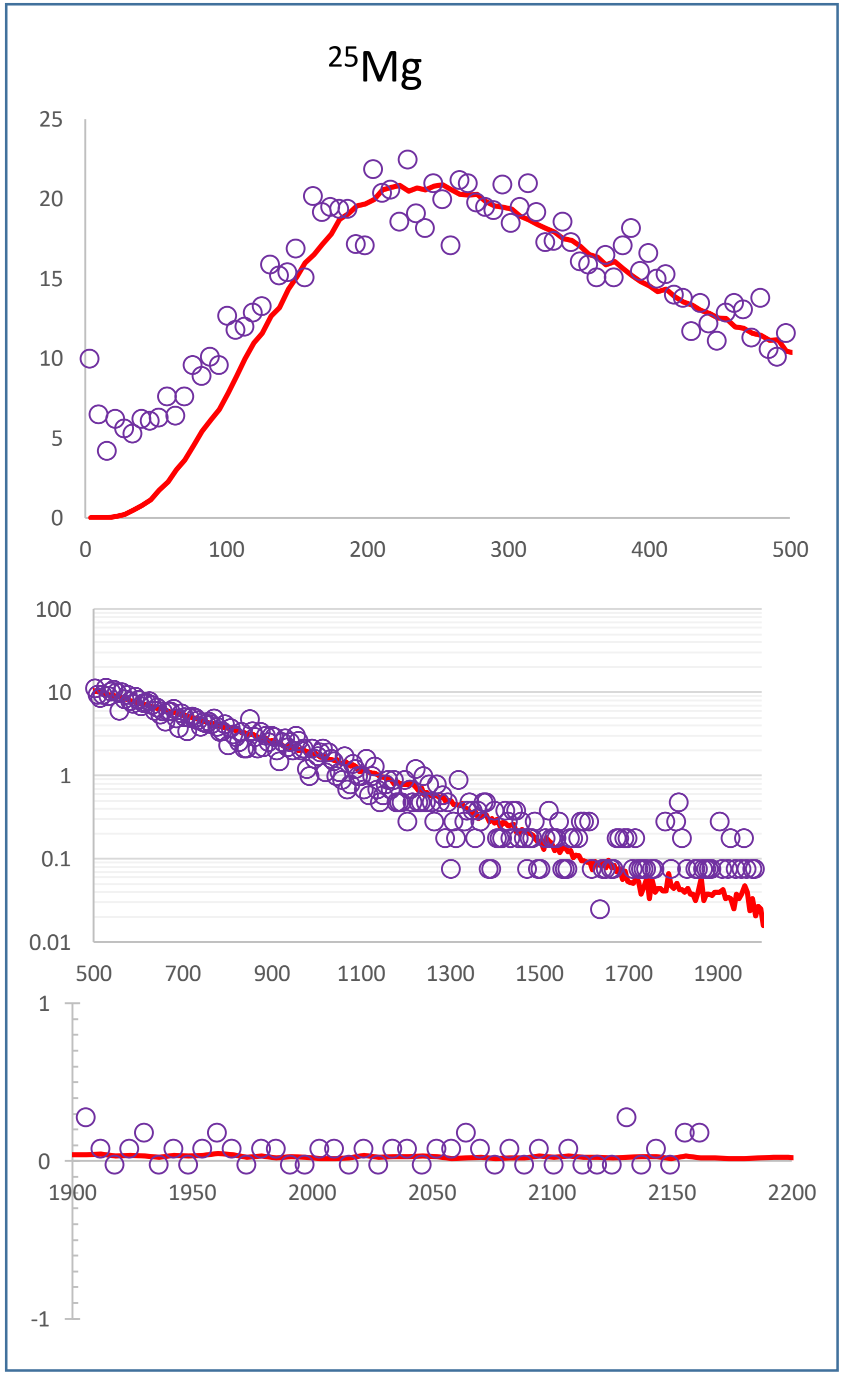

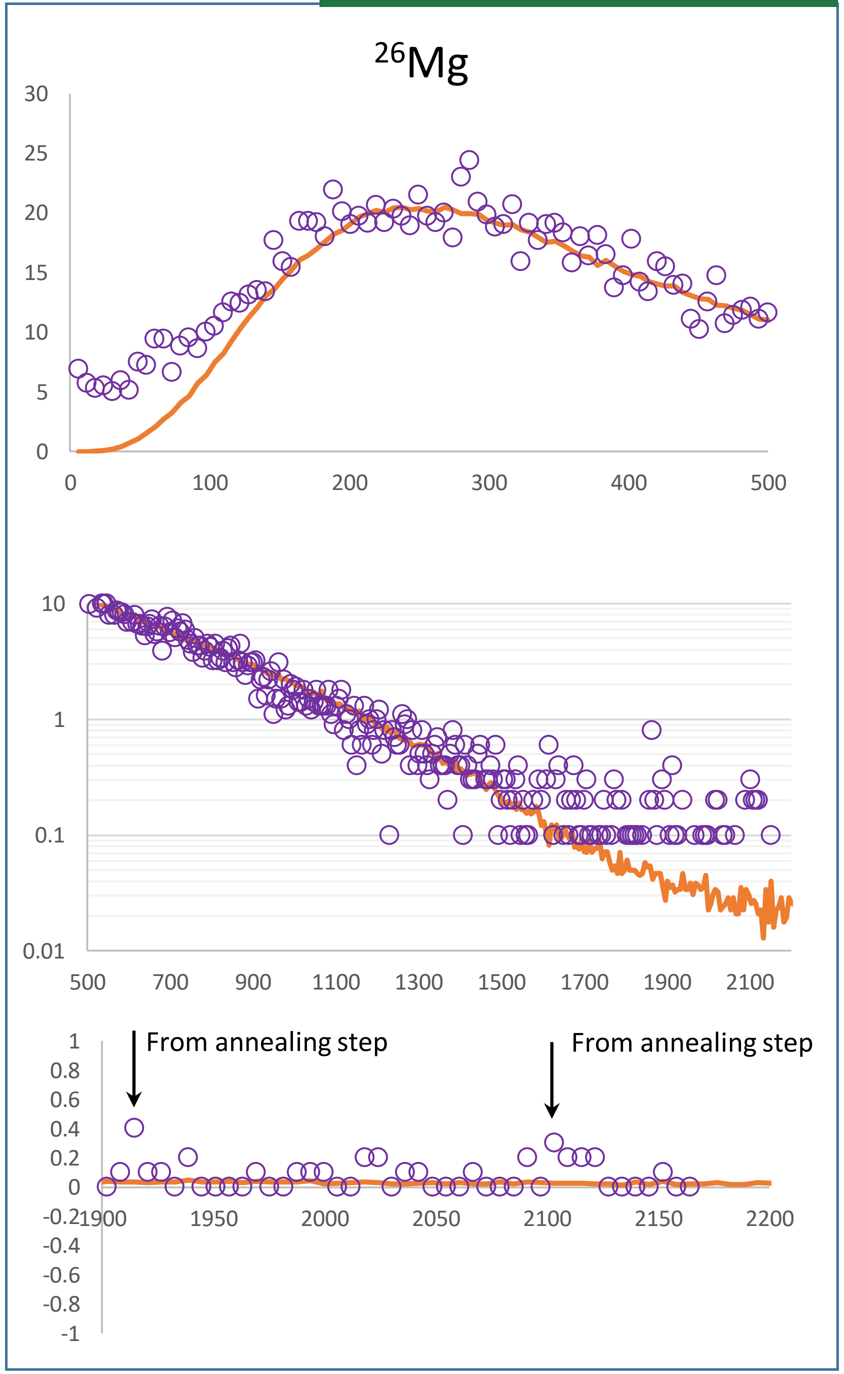

SW_6
(residuals before rework
i.e., -2, -20)

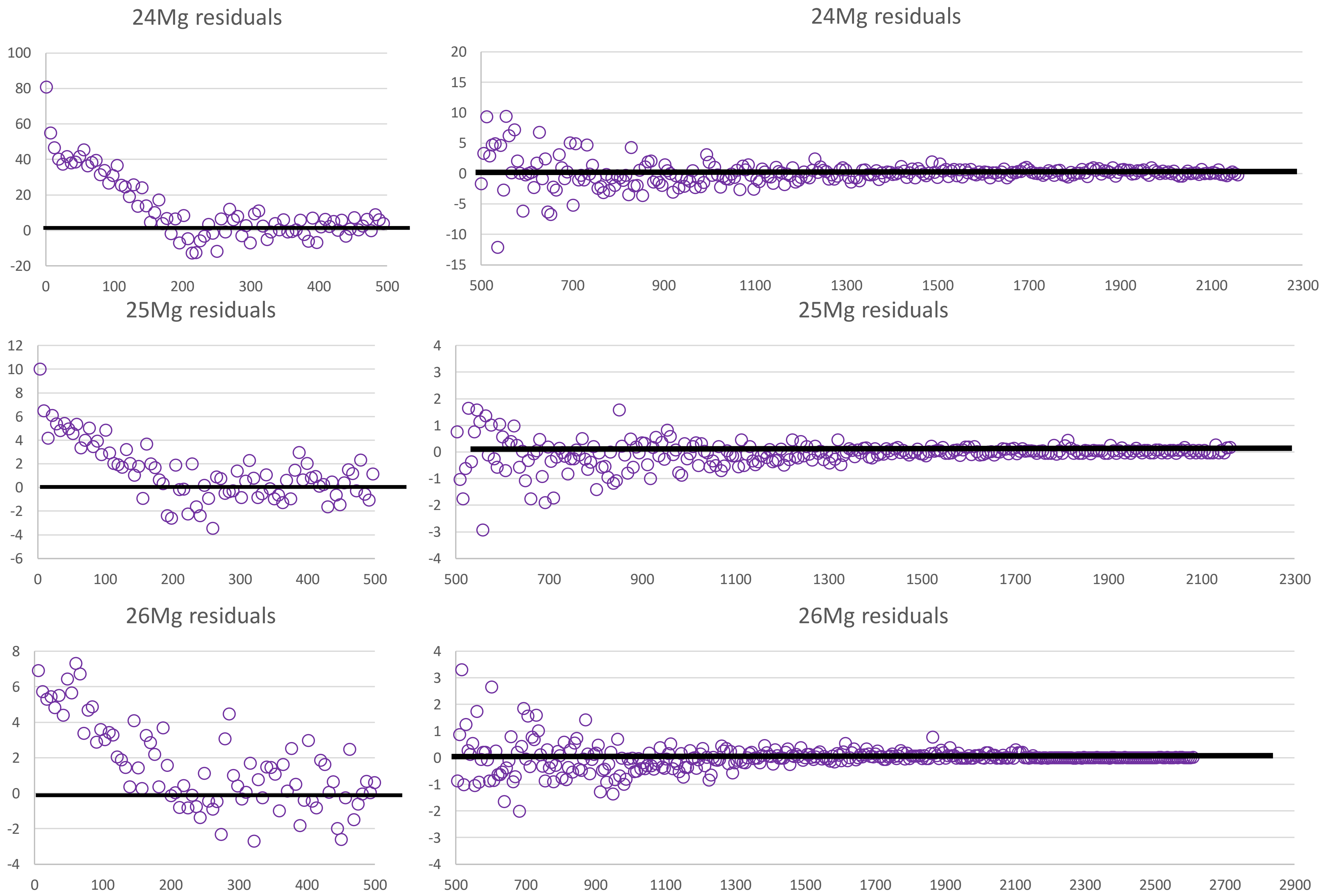

SW_6
(residuals after
rework
i.e. -13, -30)

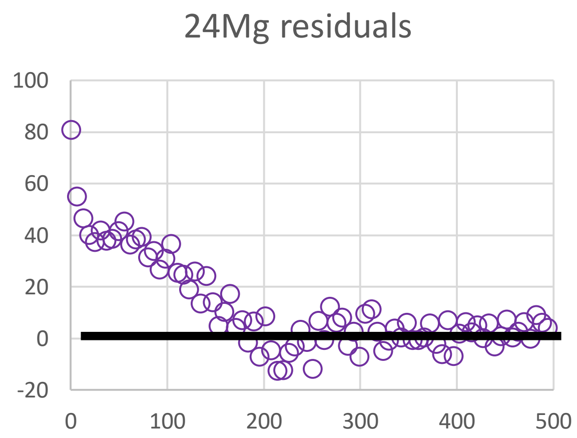

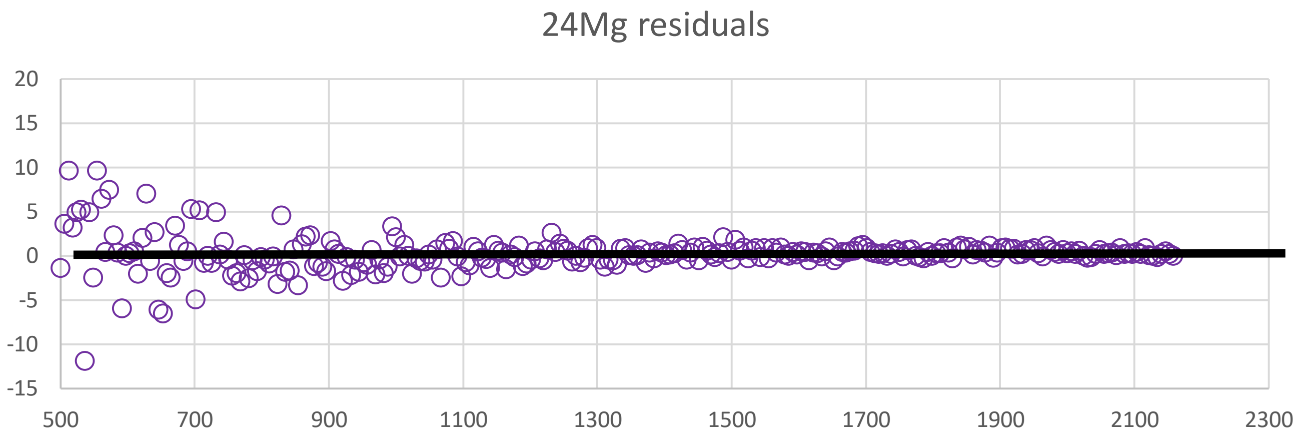

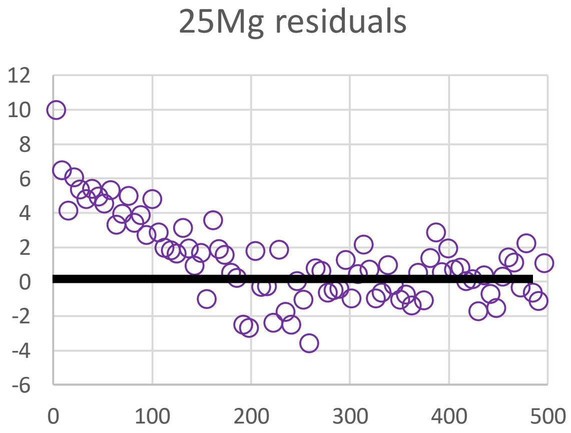

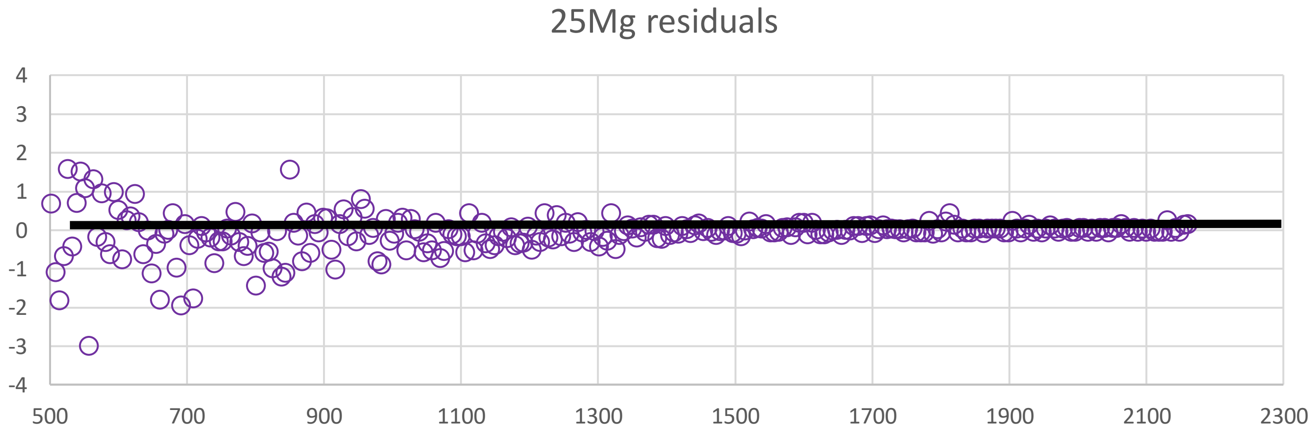

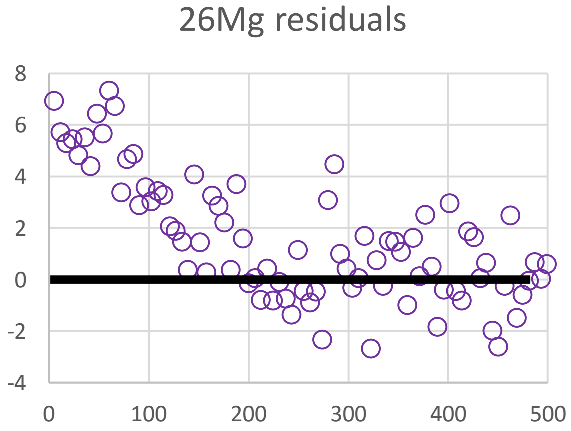

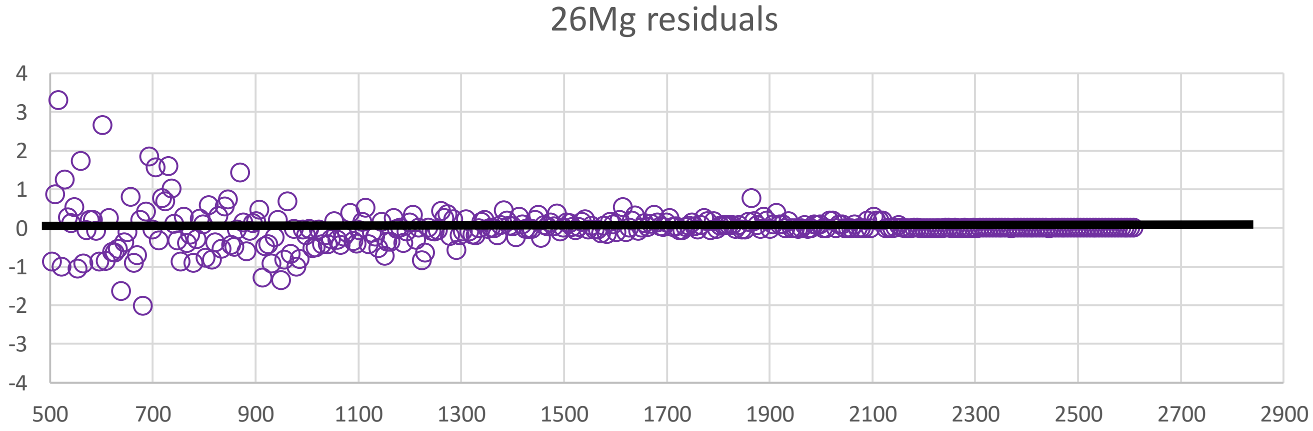

SW_9 (-12.4974, -31.8353)

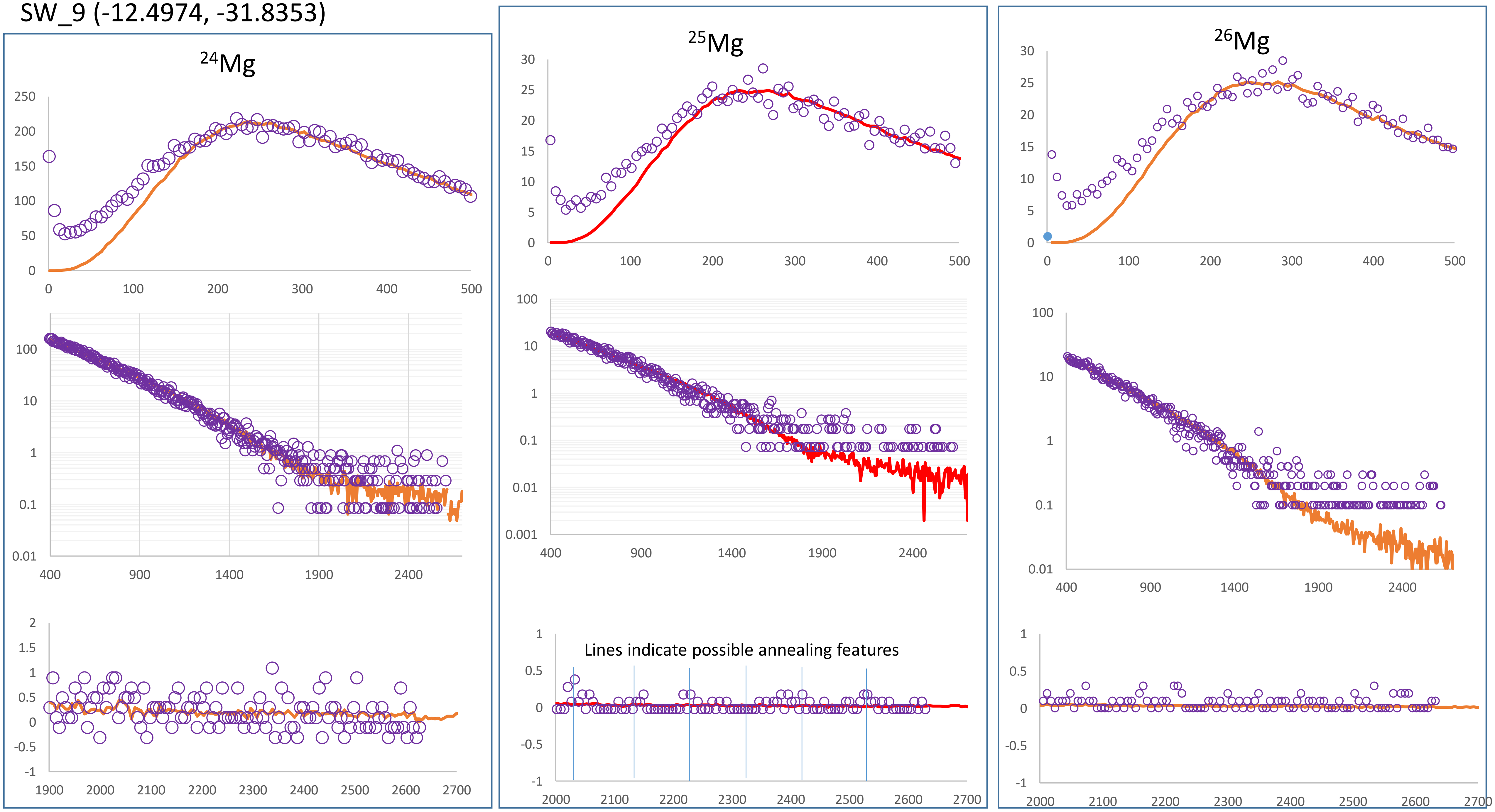

SW_9 (residuals)

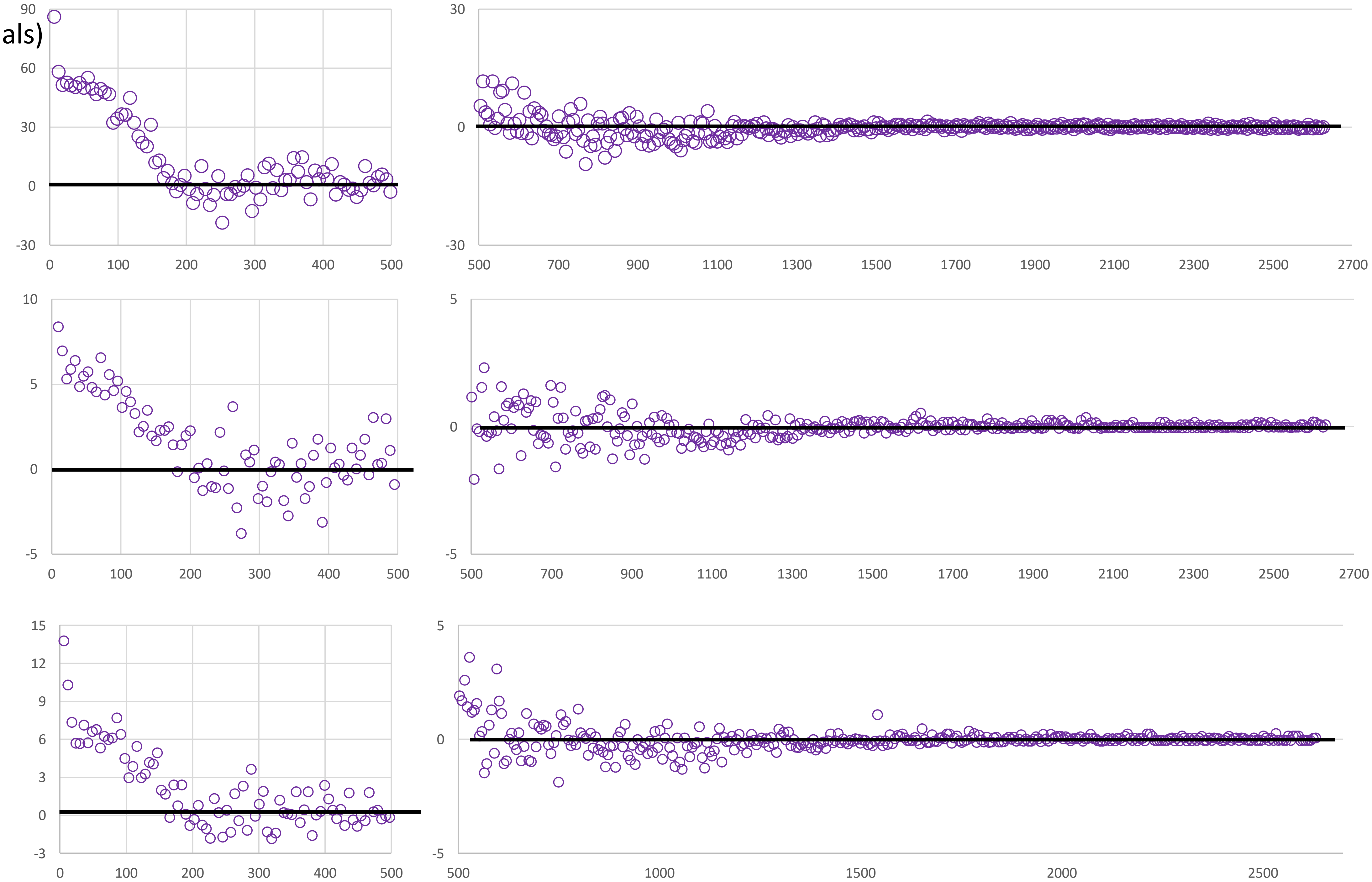

SW_10 (-6.33998 -30.158)

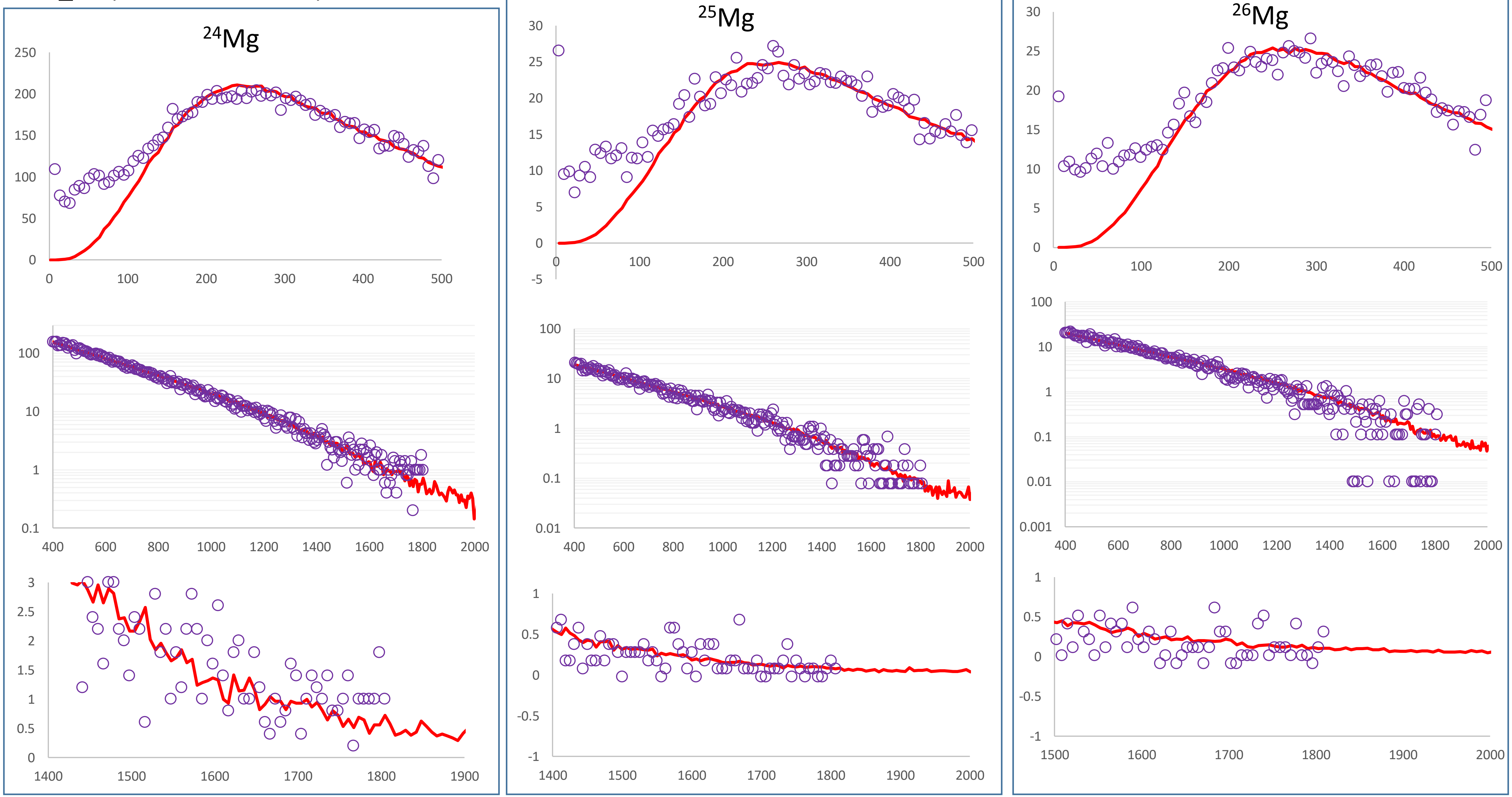

SW_10 (residuals)

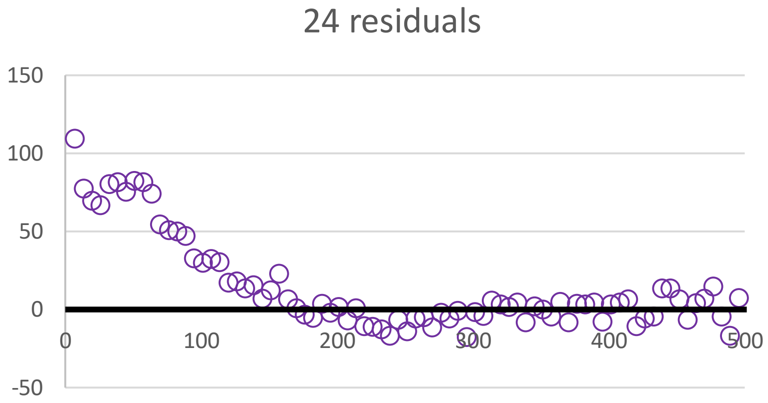

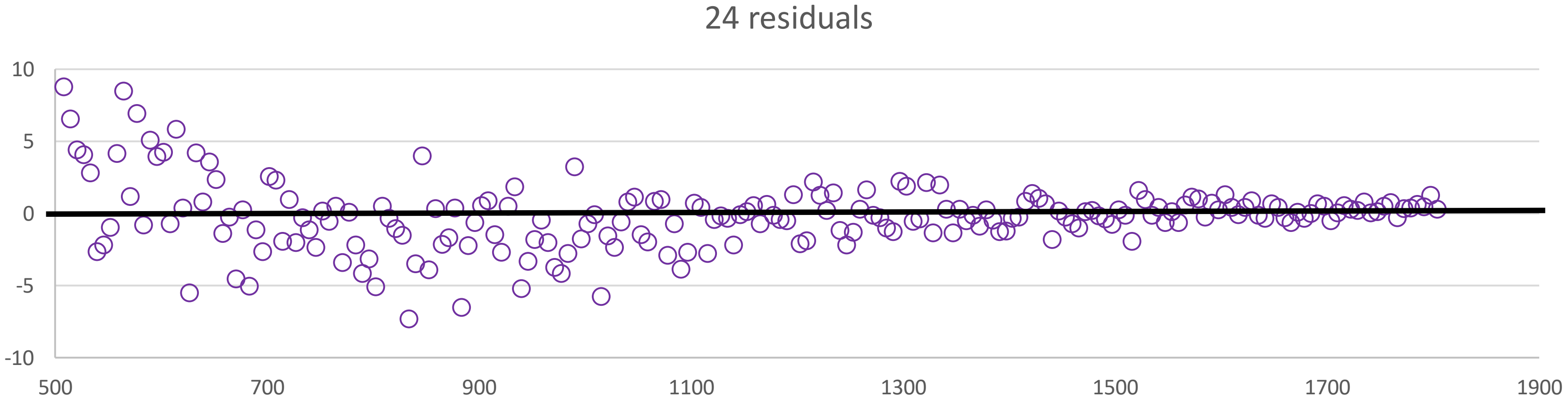

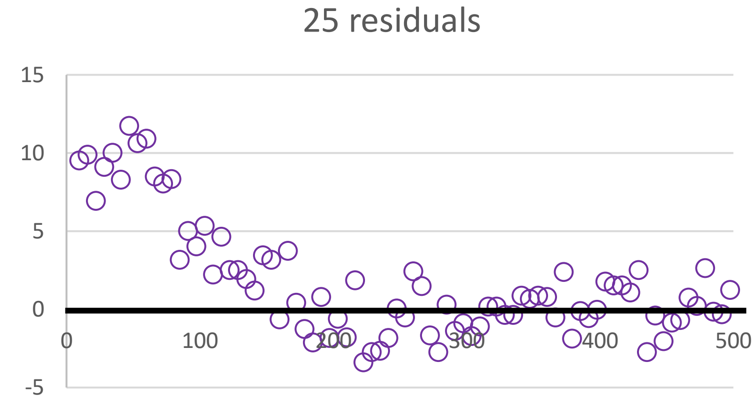

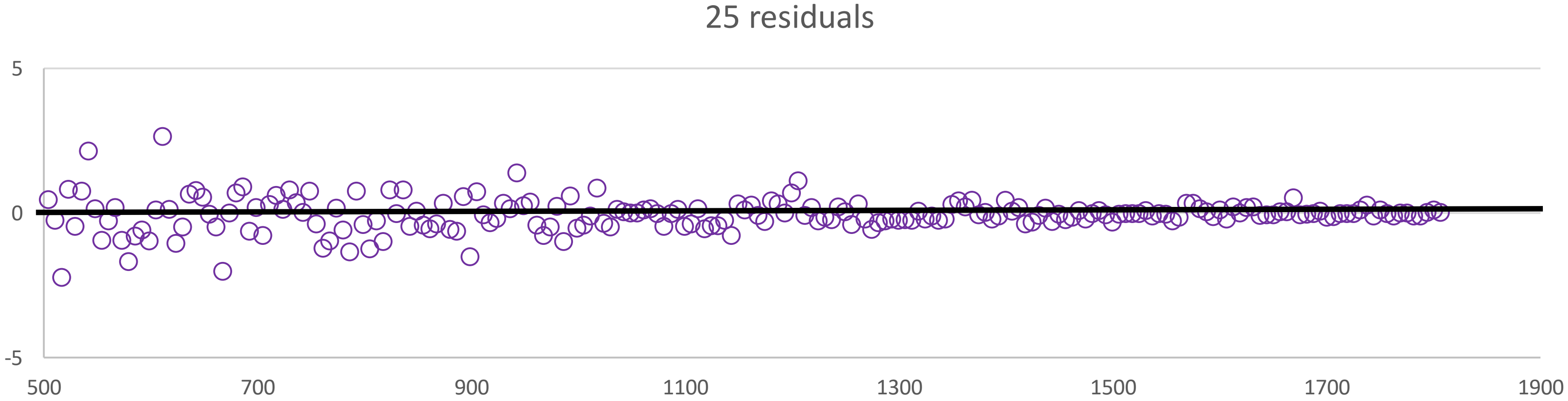

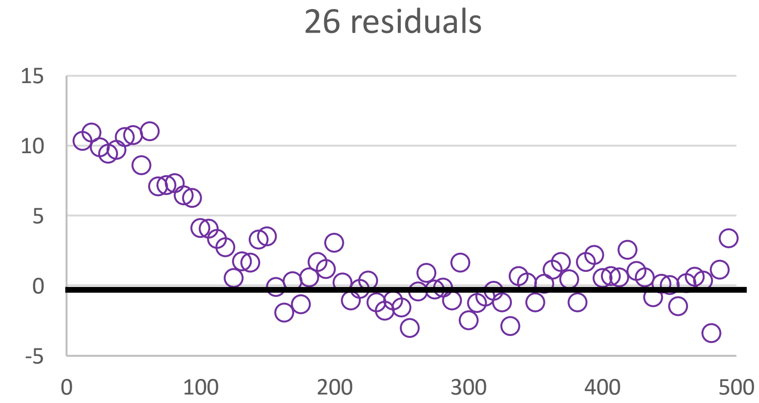

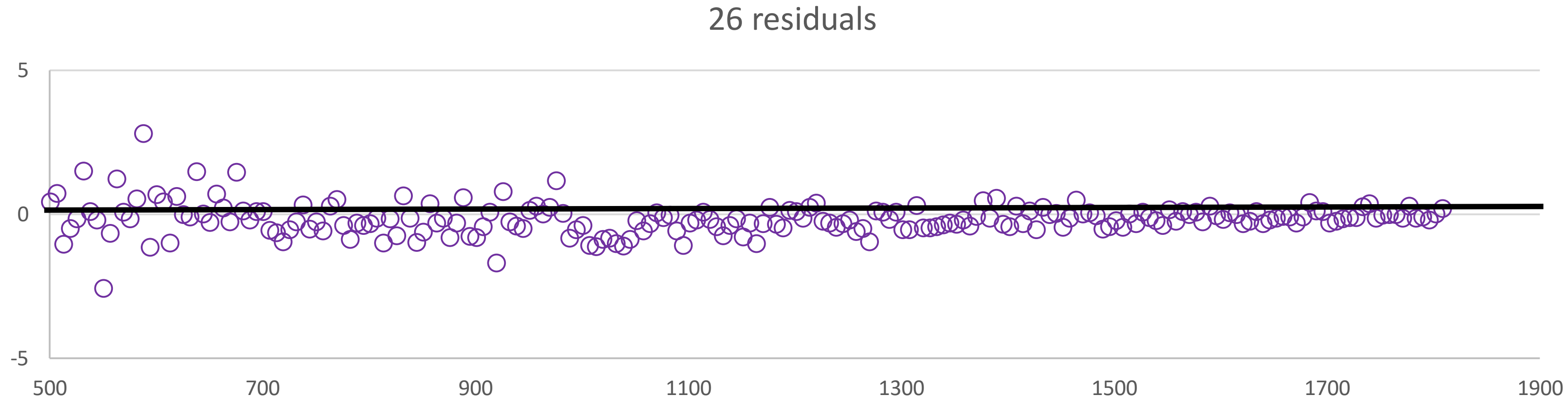

Supplement: Supplementary file 2 — Table A. List of abbreviations in SOM with pointers to more information. A1: Sample preparation procedures. A2: Nature of the interferences. A3: SRIM fits: Determination of surface contamination and background. A4: Correlation of ‰‐level (24MgH)+ with variations in the matrix for SW_2, SW_3, SW_5, SW_6, SW_9, SW_10. A5: References for the A1–A4. A6: Data, plots, and residuals for SRIM fits SW_2, SW_3, SW_5, SW_6, SW_9, SW_10. [file MAPS-55-352-s002.pdf]
